# NaBC1 Boron Transporter Enables Myoblast Response to Substrate Rigidity via Fibronectin‐Binding Integrins

**DOI:** 10.1002/advs.202407548

**Published:** 2025-04-24

**Authors:** Juan Gonzalez‐Valdivieso, Giuseppe Ciccone, Udesh Dhawan, Tezz Quon, Eva Barcelona‐Estaje, Aleixandre Rodrigo‐Navarro, Rafael R. Castillo, Graeme Milligan, Patricia Rico, Manuel Salmeron‐Sanchez

**Affiliations:** ^1^ Centre for the Cellular Microenvironment (CeMi) University of Glasgow Glasgow G11 6EW UK; ^2^ University of Valladolid Valladolid 47002 Spain; ^3^ Institute for Bioengineering of Catalonia (IBEC) The Barcelona Institute for Science and Technology (BIST) Barcelona 08028 Spain; ^4^ Centre for Translational Pharmacology School of Molecular Biosciences College of Medical Veterinary and Life Sciences University of Glasgow Glasgow G12 8QQ UK; ^5^ Universidad de Alcalá Departamento de Química Orgánica y Química Inorgánica Instituto de Investigación Química “Andrés M. del Río” (IQAR) Alcalá de Henares Madrid 28805 Spain; ^6^ Grupo DISCOBAC Instituto de Investigación Sanitaria de Castilla‐La Mancha (IDISCAM) Toledo 45004 Spain; ^7^ Centre for Biomaterials and Tissue Engineering (CBIT) Universitat Politècnica de València Valencia 46022 Spain; ^8^ Biomedical Research Networking Center in Bioengineering Biomaterials and Nanomedicine (CIBER‐BBN) Madrid 28029 Spain; ^9^ Institució Catalana de Recerca i Estudis Avançats (ICREA) Barcelona 08010 Spain

**Keywords:** biomaterials, mechanobiology, mechanotransduction, muscle cells, NaBC1, tissue engineering

## Abstract

Cells are sensitive to the physical properties of their microenvironment and transduce them into biochemical cues that trigger gene expression and alter cell behavior. Numerous proteins, including integrins, are involved in these mechanotransductive events. Here, a novel role for the boron transporter NaBC1 is identified as a mechanotransducer. It is demonstrated that soluble boron ions activate NaBC1 to enhance cell adhesion and intracellular tension in C2C12 myoblasts seeded on fibronectin‐functionalized polyacrylamide (PAAm) hydrogels. Retrograde actin flow and traction forces exerted by these cells are significantly increased in vitro in response to both increased boron concentration and hydrogel stiffness. These effects are fibronectin and NaBC1‐mediated as they are abrogated in hydrogels coated with laminin‐111 in place of fibronectin and in esiRNA NaBC1‐silenced cells. These findings thus demonstrate that NaBC1 controls boron homeostasis and also functions as a mechanosensor.

## Graphical abstract

Cells transduce the rigidity of the environment into biochemical cues through mechanotransductive cascades that modulate cell behavior. Here, a novel role for the boron transporter NaBC1 is identified as a mechanotransducer involved in regulating cell behavior via specific crosstalk with fibronectin‐binding integrins.

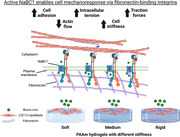

## 1 Introduction

Cells sense mechanical signals from the surrounding environment, the extracellular matrix (ECM), and transduce them into biochemical signals through mechanotransductive processes^[^ ^1^, ^2^ ^]^ While attaching to their underlying matrix, cells probe it to sense its stiffness, viscosity, and topography.^[^ ^3^, ^4^, ^5^, ^6^ ^]^ Cells then bind to the ECM via integrins, specifically to ECM proteins through so‐called focal adhesions (FA).^[^ ^7^, ^8^ ^]^ The molecular clutch model^[^ ^9^, ^10^, ^11^ ^]^ explains how cells mechanically sense the ECM by strengthening proteins involved in the mechanical links between the ECM, integrins, and their actin cytoskeleton. These physical links involve other mechanosensitive proteins found in FAs that can bind directly to actin, including talin and vinculin.^[^ ^10^, ^12^, ^13^, ^14^ ^]^ The adhesion machinery these proteins create allows cells to exert forces on the substrate, which, in turn, allows them to migrate, proliferate, and even differentiate into multiple cell types.^[^ ^4^, ^15^, ^16^, ^17^, ^18^, ^19^ ^]^ The molecular clutch is a well‐accepted paradigm. However, recent studies suggest that other membrane proteins might play important roles in cell mechanotransduction^[^ ^20^, ^21^ ^]^ by helping to modulate the strength of cell adhesion and the transmission of forces between cells and the ECM, thereby enabling the dynamic regulation of cell behavior.

The NaBC1 boron (B) transporter is encoded by the *SLC4A11* gene and is a Na^+^‐coupled B co‐transporter that controls B homeostasis.^[^ ^22^ ^]^ Mutations in the *SLC4A11* gene are involved in rare diseases, such as endothelial corneal dystrophies.^[^ ^23^ ^]^ Previous studies have reported a role for B in osteogenic differentiation^[^ ^24^ ^]^ and adipogenesis inhibition^[^ ^25^ ^]^; however, B function and homeostasis are not completely understood. We have previously demonstrated that NaBC1 crosstalk with growth factor receptors (GFR) enhances vascularization,^[^ ^26^ ^]^ adhesion‐driven osteogenesis,^[^ ^27^ ^]^ myogenic differentiation,^[^ ^28^ ^]^ and muscle regeneration in vivo.^[^ ^29^ ^]^

In this work, we demonstrate that NaBC1 is a mechanosensitive protein and that mechanotransduction happens via its interaction with fibronectin‐binding integrins. We show that active NaBC1 in C2C12 myoblasts enhanced cell spreading in vitro through the formation of more and larger FAs in response to substrate stiffness. We also show that intracellular tension, cell stiffness, retrograde actin flow, and traction forces are upregulated by B in a substrate‐stiffness‐dependent manner. From our findings, we propose that NaBC1 is a mechanosensor that plays an important role in cell response to mechanical stimuli. This new role for NaBC1 might be important for understanding the pathologies that occur in skeletal muscle when cell cytoskeleton‐ECM interactions are disrupted to cause muscular dystrophies.^[^ ^30^ ^]^

## 2 Results and Discussion

### 2.1 Active NaBC1 Modulates Cell Response to Substrate Rigidity

Polyacrylamide (PAAm) hydrogels with tuneable properties are well‐known systems that are widely used to study cell‐ECM interactions to investigate processes such as cell migration, proliferation, malignancy, and differentiation.^[^ ^31^ ^]^ The stiffness of the ECM can activate different intracellular pathways and cytoskeletal arrangements, which modulate cell responses through the integrin receptors in the mechanotransductive process.^[^ ^32^ ^]^ Here, we investigate whether there are other, as yet unidentified, cell receptors and proteins that play a role in mechanotransduction.

**Figure 1:**
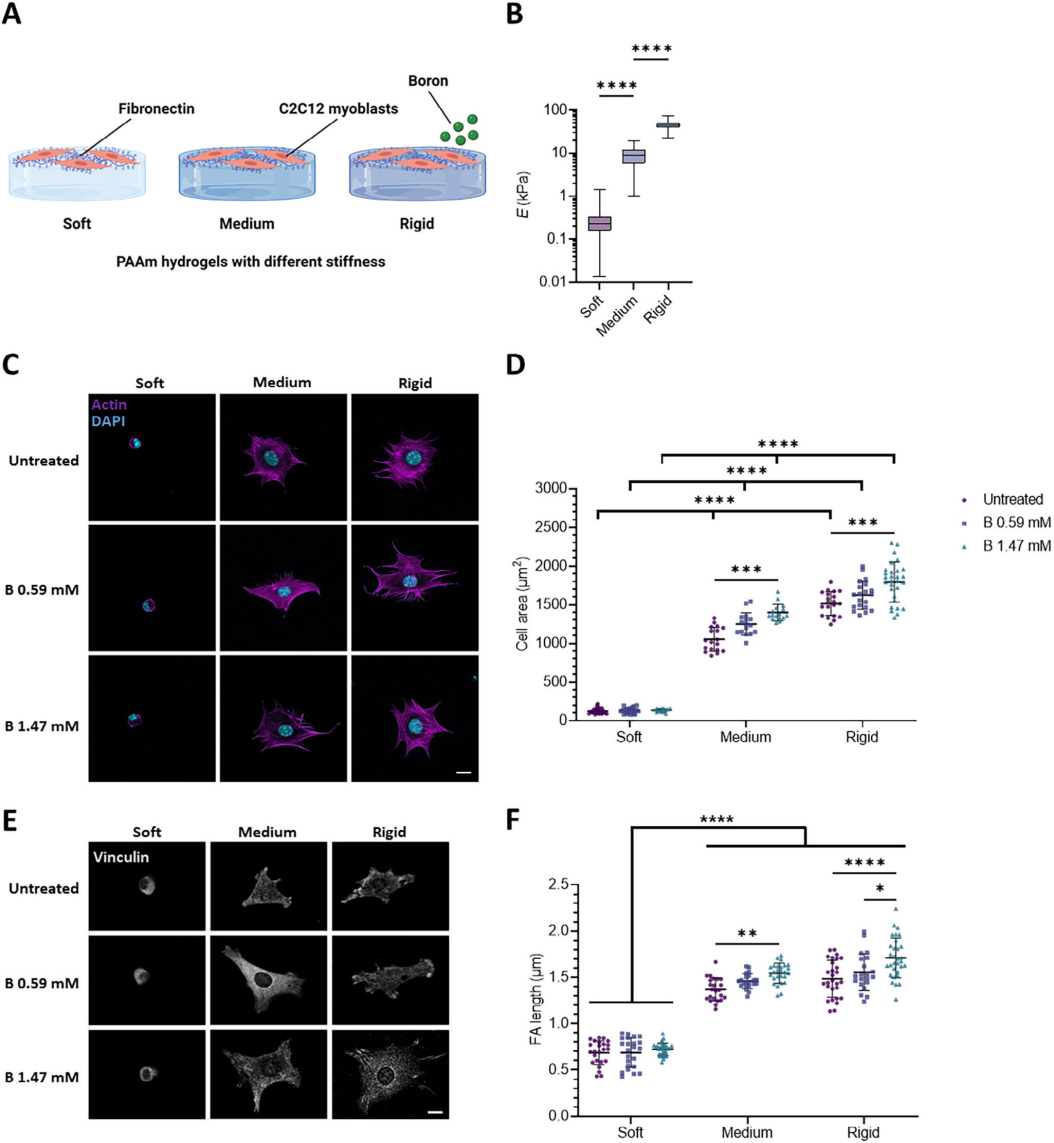
Active NaBC1 modulates cell response to substrate rigidity. A) Schematic representation of C2C12 myoblasts seeded on PAAm hydrogels with different mechanical properties (soft, medium, and rigid) functionalized with fibronectin and treated with Boron (B). Scheme created with BioRender.com. B) Measurements of the Young's modulus of each hydrogel (soft, medium, rigid) by nanoindentation. *n* > 3 hydrogels with nine repeated indentations on each single hydrogel. C) Representative immunofluorescence images of C2C12 myoblasts seeded on PAAm hydrogels of different stiffnesses, functionalized with fibronectin, and stimulated with soluble B (0.59 and 1.47 mm). Magenta: actin cytoskeleton; Cyan: DAPI. Scale bar: 20 µm. D) Quantification of projected cell area of C2C12 myoblasts, cultured as described in panel B (0.59 and 1.47 mm). *n* = 10 cells from three different biological replicates. E) Representative immunofluorescence images of C2C12 myoblasts, cultured as described in panel B. Gray: vinculin. Scale bar: 30 µm. F) Quantification of focal adhesion (FA) length in C2C12 myoblasts, cultured as described in panel B (0.59 and 1.47 mm). *n* = 10 cells from three different biological replicates. Data are represented as Mean ± Standard Deviation, and differences are considered significant for *p* ≤ 0.05 using one‐way ANOVA or two‐way ANOVA (Tukey's multiple comparisons tests) for multiple comparisons. ***p ≤ 0.001, ****p ≤ 0.0001.

In this study, we used PAAm hydrogels of different rigidities, as characterized by the Young's modulus (*E*), which we termed soft (*E =*0.5 kPa), medium (*E =*9 kPa), and rigid (*E =*35 kPa) (**Figure** **1**A,B). First, PAAm hydrogels were functionalized with fibronectin to enable cell interactions, and then C2C12 myoblasts were seeded on top of them. We measured these cells’ viability and found it to be > 95% (Figure S1A,B, Supporting Information). The rigidity of the PAAm hydrogel substrate markedly influenced cell spreading and adhesion (Figure 1C,D). On soft hydrogels, C2C12 myoblasts remained round in shape and did not properly attach to the substrate, but on medium and rigid substrates, they spread more and became larger. Substrate rigidity had an important effect on cell spreading since cell area significantly increased on the stiffest substrate (1515 ± 219 µm^2^), compared to cells seeded on medium hydrogels (1053 ± 154 µm^2^). We used concentrations of soluble B that have no effect on the viability of C2C12 myoblasts (Figure S1C, Supporting Information). Even though substrate rigidity had no effect on cell viability, cell proliferation was markedly altered (Figure S1D, Supporting Information). C2C12 myoblasts on soft hydrogels did not proliferate at a normal rate relative to their proliferation on culture plates and on medium or rigid PAAm hydrogels. It is well known that, on soft substrates (0.5 kPa), adherent cells do not proliferate due to a lack of adhesion.^[^ ^33^, ^34^ ^]^

On both medium and rigid substrates, we observed enhanced cell spreading (up to 1400 ± 110 µm^2^ and 1800 ± 260 µm^2^, respectively) when the NaBC1 transporter was stimulated with soluble B in a concentration‐dependent manner, as reported in previous studies conducted on glass.^[^ ^29^ ^]^

Increased substrate rigidity induces the growth of FAs and increases intracellular tension, in agreement with the prediction of the molecular clutch model.^[^ ^10^, ^11^, ^35^ ^]^ We used vinculin immunostaining to quantify the size of FAs. Both the size (Figure 1E,F; Figure S2B, Supporting Information) and the number (Figure S2A, Supporting Information) of FAs significantly increased following NaBC1 stimulation (with 0.59 mm and 1.47 mm concentration of B) on medium and rigid substrates but not on soft ones. We note that these differences in cell adhesion to the substrate were due only to substrate rigidity and not to different densities of fibronectin on the PAAm hydrogels used (Figure S3, Supporting Information).

**Figure 2:**
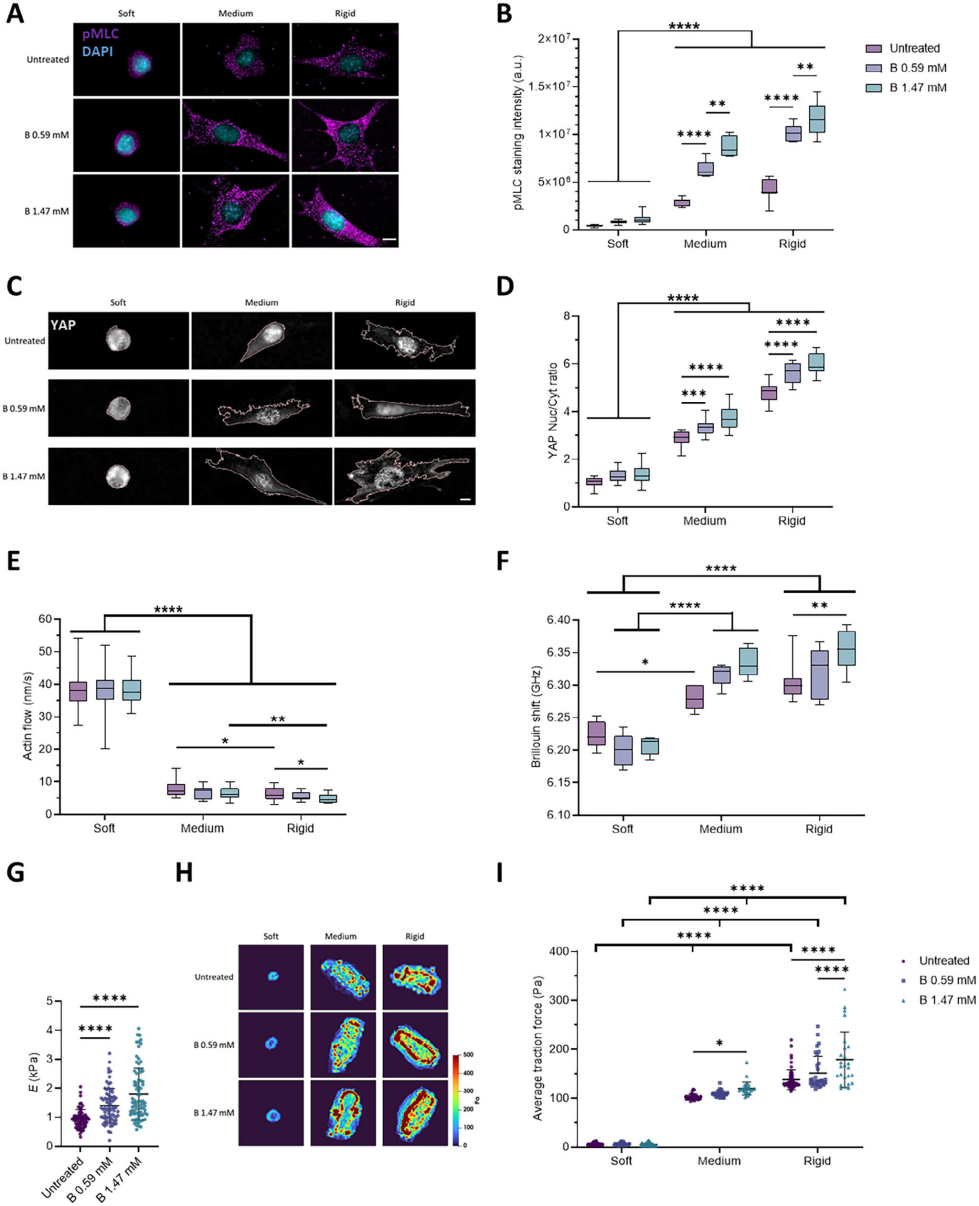
Active NaBC1 enhances intracellular tension, retrograde actin flow, and forces exerted by cells. The experimental results reported in A–I were obtained from culture conditions in which C2C12 myoblasts were seeded on PAAm hydrogels of different stiffnesses (soft, medium, and rigid) that were functionalized with fibronectin (FN) and stimulated with soluble boron (0.59 and 1.47 mm). A) Representative immunofluorescence images of C2C12 myoblasts cultured as described. Magenta: phosphorylated myosin light chain (pMLC); Cyan: DAPI. Scale bar: 50 µm. B) Quantification of pMLC intensity in C2C12 myoblasts cultured as described. *n* = 10 cells from three different biological replicates. C) Representative immunofluorescence images of C2C12 myoblasts cultured as described. Gray: YAP; Light pink: cell outline. Scale bar: 50 µm. D) Quantification of YAP nuclear translocation in C2C12 myoblasts cultured as described. *n* = 10 cells from 3 different biological replicates. E) Quantification of actin retrograde flow in C2C12 myoblasts cultured as described. *n* = 5 cells with at least five different flow areas per cell. F) Quantification of Brillouin shift in C2C12 myoblasts cultured as described and imaged using Brillouin microscopy. *n* = 10 cells from three different biological replicates. G) Quantification of cell stiffness by nanoindentation of C2C12 myoblasts seeded on glass coverslips functionalized with FN and stimulated with soluble B (0.59 and 1.47 mm). *n* = 10 cells with nine indentations on each single cell from three different biological replicates. H) Representative traction maps of C2C12 myoblasts cultured as described. I) Quantification of traction forces exerted by C2C12 myoblasts cultured as described. *n* = 30 cells from ten different locations within each hydrogel from three different biological replicates. Data are represented as Mean ± Standard Deviation, and differences are considered significant for *p* ≤ 0.05 using one‐way ANOVA or two‐way ANOVA (Tukey's multiple comparisons tests) for multiple comparisons. *p ≤ 0.05, **p ≤ 0.01, ***p ≤ 0.001, ****p ≤ 0.0001.

The Yes‐associated protein (YAP) is a reporter for mechanotransduction.^[^ ^14^ ^]^ Phosphorylation of the myosin light chain (pMLC) regulates cytokinesis and plays an important role in diverse cell functions via a Rho‐associated kinase (ROCK) pathway after the assembly of FAs.^[^ ^36^ ^]^ To determine whether the effect of NaBC1 on cell adhesion involves intracellular tension, we measured the phosphorylation of MLC (**Figure** **2**A,B) and YAP nuclear translocation (Figure 2C,D). Our results show that both pMLC levels and YAP nuclear translocation were significantly increased on medium (up to 2.88 and 3.72‐fold increase) and rigid (up to 2.79 and 5.98‐fold increase) PAAm substrates after NaBC1 stimulation with soluble B. In cells seeded on soft gels, we observed no significant differences in pMLC levels nor in YAP nuclear translocation following NaBC1 stimulation, regardless of the concentration of B used.

The molecular clutch model links the retrograde flow of actin to the ECM through FAs and integrins. In this model, the elastic resistance of the substrate to deformation offsets the contractility of myosin, thereby slowing the actin flow and increasing the forces loaded onto integrins and FAs. The force loading rate increases with substrate rigidity.^[^ ^9^, ^10^ ^]^ On soft substrates, cells present a poor cytoskeletal organization and weakly assembled FAs (in which talin is folded and vinculin is not recruited), leading to a lack of force generation. As rigidity increases, the cells’ connection to the ECM becomes stronger, giving them time to load enough force onto the FAs. This force loading leads to the unfolding of talin and to the recruitment of vinculin, which enables the actin‐FAs‐integrin‐ECM clutch. To test the role of NaBC1 in this context, actin flow was measured in live C2C12 myoblasts transfected with Lifeact. As expected, the retrograde actin flow decreased as substrate rigidity increased (Figure 2E and Supplementary videos). Upon stimulation of NaBC1 with B, retrograde actin flow decreased in cells seeded on medium substrates (7.7, 7.5 and 6.2 nm s^−1^ for untreated, 0.59 and 1.47 mm of B, respectively), and decreased further in myoblasts seeded on rigid substrates (5.9, 5 and 4.8 nm s^−1^ for untreated, 0.59 and 1.47 mm, respectively) compared to cells on hydrogels of the same rigidity not treated with B. We propose that the slower actin flow observed following NaBC1's stimulation with B involves an enhanced engagement of the molecular clutch, as supported by the enhanced cell adhesion recorded on medium and rigid PAAm hydrogels and as shown in Figure 1. Together, these results suggest that NaBC1 might be linked to the molecular clutch.

Clutch engagement is linked to cytoskeletal tension,^[^ ^37^ ^]^ a major predictor of cell stiffness. We, therefore, measured cell stiffness using Brillouin microscopy (Figure 2F). This is a contact‐free, label‐free, noninvasive technique used to optically map the mechanical properties of biological materials.^[^ ^38^, ^39^, ^40^, ^41^ ^]^ Brillouin microscopy has also been recently used to map the elastic properties of cancer cells on PAAm gels.^[^ ^42^ ^]^ Here, we used Brillouin microscopy to quantify the Brillouin shift (a proxy for elasticity) of C2C12 myoblasts on PAAm hydrogels—the larger the shift of the Brillouin peak, the higher the stiffness of the measured region.^[^ ^43^ ^]^ The resolution of this technique (< 1 µm) allows for the mapping of cell mechanical properties at several points. Cells on soft hydrogels showed a lower Brillouin shift (6.224 ± 0.021 GHz) compared to cells cultured on medium or rigid hydrogels (6.281 ± 0.019 and 6.305 ± 0.029 GHz, respectively). Interestingly, when NaBC1 was stimulated with 0.59 mm of B, the cells on medium (6.317 ± 0.018 GHz) and rigid hydrogels (6.321 ± 0.035 GHz) stiffened. This stiffening was more evident when C2C12 cells were stimulated with B at 1.47 mm, as the Brillouin shift increased to 6.334 ± 0.022 GHz and 6.353 ± 0.029 GHz on medium and rigid hydrogels, respectively (Figure 2F; Figure S4, Supporting Information). We used nanoindentation to confirm the data obtained from Brillouin microscopy (Figure 2G). C2C12 myoblasts treated with B, either at 0.59 or 1.47 mm, presented with significantly higher Young's modulus (1.31 ± 0.59 and 1.64 ± 0.89 kPa, respectively), compared to untreated cells (0.94 ± 0.31 kPa). Thus, higher cell stiffness is a consequence of NaBC1 stimulation as determined by two independent techniques, and might be related to enhanced cell attachment.

Cells generate traction forces that deform the ECM and engage the molecular clutch.^[^ ^6^ ^]^ We, therefore, performed traction force microscopy (TFM) to measure cell forces on the different substrates and to measure changes in cell force after NaBC1 stimulation (Figure 2H,I). We observed in our TFM results that traction stress gradually increased with substrate rigidity. Stress maps indicate that higher forces are exerted on the cell edges, particularly on the stiffest surface (35 kPa), and consistent with the location of large FA complexes (Figure 1E,F). Our results also showed that, following NaBC1 stimulation, the lowest B concentration (0.59 mm) was sufficient to produce a significant increase in cell traction forces (151.1 ± 34.5 Pa) but only on the rigid substrate. When the concentration of B was increased to 1.47 mm, we observed increased cell traction forces on both the medium (119.8 ± 13.4 Pa) and rigid (178.9 ± 56.5 Pa) hydrogels. These results, together with the increased cell stiffness observed following B stimulation, indicate that higher intracellular tension occurs as a result of integrin and NaBC1 activation.

As explained above, cells seeded on soft PAAm hydrogels (*E* = 0.5 kPa) adopted a different cell morphology to cells cultured on stiffer substrates and showed different cell behaviors and proliferation rates as well. We, therefore, hypothesized that C2C12 myoblasts undergo cell senescence due to their lack of adhesion to the underlying substrate. Cell senescence consists of a state in which cells remain metabolically active without undergoing cell death or division.^[^ ^44^ ^]^ It is involved in numerous biological processes, such as tumor suppression, tumor progression, aging, and tissue repair.^[^ ^45^ ^]^ Common markers of cell senescence include multinucleated cells, increased vacuolization, morphological changes, and the expression of pH‐dependent β‐galactosidase.^[^ ^46^ ^]^ β‐galactosidase resides in lysosomes and converts β‐galactosides into monosaccharides under acidic pH. Its activity is 100% higher in senescent cells relative to pre‐senescent cells.^[^ ^47^ ^]^ We, therefore, assayed β‐gal activity to test our hypothesis that C2C12 myoblasts cultured on soft substrates undergo senescence (Figure S6, Supporting Information). Our results showed that the enzymatic activity of β‐galactosidase was 5.82 and 5.08 times higher in C2C12 myoblasts seeded on soft hydrogels than in C2C12 myoblasts seeded on medium or rigid substrates, respectively. Interestingly, cell senescence decreased over time in C2C12 myoblasts seeded on soft hydrogels (Figure S6A,B, Supporting Information), which could be explained by ECM secretion from non‐senescent cells. Moreover, NaBC1 stimulation with B had no effect on β‐gal activity on any substrate at early (Figure S6C, Supporting Information) or long (Figure S6D,Supporting Information) time points. We can speculate that this arrest of the cell cycle might be responsible for the lack of response in cell mechanotransduction after NaBC1 stimulation.

### 2.2 NaBC1 Cooperates with Fibronectin‐Binding Integrins to Modulate Intracellular Signaling

**Figure 3:**
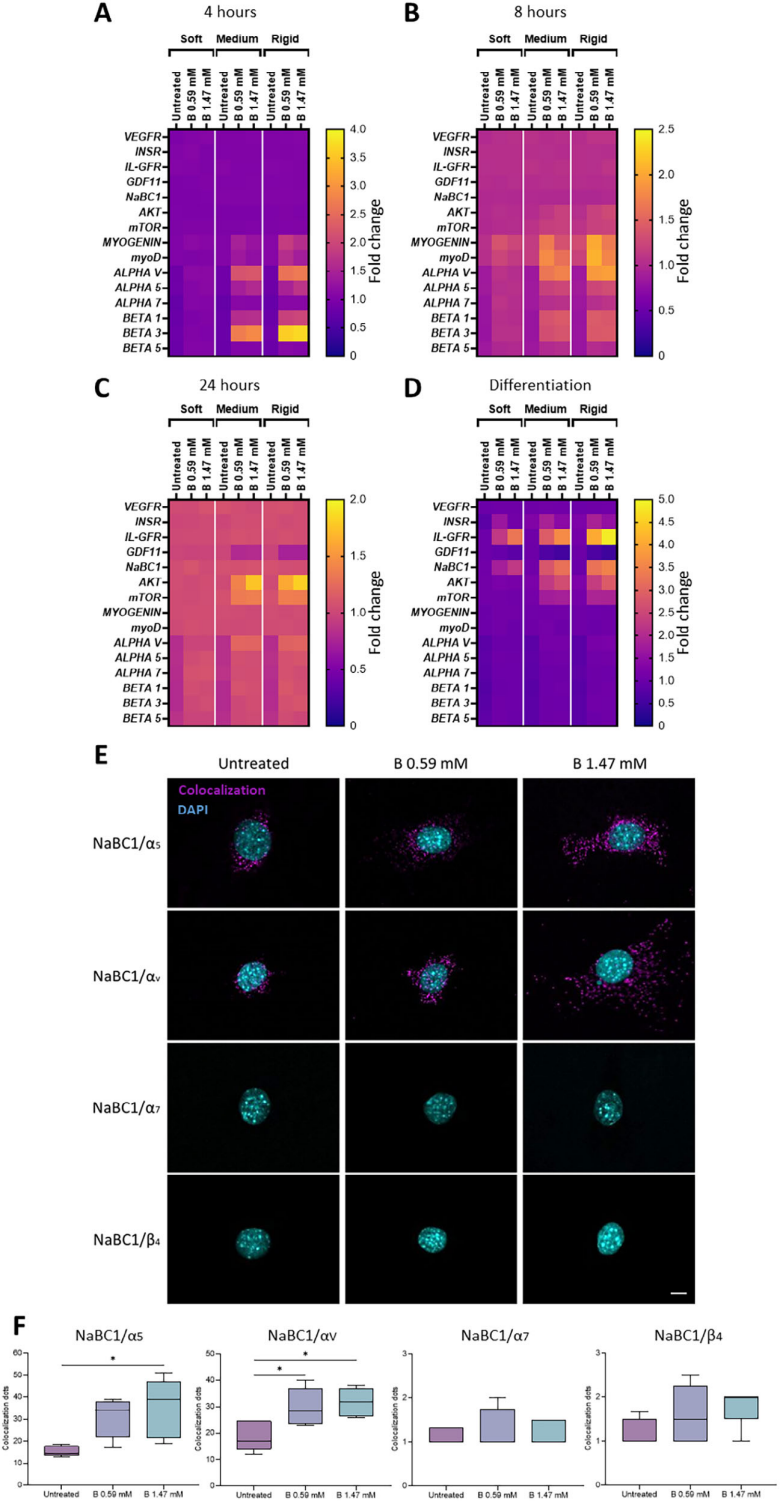
NaBC1 controls intracellular signaling via cooperation with fibronectin‐binding integrins. A–C) Heat maps of gene expression of boron transporter (*NaBC1*), myogenesis markers (*MYOD, MYOGENIN*), AKT/mTOR pathway (*AKT, mTOR*), muscle metabolism (*IL‐GFR*, *INSR*, *GDF11*, *VEGFR*), cell adhesion‐related genes (*ALPHAV*, *ALPHA5*, *ALPHA7*, *BETA1*, *BETA3*, *BETA5* Integrins) in C2C12 myoblasts seeded on PAAm hydrogels of different stiffnesses, functionalized with fibronectin (FN) and stimulated with soluble boron (B) (at 0.59 and 1.47 mm) for A) 4, B) 8, or C) 24 h compared to untreated cells on cell culture plates, as measured by qPCR. D) Heat map of gene expression in C2C12 myoblasts seeded on PAAm hydrogels of different stiffnesses, functionalized with FN, and stimulated with soluble B (at 0.59 and 1.47 mm) for 96 h in myogenic differentiation conditions, as measured by qPCR. For panels A–D, *n* = 3 biological replicates with three technical replicates. E) Colocalization assays were performed by using the Duolink PLA protein detection technology, which is based on in situ proximity ligation assay (PLA) that allows the visualization and quantification of protein–protein interactions when proteins are present within 40 nm. Representative images showing the colocalization dots of NaBC1/α_5_, NaBC1/α_v_, NaBC1/α_7_, and NaBC1/β_4_ in C2C12 myoblasts seeded on rigid PAAm hydrogels, functionalized with FN for 1 h, and stimulated with soluble B (0.59 and 1.47 mm). Magenta: colocalization dots; Cyan: DAPI. Scale bar: 50 µm. F) Quantification of a number of colocalization dots of NaBC1/α_5_, NaBC1/α_v_, NaBC1/α_7_, and NaBC1/β_4_. *n* = 30 cells from three different biological replicates. Data are represented as Mean ± Standard Deviation, and differences are considered significant for *p* ≤ 0.05 using one‐way ANOVA (Tukey's multiple comparisons tests) for multiple comparisons. *p ≤ 0.05.

To decipher the role of NaBC1 in the molecular clutch, we investigated the PI3K/AKT signaling pathway as previous studies have highlighted that this pathway undergoes adhesion‐dependent activation.^[^ ^48^ ^]^ **Figure** **3**A–D shows that *AKT* and *mTOR* gene expression levels were upregulated up to four‐fold in C2C12 myoblasts seeded on medium and rigid substrates (at 24 h and 96 h) following NaBC1 stimulation, compared to non‐stimulated myoblasts. We have previously demonstrated that B‐loaded hydrogels promote muscle regeneration in vitro and in vivo.^[^ ^29^ ^]^ We, therefore, assayed the expression of the Vascular Endothelial Growth Factor receptor (*VEGFR*), Insulin receptor (*INSR*), and insulin‐like growth factor receptor (*IL‐GFR*) genes, which are important for the functions of muscle cells.^[^ ^49^, ^50^, ^51^ ^]^ Our results showed that substrate rigidity had no influence on the expression of these genes. However, NaBC1 stimulation with B at 1.47 mm boosted *IL‐GFR* gene expression up to five times on all substrates. Myogenin and myoD are typical markers of early myogenic differentiation.^[^ ^52^, ^53^ ^]^ Figure 3 A‐D shows that the expression of both markers was upregulated 4 h after C2C12 myoblasts were seeded on medium substrates (*E =*9 kPa). These substrates have a similar level of elasticity to that of healthy human skeletal muscles, such as the *flexor digitorum profundus* (*E =*8.7 kPa) and the *gastrocnemius* (*E =*9.9 kPa).^[^ ^54^ ^]^ In contrast to IL‐GFR, which promotes muscle growth, growth differentiation factor 11 (GDF11) inhibits myogenesis via the phosphorylation of SMAD2/3 transcription factors.^[^ ^55^ ^]^ Our results show, for the first time, that NaBC1 stimulation downregulated *GDF11* expression in C2C12 myoblasts seeded on medium and rigid substrates after 24 h, demonstrating a relationship between NaBC1 and GDF11 conditioned by the rigidity of the substrate.

Cells perceive force through a variety of molecular sensors, of which ion channels and transporters are the fastest and most efficient. Several studies have previously reported that ion‐channels and integrins,^[^ ^56^ ^]^ as well as ion‐transporters and integrins,^[^ ^57^ ^]^ can physically couple together to produce a cluster at the cell membrane. These clusters activate integrin‐channel crosstalk and induce reciprocal signaling in which cell adhesion can induce channel activation^[^ ^58^ ^]^ and channel engagement can regulate cell adhesion.^[^ ^59^, ^60^ ^]^ Integrins might play a role in the localization of ion channels/transporters in the plasma membrane and ion channel regulation via the formation of macromolecular complexes that further regulate downstream signaling proteins. Indeed, ion channels sometimes transmit their signals through conformational coupling.^[^ ^61^ ^]^ The channel protein is not merely a final target because it often feeds back by controlling integrin activation and/or expression, as occurs with different ion channels that couple with integrin β_1_ and activate its expression.^[^ ^62^, ^63^ ^]^ Here, we report that a combination of NaBC1 stimulation and substrate rigidity upregulates the expression of genes that encode fibronectin‐binding integrins, such as α_v_, α_5_, β_1_ and β_3,_ in C2C12 myoblasts at 4 h and, to a lesser extent, at 8 h after NaBC1 stimulation (**Figures 3A,B**; Figure S5, Supporting Information). By contrast, the expression of α_7_ and β_5_ remained unaltered in C2C12 cells seeded on medium or rigid substrates. When C2C12 myoblasts were seeded on soft hydrogels, NaBC1 stimulation did not alter the downregulation of integrin expression (α_v_, α_5_, α_7_, β_1_, β_3_, and β_5_) (Figures 3 A‐C; Figure S5,Supporting Information), as is typical of substrates of low elastic modulus.^[^ ^64^ ^]^ To investigate whether integrin upregulation after NaBC1 stimulation occurred as a consequence of the interaction between α_5_β_1_ and α_v_β_3_ integrins and NaBC1, we measured the colocalization of NaBC1/α_5_‐α_v_ using the DUOLINK PLA kit system (Figure 3E,F). Each dot in Figure 3E corresponds to the signal generated by two different proteins that are closer than 40 nm. Our results show that the addition of B to C2C12 myoblasts seeded on rigid substrates led to the colocalization of fibronectin‐binding integrins (α_5_ and α_v_) and NaCB1 in a dose‐dependent manner (Figure 3F). We did not observe any effect of B stimulation on the colocalization of NaBC1 and other integrin receptors, such as α_7_ and β_4_ integrins (specific laminin receptors) (Figure 3E,F).^[^ ^65^, ^66^ ^]^ Together, these results demonstrate that NaBC1 cooperates with fibronectin‐binding integrins in response to substrate rigidity. We therefore conclude that NaBC1 stimulation, in substrates of high enough elasticity, induces the expression of genes that encode fibronectin‐binding integrins and components of the cell adhesion signaling pathways, AKT‐mTOR.

**Figure 4:**
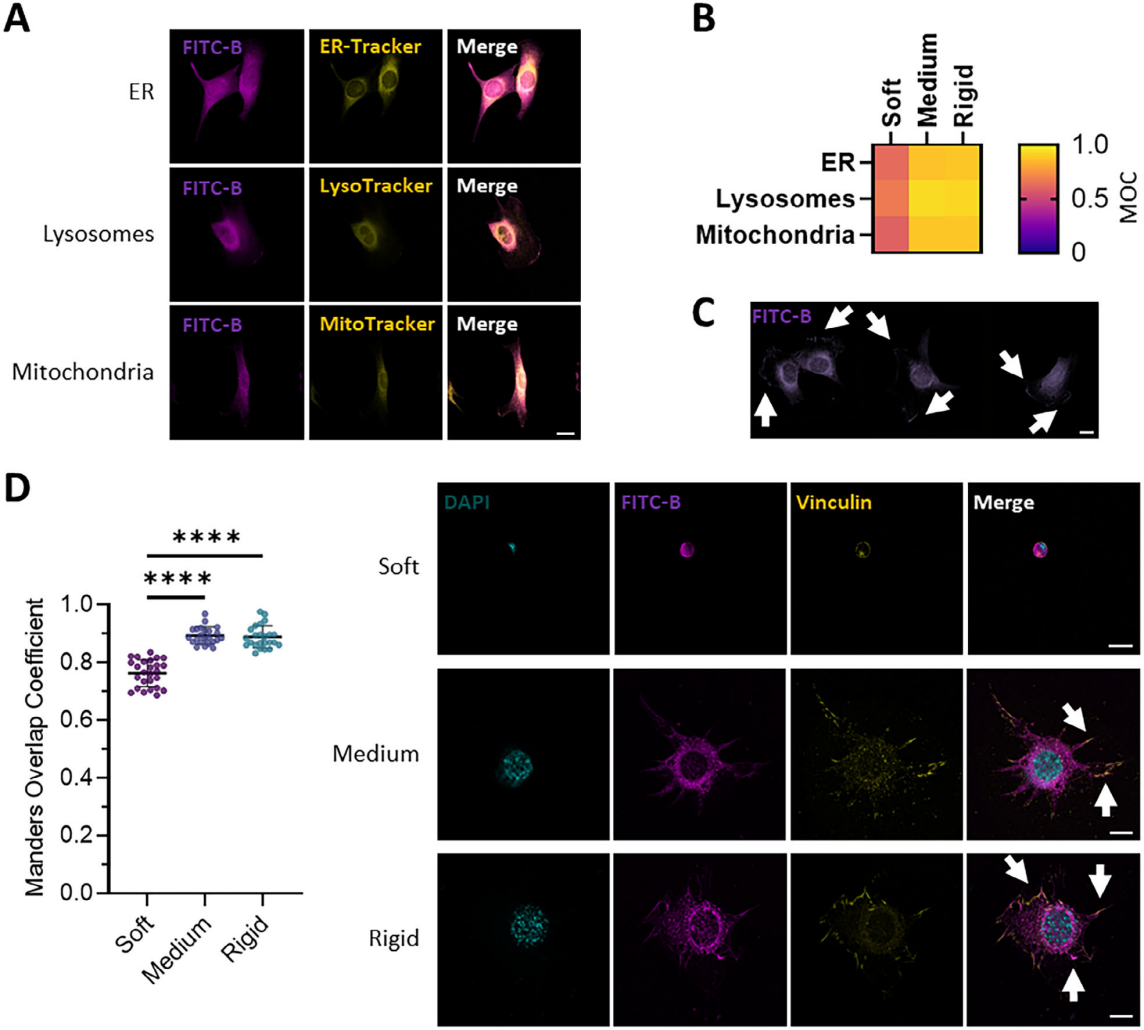
Boron subcellular localization highlights its presence in focal adhesions. A) Representative images of the colocalization of FITC‐labelled Boron (FITC‐B) and live trackers in C2C12 myoblasts seeded on rigid PAAm hydrogels functionalized with fibronectin (FN). Magenta: FITC‐B. Yellow: live trackers for the ER (ER‐Tracker), lysosomes (LysoTracker), and mitochondria (MitoTracker). Scale bar: 20 µm. B) Heat map of B and live tracker colocalization in C2C12 myoblasts seeded on rigid PAAm hydrogels functionalized with FN. C) Representative images of the subcellular localization of FITC‐B in C2C12 myoblasts seeded on rigid PAAm hydrogels functionalized with FN. Magenta: FITC‐B. Arrows indicate clumps in cell edges. Scale bar: 20 µm. D) (Left) Quantification of FITC‐B and focal adhesion colocalization in C2C12 cells seeded on hydrogels of different stiffnesses, as scored using the Manders Overlap Coefficient (MOC). (Right) Representative images of FITC‐B and focal adhesion colocalization in C2C12 myoblasts seeded on PAAm hydrogels of different stiffnesses functionalized with FN. Magenta: FITC‐B; Yellow: vinculin; Cyan: DAPI. Scale bars: 20 µm. *n* = 20 cells from three different biological replicates. Data are represented as Mean ± Standard Deviation, and differences are considered significant for *p* ≤ 0.05 using one‐way ANOVA (Tukey's multiple comparisons tests) for multiple comparisons. ****p ≤ 0.0001.

### 2.3 Intracellular Dynamics of B

To test the intracellular dynamics of B, C2C12 myoblasts were incubated with FITC‐labelled boron (FITC‐B) in combination with multiple live trackers for specific cell compartments (the endoplasmic reticulum (ER), lysosomes, and mitochondria). Figure 4A,B shows that FITC‐B colocalizes with lysosomes, mitochondria, and the ER in C2C12 myoblasts seeded on medium and rigid hydrogels but not on soft ones. We also observed FITC‐B forming clumps at the cells’ edges (Figure 4C). We, therefore, hypothesized that NaBC1 might be present in focal adhesions since we had previously observed its colocalization with integrins. To test this hypothesis, we investigated the colocalization of FITC‐B and vinculin (Figure 4D). The Manders Overlap Coefficient (MOC) quantifies the amount of fluorescence overlapping between two channels ranging from 0 (an “anti‐colocalization”) to 1 (a perfect colocalization) and is widely used as a quantitative tool to evaluate colocalization in biological microscopy.^[^ ^67^ ^]^ MOC was low for myoblasts seeded on soft hydrogels but was close to 1 for those seeded on medium and rigid hydrogels. This result confirms the presence of NaBC1 in focal adhesions in cells seeded on medium and rigid substrates and corroborates the cooperation and colocalization between NaBC1 and fibronectin‐binding integrins, triggered by the elasticity of the substrate.

FRAP (Fluorescence Recovery After Photobleaching) is a direct, noninvasive method used to study the mobility of biological molecules in living cells.^[^ ^68^ ^]^ FRAP corroborated our earlier finding that FITC‐B colocalizes with focal adhesions, as demonstrated by the results we obtained with the living trackers (Figure S7, Supporting Information). Our FRAP results also showed that there were lower levels of FITC‐B in the cell nuclei compared to the cytoplasm (Figure S7A, Supporting Information) and that FITC‐B undergoes continuous influx into and efflux out of mitochondria and lysosomes (Figure S7A, Supporting Information). Furthermore, the short half‐life and decreased fluorescence signal of FITC‐B over time in focal adhesions indicate that FITC‐B is internalized through these structures, supporting the colocalization of NaBC1 and fibronectin‐binding integrins (Figure S7B, Supporting Information).

Given that FITC‐B colocalizes with mitochondria in C2C12 cells seeded on rigid substrates, we hypothesized that B might have a role in mitochondrial metabolism. Although previous studies have indicated that a relationship exists between cell mechanics and metabolism,^[^ ^69^ ^]^ how the mechanical cues exerted by the ECM influence metabolic pathways remains poorly understood.^[^ ^70^, ^71^ ^]^ It is known that mechanotransduction pathways control cell processes (such as proliferation, differentiation, and death) that require energy generation and the biosynthesis of macromolecules.^[^ ^72^ ^]^ Indeed, ≈50% of the ATP consumed by platelets^[^ ^73^ ^]^ and neurons^[^ ^74^ ^]^ is required to support the polymerization and rearrangement of the actin cytoskeleton. To test the effect of substrate elasticity and NaBC1 on cell metabolism, we measured total ATP and mitochondrial ATP (mATP) content in C2C12 myoblasts (Figure S8, Supporting Information). We detected the same level of total ATP for both medium and rigid substrates (Figure S8A, Supporting Information). When C2C12 myoblasts were seeded on rigid hydrogels, NaBC1 stimulation further increased their ATP content, relative to cells not treated with B on the same substrate, supporting a role for NaBC1 in mechanotransduction in the actin cytoskeleton of cells under higher tension (Figure S8B, Supporting Information).^[^ ^70^, ^75^ ^]^

### 2.4 NaBC1 Interacts with β_1_ Integrin and Vinculin on Rigid Hydrogels, and This Interaction is Amplified in the Presence of B

**Figure 5:**
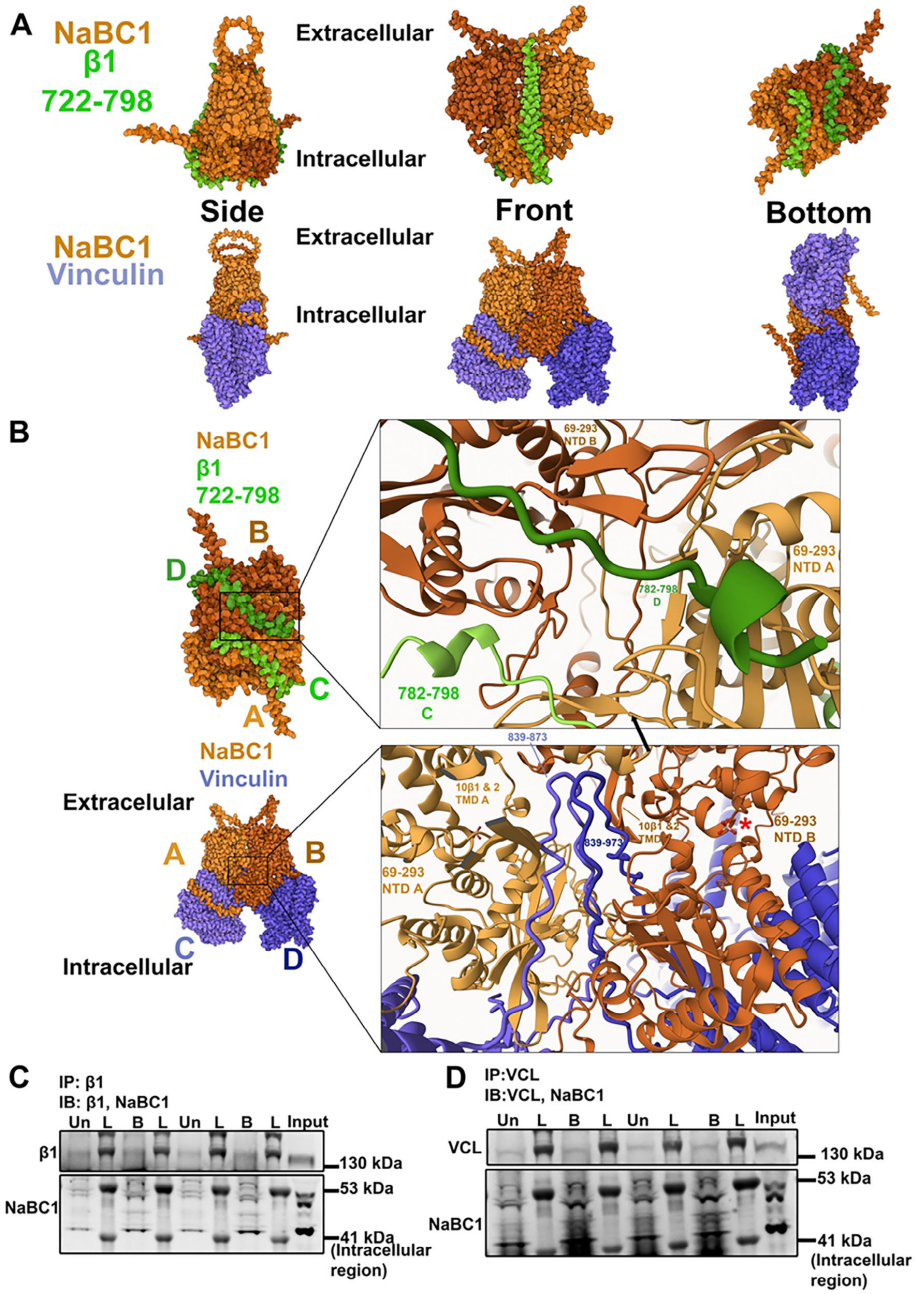
AlphaFold 3 predictions and experimental validation of NaBC1 interaction with β_1_ integrin and vinculin. A) AlphaFold 3 models of the interaction between a NaBC1 dimer (both monomers shown in orange, with monomer A slightly lighter than monomer B for clarity) and the transmembrane domain along with the intracellular C‐terminal tail of β_1_ integrin (residues 722–798, both monomers shown in green, with monomer C lighter than monomer D for clarity). The models, viewed from the side, front, and bottom (intracellular side), predict the transmembrane helix of β_1_ integrin to sit in a groove on the side of the interacting face of the NaBC1 dimer. The C‐terminal tail of β_1_ integrin is predicted to wrap along the underside of the intracellular domain of NaBC1. The model of NaBC1 and vinculin (both monomers shown in purple, with monomer C lighter than monomer D for clarity) suggests that vinculin interacts with both the transmembrane domain (TMD) and the N‐terminal intracellular domain (NTD) of NaBC1. B) Detailed view of the interactions. The C‐terminal tail of each β_1_ integrin monomer is predicted to interact with the intracellular domains of both NaBC1 monomers. A section of vinculin's proline‐rich hinge region (residues 839–873) is predicted to insert between the NaBC1 monomers, reaching from the intracellular domains up to the transmembrane domains and interacting with the 10 β1 and 10 β2 β‐sheets of NaBC1. Red asterisk represents the presence of B ion. pLDDT confidence scores: 68.6 for NaBC1/β_1_ integrin; 76.1 for NaBC1/vinculin. C) Immunoblots validating the interaction of NaBC1 with β_1_ integrin. β_1_ integrin was immunoprecipitated from cells cultured on rigid hydrogels in the absence or presence of B (1.47 mm) for 24 h. Immunodetection was performed using antibodies specific for β_1_ integrin and NaBC1. An increase in the interaction of NaBC1 with β_1_ integrin was observed in the presence of B. D) Immunoblots showing interaction between NaBC1 and vinculin (VCL). Vinculin was immunoprecipitated using protein‐specific antibodies, and NaBC1 and vinculin were immunodetected. An increase in the interaction of NaBC1 with β_1_ integrin was observed in the presence of B. IP, Immunoprecipitated protein; IB, Immunodetected protein; Un, untreated cells; L, protein ladder; B, B‐treated cells. Cell lysate is referred to as input to confirm protein‐specific bands. All immunoblot experiments were duplicated with identical results. Uncropped immunoblots are presented in Figure S14 (Supporting Information).

AlphaFold 3 modeling and co‐immunoprecipitation were performed to study the interaction (**Figures** **5**; Figure S13–16, Supporting Information) between the Boron transporter NaBC1 and β_1_ integrin, (NaBC1/β_1_) and between NaBC1 and vinculin (NaBC1/vinculin). Initial AlphaFold 3 modeling of the NaBC1 dimer in complex with β1 integrin showed inconclusive models due to the presence of the extracellular domain interacting with the intracellular domain of NaBC1. To overcome this, predictions were repeated with NaBC1 dimers using two copies of only residues 722–798 of β_1_ integrin, encompassing the transmembrane domain and intracellular C‐terminal tail, revealing a distinct interaction interface (Figure 5A). The transmembrane domain helix of β_1_ integrin was predicted to sit within a groove along the interacting face between the two monomers of the NaBC1 dimer. Furthermore, the C‐terminal tail of β_1_ integrin was predicted to extend below and wrapped around the intracellular portion of the NaBC1 dimer, suggesting an interaction mediated by both transmembrane and intracellular domains (Figure 5B). The predicted interaction between the intracellular domains of NaBC1 and the C‐terminal tail of β_1_ integrin suggests a potential mechanism for allosteric regulation or signal transduction. Three out of the five β_1_ integrin models were effectively the same, with the other two showing the transmembrane helix s in a similar position as the other three (Figure S13, Supporting Information). Four out of the five vinculin models were very similar, with only one showing a different conformation (Figure S13, Supporting Information).

In a separate model, AlphaFold 3 predicted an interaction between NaBC1 and vinculin (Figure 5A), mediated by both the transmembrane and N‐terminal intracellular domains of NaBC1. Residues 839–873, within vinculin's proline‐rich hinge region, were predicted to be inserted between the two NaBC1 monomers (Figure 5B), extending from the intracellular domains toward the transmembrane domains of NaBC1, suggesting interaction with vinculin could potentially influence NaBC1 conformation or activity. Four out of the five vinculin models were very similar, with only one showing a different conformation (Figure S13, Supporting Information).

We then performed co‐immunoprecipitation to experimentally validate if NaBC1 interacts with β_1_ integrin or vinculin. C2C12 cells were seeded on rigid hydrogels in the absence or presence of B (1.47 mm) for 24 h. β_1_ integrin and vinculin were immunoprecipitated using specific antibodies, and we assessed if NaBC1 was pulled down along with β_1_ integrin or vinculin. We observed that both β_1_ integrin and vinculin interact with NaBC1 (Figure 5; the uncropped immunoblots are shown in Figure S14, Supporting Information). Furthermore, a higher amount of NaBC1 interacting with β_1_ integrin or vinculin was observed in the presence of Boron than without it (Figure 5). This confirmed that the presence of Boron increases interaction between NaBC1 and β_1_ integrin as well as between NaBC1 and vinculin. Interestingly, the vinculin antibody used detects vinculin residues 777–1066, which fit the residues predicted by AlphaFold 3, confirming that vinculin proline‐rich hinge interacts with NaBC1 (839‐873). We noted that we failed to observe the full‐length NaBC1 protein (98 kDa) and questioned if it cleaves into separate fragments during the extraction process. To validate this, crude cell lysate with NaBC1 antibody alone was first probed (Figure S15, Supporting Information). We observed protein bands at 41 and 52 kDa, which can be attributed to cleaved 41 kDa intracellular region, as also shown by Vilas et al.^[^ ^76^ ^]^ These protein bands were also observed when the immunocomplexes were silver‐stained (Figure S15, Supporting Information). We note that, although Vilas et al. confirmed the presence of a 56 ± 3 kDa transmembrane region (499 amino acid long) of NaBC1 in mammalian cells, however, the NaBC1 antibody used in immunoblotting (ABN1718, Merck) only detects NaBC1 at C‐terminus, making it impossible for the 52 kDa NaBC1 protein band to be the transmembrane region. To further validate if the antibody can detect full‐length NaBC1, NaBC1 was immunoprecipitated (instead of β_1_ integrin or vinculin, Figure S16, Supporting Information). We did not observe protein bands close to 98 kDa which that can be assigned to NaBC1 but instead observed bands close to 41 kDa, confirming that NaBC1 cleaves into several smaller fragments including a 41 kDa intracellular region which interacts with β_1_ integrin and vinculin as a function of B. Collectively, results confirm that NaBC1 interacts with both adhesion proteins β_1_ integrin and vinculin on rigid hydrogels and this interaction is amplified in the presence of B.

### 2.5 NaBC1 is Not Involved in Impaired Cell Response to Stiffness on Laminin‐111‐Coated Substrates

**Figure 6:**
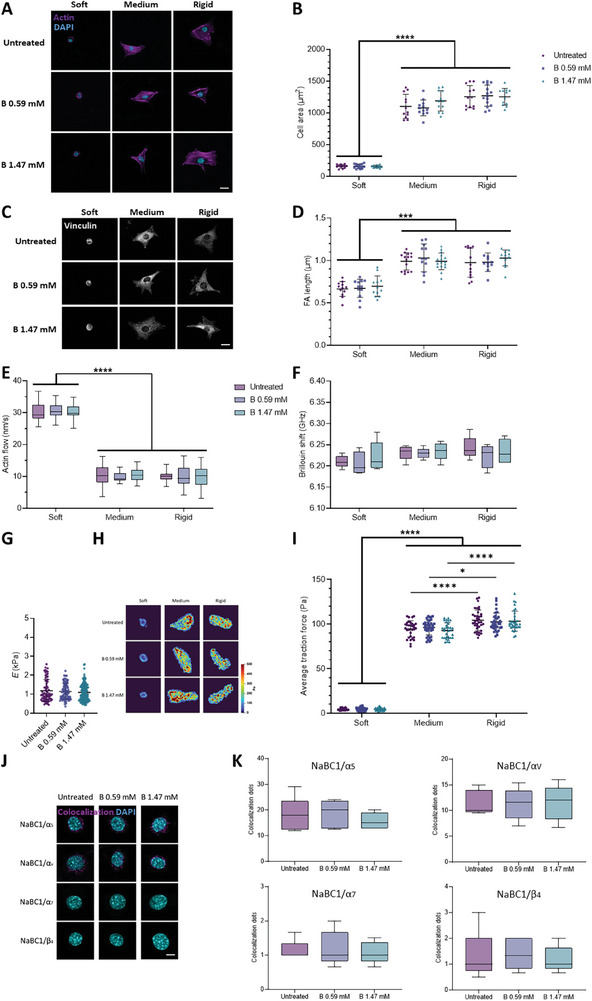
NaBC1 is not involved in the impaired cell response to stiffness on laminin‐111. The results reported in panels A–F and H–J derive from experiments in which C2C12 myoblasts were seeded on PAAm hydrogels of different stiffnesses (soft, medium, and rigid) that were functionalized with laminin‐111 and stimulated with soluble boron ions at two different concentrations (0.59 and 1.47 mm). A) Representative immunofluorescence images of C2C12 myoblasts cultured as described. Magenta: actin cytoskeleton; Cyan: DAPI. Scale bar: 20 µm. B) Quantification of cell area of C2C12 myoblasts cultured as described. *n* = 10 cells from three different biological replicates. C) Representative immunofluorescence images of C2C12 myoblasts cultured as described. Gray: vinculin. Scale bar: 20 µm. D) Quantification of focal adhesion (FA) length in C2C12 myoblasts cultured as described. *n* = 10 cells from three different biological replicates. E) Quantification of actin retrograde flow in C2C12 myoblasts cultured as described. *n* = 5 cells with at least five different flow areas per cell. F) Quantification of Brillouin shift in C2C12 myoblasts cultured as described and imaged with Brillouin microscopy. *n* = 10 cells from three different biological replicates. G) Quantification of cell stiffness by nanoindentation of C2C12 myoblasts seeded on glass coverslips functionalized with laminin‐111 and stimulated with soluble B, as described. *n* = 10 cells with nine indentations on each single cell from three different biological replicates. H) Representative traction maps of C2C12 myoblasts cultured as described. I) Quantification of traction forces exerted by C2C12 myoblasts when cultured as described. *n* = 30 cells from ten different locations within each hydrogel from three different biological replicates. J) Colocalization assays were performed by using the Duolink PLA protein detection technology, which is based on in situ proximity ligation assay (PLA) that allows the visualization and quantification of protein–protein interactions when proteins are present within 40 nm. Representative images of colocalization dots of NaBC1/α_5_, NaBC1/α_v_, NaBC1/α_7_ and NaBC1/β_4_ in C2C12 myoblasts cultured as described. Magenta: colocalization dots; Cyan: DAPI. Scale bar: 50 µm. K) Quantification of the number of colocalization dots of NaBC1/α_5_, NaBC1/α_v_, NaBC1/α_7_, and NaBC1/β_4_. *n* = 30 cells from three different biological replicates. Data are represented as Mean ± Standard Deviation, and differences are considered significant for *p* ≤ 0.05 using one‐way ANOVA or two‐way ANOVA (Tukey's multiple comparisons tests) for multiple comparisons. *p ≤ 0.05, ***p ≤ 0.001, ****p ≤ 0.0001.

To test the role of NaBC1 in cell mechano‐responses, we conducted further experiments on PAAm hydrogels coated with laminin‐111 (**Figure** **6**), as laminin‐111 has been recently shown to impair breast epithelial cell responses to substrate elasticity.^[^ ^77^ ^]^

We observed that when C2C12 cells were seeded onto soft PAAm hydrogels coated with laminin‐111, they remained small and did not spread (Figure 6A,B). Moreover, there were no differences regarding the cell area between C2C12 cells seeded on medium and rigid hydrogels coated with laminin‐111. This is in contrast to what we had previously observed, i.e., a continuous increase in cell area when cells were seeded on fibronectin‐coated hydrogels, as the rigidity of the substrate increased (Figure 1A,B). Importantly, in substrates coated with laminin‐111, NaBC1 stimulation with soluble B at different concentrations did not alter this rounded cell morphology, as reported earlier in Figure 1, and the cell adhesive response on rigid and medium substrates was similar between them.

When C2C12 myoblasts were seeded on soft hydrogels coated with laminin‐111, they kept showing low numbers and small FAs (Figure 6D; Figures S10,S26–S28, Supporting Information). These cells were also unable to respond to substrate stiffness, and differences in FA length and number between medium and rigid hydrogels were not observed (≈30 FA and 0.4 µm). Interestingly, B did not increase the length of FAs on these substrates, in contrast to what we had previously observed in cells seeded on fibronectin‐coated substrates (Figure 1E,F). We also assessed retrograde actin flow (Figure 6E) and observed the same trend: that the previously observed difference in retrograde actin flow in cells cultured on medium and stiff fibronectin‐coated substrates was lost when cells were seeded on medium and stiff laminin‐111 coated substrates. We also saw no retrograde actin flow response to the stimulation of NaCB1 with B in these cells (Figure 6E). Cell stiffness and cell traction forces exerted on laminin‐111‐functionalized substrates were measured using Brillouin microscopy/nanoindentation and traction force microscopy (Figure 6F–I). Undoubtedly, the weak adhesion of cells to laminin‐111‐coated substrates might explain the higher retrograde actin flow, lower cell stiffness, and lower forces exerted in comparison to cells on fibronectin‐coated substrates. We also performed PLA colocalization assays and observed no interactions between NaBC1 and fibronectin‐binding (α_5_‐α_v_) or laminin‐binding (α_7_‐β_4_) integrins on PAAm hydrogels of different stiffnesses that were functionalized with laminin‐111 (Figure 6J,K). The ATP content (both total and mATP) remained constant in cells on medium and rigid substrates even after incubation with soluble B (Figure S17, Supporting Information). The lack of response to the B‐mediated stimulation of NaCB1 on laminin‐coated substrates—which hinder mechanotransduction and do not engage the molecular clutch^[^ ^77^ ^]^—support the mechanosensitive nature of NaCB1.

As explained above, the combination of NaBC1 stimulation and substrate rigidity upregulates the expression of genes that encode fibronectin‐binding integrins, such as α_v_, α_5_, β_1_ and β_3,_ in C2C12 myoblasts at 4 h and, to a lesser extent, at 8 h after NaBC1 stimulation (Figures 4A,B; Figure S5, Supporting Information). These results suggest that NaBC1 cooperates with fibronectin‐binding integrins in response to substrate rigidity. To corroborate the role of NaBC1 in cell mechano‐responses, we determined the gene expression of multiple integrins (α_v_, α_5_, α_7_, β_1_, β_3_, and β_5_) in C2C12 myoblasts seeded on PAAm hydrogels coated with laminin‐111. Figure S12 (Supporting Information) shows that the expression of integrins remained unaltered in C2C12 cells seeded on medium or rigid substrates. Moreover, NaBC1 stimulation did not alter the downregulation of integrin expression (αv, α5, α7, β1, β3, and β5) when C2C12 myoblasts were seeded on soft hydrogels, as is typical of substrates of low elastic moduli.^[^ ^64^ ^]^

### 2.6 NaBC1‐Silencing Decreases Cell Mechanotransduction on Fibronectin‐Coated Surfaces

**Figure 7:**
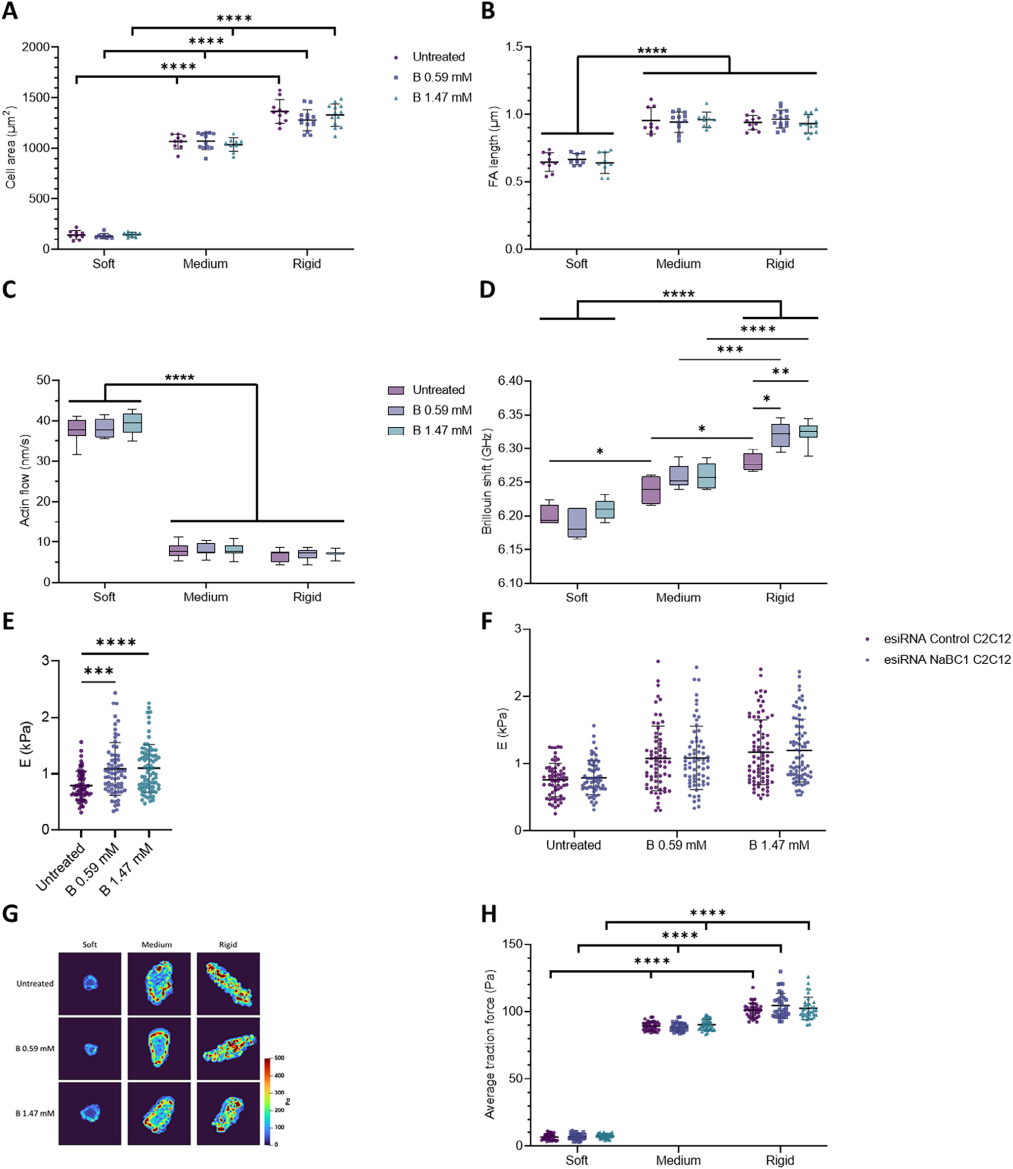
Cell mechanotransduction on fibronectin‐coated surfaces is dependent of NaBC1. The results reported in panels A–D derive from experiments in which NaBC1‐silenced C2C12 myoblasts were seeded on PAAm hydrogels of different stiffnesses (soft, medium, and rigid) that were functionalized with fibronectin and stimulated with soluble boron ions (B) at two different concentrations (0.59 and 1.47 mm). A) Quantification of cell area of NaBC1‐silenced C2C12 myoblasts that were treated and cultured as described. *n* = 10 cells from three different biological replicates. B) Quantification of focal adhesion (FA) length in NaBC1‐silenced C2C12 myoblasts that were treated and cultured as described. *n* = 10 cells from three different biological replicates. C) Quantification of actin retrograde flow in NaBC1‐silenced C2C12 myoblasts that were treated and cultured as described. *n* = 5 cells with at least five different flow areas per cell. D) Quantification of Brillouin shift in NaBC1‐silenced C2C12 myoblasts that were treated and cultured as described and imaged by Brillouin microscopy. *n* = 10 cells from three different biological replicates. E) Quantification of cell stiffness by nanoindentation of NaBC1‐silenced C2C12 myoblasts seeded on glass coverslips functionalized with FN and stimulated with soluble B (0.59 and 1.47 mm). *n* = 10 cells with nine indentations on each single cell from three different biological replicates. F) Comparison of cell stiffness by nanoindentation of NaBC1‐silenced and Control‐silenced C2C12 myoblasts seeded on glass coverslips functionalized with FN and stimulated with soluble B (0.59 and 1.47 mm). *n* = 10 cells with nine indentations on each single cell from three different biological replicates. G) Representative traction maps of NaBC1‐silenced C2C12 myoblasts that were treated and cultured as described. H) Quantification of traction forces exerted by NaBC1‐silenced C2C12 myoblasts that were treated and cultured as described. *n* = 30 cells from ten different locations within each hydrogel from three different biological replicates. Data are represented as Mean ± Standard Deviation, and differences are considered significant for *p* ≤ 0.05 using one‐way ANOVA or two‐way ANOVA (Tukey's multiple comparisons tests) for multiple comparisons. *p ≤ 0.05, **p ≤ 0.01, ***p ≤ 0.001, ****p ≤ 0.0001.

To demonstrate that the enhanced mechanotransductive effect of B on fibronectin‐coated substrates happens through NaBC1, we silenced NaCB1 using a NaCB1‐targeting esiRNA in C2C12 myoblasts (Figure S18, Supporting Information). esiRNA are comprised of a heterogeneous pool of siRNA all targeting the same mRNA sequence, thereby allowing us to perform post‐transcriptional silencing of NaBC1 in a highly specific and effective gene knockdown without off‐target effects. Figure S18B (Supporting Information) shows that esiRNA‐mediated silencing reduced NaBC1 expression to less than 10% of that in wild‐type myoblasts but did not affect cell viability (Figure S18C, Supporting Information). Furthermore, Figure S19 (Supporting Information) shows that cell transfection with esiRNA Control did not alter the cell response to substrate stiffness nor NaBC1 stimulation with soluble B. However, cell area and FAs were smaller and less numerous in NaCB1‐silenced cells, relative to wild‐type and transfection controls, indicative of lower cell spreading and decreased adhesion (**Figure** **7**A,B; Figures S21,S26–S28, Supporting Information). Following NaCB1 silencing, C2C12 cells were incubated with soluble B. The cell area and FA length of these cells remained the same as that of untreated cells, indicating that the NaBC1 receptor mediates the effects of B on the adhesiveness and morphology of these cells. We next measured retrograde actin flow in NaBC1‐silenced myoblasts, and observed no significant differences in retrograde actin flow between cells cultured on a medium or rigid substrate (Figure 7C). When NaBC1 was stimulated with soluble B in NaBC1‐silenced cells, it did not trigger any further decrease in actin flow. Unexpectedly, in NaBC1‐silenced cells after incubation with soluble B, we observed an increased Brillouin shift (up to 6.322 ± 0.018 GHz) (as measured by Brillouin microscopy, Figure 7D; Figure S22, Supporting Information) and Young´s modulus measured by nanoindentation (up to 1.21 ± 0.48 kPa) (Figure 7E). These increases were also noticeable in C2C12 myoblasts transfected with esiRNA Control (Figure S19, Supporting Information). However, untreated NaBC1‐silenced myoblasts presented similar Young´s modulus to Control‐silenced myoblasts and were 24% softer than untreated wild‐type myoblasts (Figure 7F), suggesting that the silencing process has an effect on cell membrane and stiffness. Since the silencing process inherently cannot achieve 100% efficiency, the increase in cell stiffness observed in NaBC1‐silenced cells following B stimulation may be attributed to the residual low levels of NaBC1 expression (<10% of the wild‐type levels) (Figure S18, Supporting Information). TFM measurements show that NaBC1‐silenced myoblasts exerted 20% lower forces compared to wild‐type cells, and that this was not increased by B stimulation (Figure 7G,H). These decreased traction forces were not shown by myoblasts transfected with esiRNA Control (Figure S19, Supporting Information), thereby discarding any effect of cell transfection. The total and mATP content also remained unaltered following the addition of B to NaBC1‐silenced cells (Figure S23, Supporting Information). Together, our results demonstrate that NaBC1 is responsible for enhanced mechanotransduction on fibronectin‐coated substrates after B stimulation of cells.

### 2.7 The Influence of NaBC1 on the Dynamics of the Actin Cytoskeleton is Linked to Talin–Vinculin Binding

**Figure 8:**
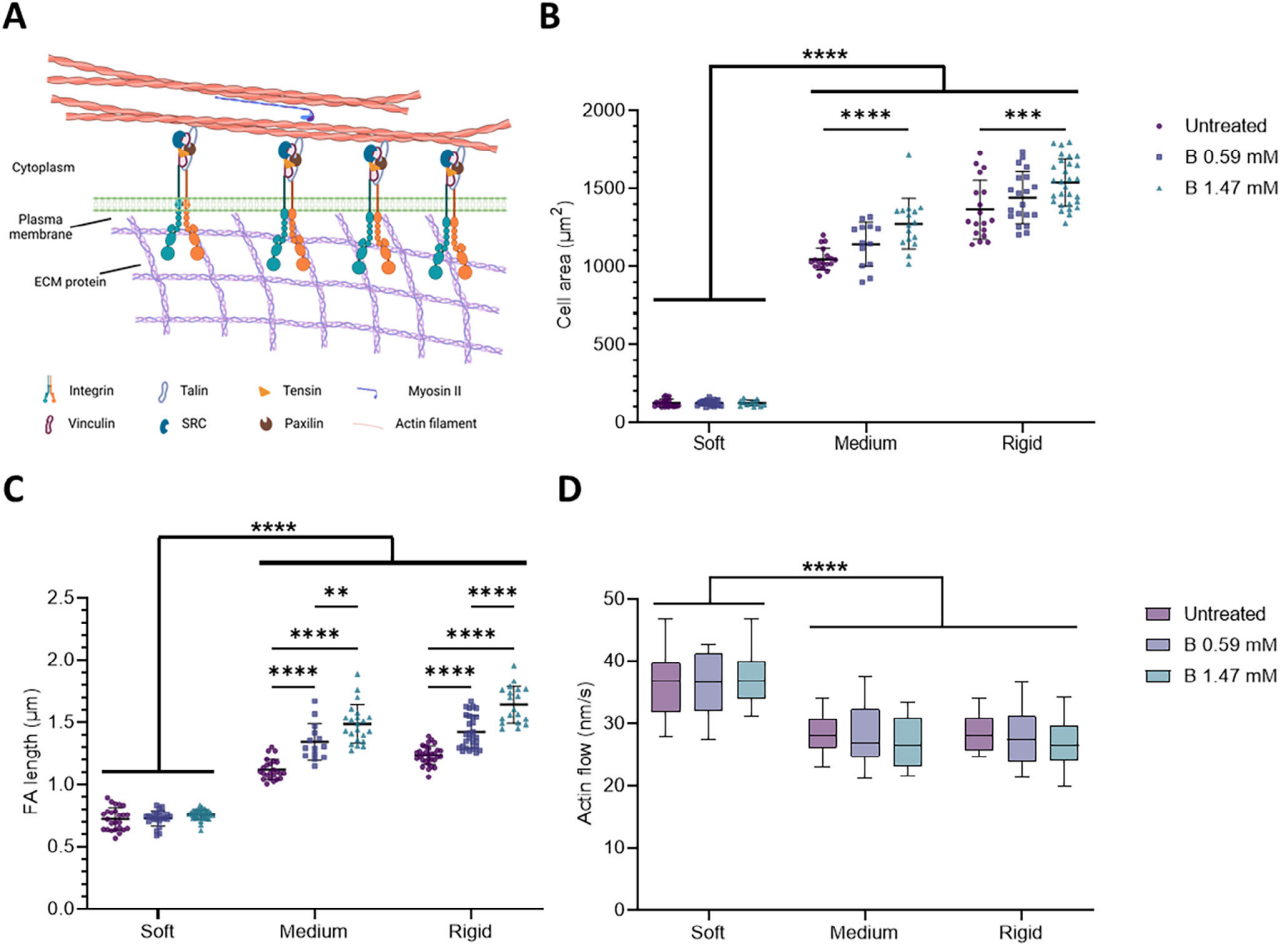
NaBC1 functions as a mechanosensor and is dependent of talin–vinculin binding. A) Schematic representation of the adhesion of a cell to ECM proteins via integrins. When talin unfolds in response to substrate rigidity vinculin can be recruited together with other proteins involved in the molecular clutch, forming mature focal adhesions and establishing the actin cytoskeleton. Schematic created with BioRender.com. Transfection with the VD1 plasmid, which encodes the vinculin head domain that can dominantly bind talin over endogenous vinculin, prevents the link between integrins and the actin cytoskeleton from being formed. Thus, VD1 mutant prevents cells’ response to the dynamics of the actin cytoskeleton that is mediated by talin unfolding. B) Quantification of cell area of C2C12 myoblasts transfected with VD1 seeded on PAAm hydrogels of different stiffnesses, functionalized with fibronectin (FN) and stimulated with soluble boron (B) (0.59 and 1.47 mm). *n* = 10 cells from three different biological replicates. C) Quantification of focal adhesion (FA) length in C2C12 myoblasts treated and cultured as described in panel B. *n* = 10 cells from three different biological replicates. D) Quantification of actin retrograde flow in C2C12 myoblasts treated and cultured as described in panel B. *n* = 5 cells with at least five different flow areas per cell. Data are represented as Mean ± Standard Deviation, and differences are considered significant for *p* ≤ 0.05 using two‐way ANOVA (Tukey's multiple comparisons tests) for multiple comparisons. ***p ≤ 0.001, ****p ≤ 0.0001.

We performed further experiments to investigate the crosstalk between the NaBC1 receptor and the molecular clutch. To do so, we transfected C2C12 myoblasts with the VD1 plasmid (**Figure** **8**; Figure S24, Supporting Information), which encodes a dominant protein composed of the head domain of vinculin that out competes endogenous vinculin for talin binding.^[^ ^6^, ^10^, ^78^ ^]^ Thus, VD1‐transfected cells break the link between integrins and the actin cytoskeleton, preventing the cells’ response to stiffness, which is mediated by talin unfolding.^[^ ^79^ ^]^ Figure 8B shows that the cell area of VD1‐transfected cells remains different on hydrogels of increasing stiffness (1047.16 ± 70.62 and 1364.78 ± 188.79 µm^2^ on medium and rigid hydrogels, respectively). When NaBC1 was activated in VD1 cells with different concentrations of B, cell area increased (Figure 8B), reaching values that are higher than those observed in wild‐type myoblasts (Figure 1D, 1274.40 ± 162.50 and 1539.66 ± 151.31 µm^2^ on medium and rigid hydrogels after stimulation with B 1.47 mm, respectively).

The transfection of C2C12 myoblasts with VD1 did not alter the formation of FAs, neither their length (Figure 8C) nor number (Figure S25, Supporting Information) compared to wild‐type cells. However, on stimulation of these cells with B, the number and size of their FAs increased (Figures 8C; Figures S26–S28, Supporting Information). VD1 impairs the link between integrins and the actin cytoskeleton and, as expected, transfected myoblasts with VD1 presented with high actin flow on medium (28.2 ± 2.8 nm s^−1^) and rigid (28.4 ± 2.8 nm s^−1^) hydrogels (Figure 8D), close to the actin flow values recorded for cells on soft substrates and significantly different from those of wild‐type myoblasts on hydrogels coated with fibronectin. The incubation of VD1 transfected cells with soluble B did not change their actin flow rates significantly, suggesting that the effect of NaBC1 on the dynamics of the actin cytoskeleton are dependent on proper talin folding/unfolding. We, therefore, conclude that our results and the molecular clutch model explain the active role of the NaBC1 transporter as a mechanosensor in response to stiffness.

### 2.8 NaBC1 Stimulation Induces Myogenic Differentiation in Medium and Rigid Substrates

**Figure 9:**
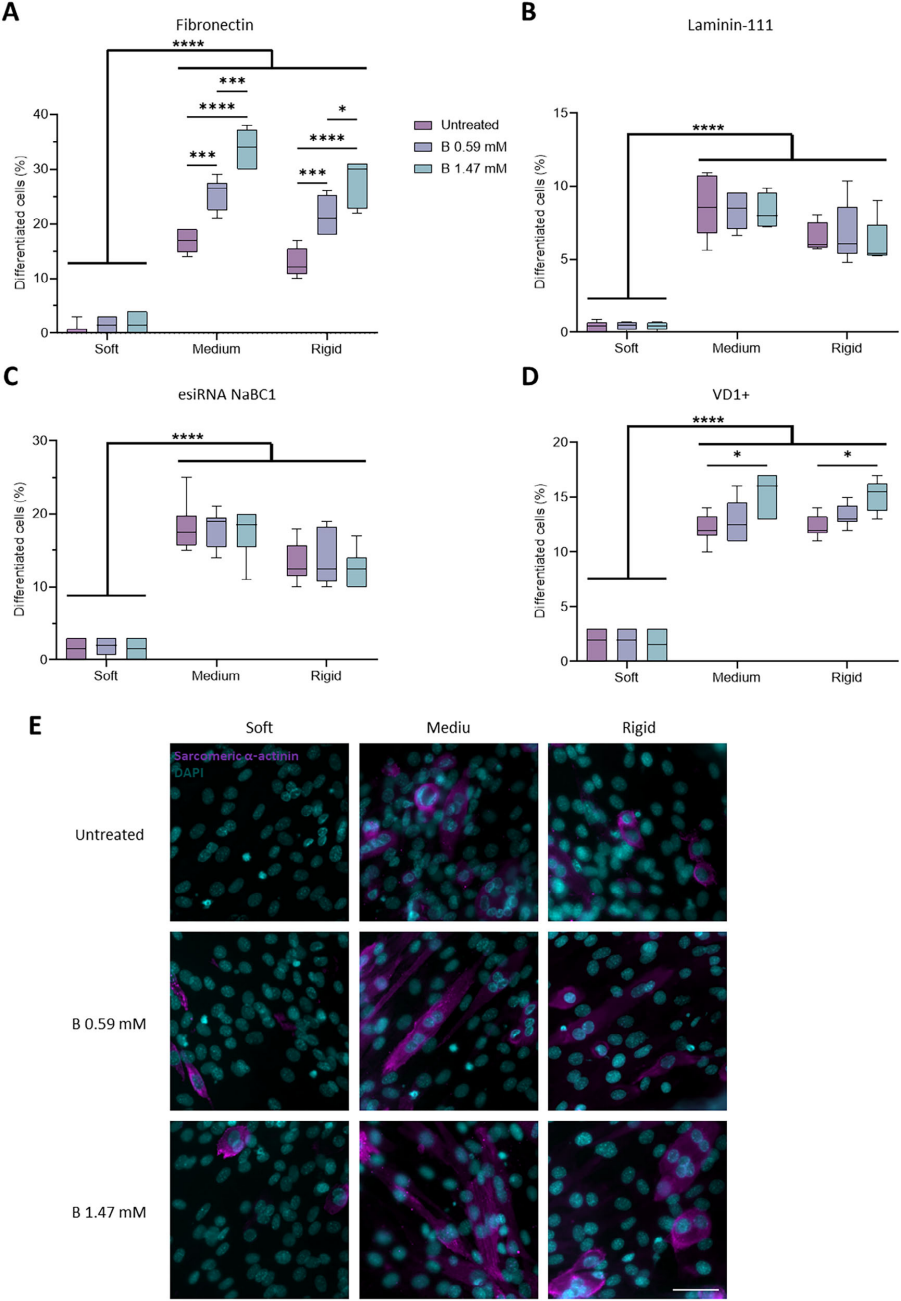
NaBC1 induces myogenic differentiation on fibronectin‐coated medium and stiff substrates. A) Quantification of myogenic differentiation in C2C12 myoblasts seeded on PAAm hydrogels of different stiffnesses, functionalized with fibronectin (FN) and stimulated with soluble boron (B) (0.59 and 1.47 mm). *n* = 10 images from three different biological replicates. B) Quantification of myogenic differentiation in C2C12 myoblasts seeded on PAAm hydrogels of different stiffnesses, functionalized with laminin‐111 and stimulated with soluble B ions (0.59 and 1.47 mm). *n* = 10 images from three different biological replicates. C) Quantification of myogenic differentiation in NaBC1‐KO C2C12 myoblasts, cultured as described in A. *n* = 10 images from three different biological replicates. D) Quantification of myogenic differentiation in C2C12 myoblasts transfected with the VD1 plasmid and cultured as described in A. *n* = 10 images from three different biological replicates. E) Representative images of myogenic differentiation in C2C12 myoblasts, cultured as described in A. Magenta: sarcomeric α‐actinin; Cyan: DAPI. Scale bar: 100 µm. Myotubes were counted when three or more cell nuclei were aligned. Data are represented as Mean ± Standard Deviation, and differences are considered significant for *p* ≤ 0.05 using two‐way ANOVA (Tukey's multiple comparisons tests) for multiple comparisons. *p ≤ 0.05, ***p ≤ 0.001, ****p ≤ 0.0001.

To understand the biological importance of the NaBC1 B transporter, we studied the formation of myotubes in vitro by determining the expression of sarcomeric α‐actinin, a typical marker for myogenesis.^[^ ^80^ ^]^ The fusion of myoblasts into myotubes is a phase of skeletal myogenesis that is essential for muscle repair.^[^ ^81^, ^82^ ^]^ After cell adhesion, when cultured in low serum conditions, myoblasts spread, elongate, and fuse into myotubes.^[^ ^83^, ^84^ ^]^ **Figure** **9** shows C2C12 myotubes after 4 days of culture under differentiation conditions (no serum and 1% insulin‐transferrin‐selenium (ITS)). When C2C12 myoblasts were cultured on fibronectin‐coated hydrogels, NaBC1 stimulation with soluble B significantly induced myogenic differentiation on both medium and rigid substrates in a dose‐dependent manner (25.5 ± 2.9% and 33.8 ± 3.9% on medium hydrogels versus 21.5 ± 3.6% and 27.8 ± 4.2% on rigid hydrogels after incubation with 0.59 and 1.47 mm, respectively). Interestingly, the highest level of myogenic differentiation was achieved on medium hydrogels (*E =*9 kPa), which have a stiffness similar to that of healthy human skeletal muscles, such as the *flexor digitorum profundus* (*E =*8.7 kPa) and the *gastrocnemius* (*E =*9.9 kPa).^[^ ^54^ ^]^ The enhanced myogenic differentiation mediated by B stimulation of NaBC1 was not observed in C2C12 myotubes cultured on laminin‐111‐coated hydrogels, which demonstrates the importance of mechanotranduction in NaCB1‐treated cells which does not occur on cells cultured on laminin‐111 coated hydrogels (Figure 6). Importantly, the stimulation of esiRNA‐silenced NaBC1 cells with B also did not increase the percentage of differentiated cells. Finally, in C2C12 myoblasts transfected with the VD1 plasmid, we observed a dose‐dependent percentage of differentiation after NaBC1 stimulation (up to 15.3%) but markedly lower compared to wild‐type myoblasts (up to 33.8%). Together, our results show that the stimulation of NaBC1 with B in C2C12 myotubes cultured on medium or rigid substrates induces myogenic differentiation through a mechanism that involves cooperation with the molecular clutch through fibronectin‐binding integrins.

## 3 Conclusion

We show that the NaBC1 transporter is actively involved in regulating cell behavior and fate beyond its role in B homeostasis. We demonstrated that the response of myoblasts to substrates stiffness which has been described by the molecular clutch model is altered by stimulation of the NaBC1 boron transporter. NaBC1 plays an important role in cell mechanotransduction and contributes to cell response to substrate rigidity in coordination with fibronectin‐binding integrins (α_v_, α_5_, β_1_, and β_3_). Importantly, we show the absence of a response to substrate stiffness in NaBC1‐silenced cells. Further, that NaBC1 stimulation had no effect on cell adhesion in myoblasts cultured on laminin‐111‐coated substrates, on which the cellular response to substrate rigidity is impaired and independent of the actin–talin–integrin–fibronectin molecular clutch.^[^ ^77^ ^]^

## 4 Experimental Section

### Preparation of PAAm Hydrogels

For PAAm hydrogels, all reagents were acquired from Sigma–Aldrich. Briefly, 1 mL volumes were prepared using stock solutions of 40% acrylamide (AAm) and 2% N,N'‐methylenebisacrylamide (BisAAm) mixed in different ratios for specific hydrogel stiffnesses (**Table** **1**). Solution volumes were then made up to 400 µL with milli‐Q water, 25 µL 1.5% (w/w) tetramethylethylenediamine (TEMED) and 8 µL 5% (w/w) ammonium persulfate (APS) and mixed thoroughly. 10 µL of solution was spotted onto hydrophobic glass slides before placing acrylsilanized glass coverslips onto the spots. Gelation was allowed to occur at room temperature for 30 min before detaching and swelling in milli‐Q water overnight at 4 °C. For imaging of live samples, PAAm gels were prepared onto glass bottom dishes (ThermoFisher Scientific).

**Table 1:** Component ratios for the preparation of PAAm hydrogels with different stiffness.

Hydrogel	AAm		BisAAm	
	Percentage [%]	Volume [µL]	Percentage [%]	Volume [µL]
Soft	3	30	0.06	50
Medium	5	12	0.3	60
Rigid	10	325	0.3	257

### ECM Protein‐Functionalization of PAAm Hydrogels

PAAm hydrogels prepared on coverslips were transferred to multi‐well plates before covering with 0.2 mg mL^−1^ sulfosuccinimidyl 6‐(4′‐azido‐2′‐nitrophenylamino)hexanoate (sulfo‐SANPAH) (Thermo Fisher). Samples were placed in a 365 nm UV light source at a distance of ≈3 inches for 10 min. This step was repeated three times. Hydrogels were then washed with 50 mm HEPES buffer (pH 8.5) three times before coating with 10 µg mL^−1^ ECM protein (fibronectin or laminin‐111, as indicated) (Biolamina) in HEPES buffer and overnight incubation at 37 °C. Hydrogels were washed with milli‐Q water to remove excess protein.

### PAAm Hydrogels Functionalization Quantification

Hydrogels functionalization was quantified by measurement of fibronectin or laminin‐111 coating intensity. Hydrogels were washed with DPBS, blocked in 2% BSA in DPBS for 1 h at room temperature, and then incubated with mouse monoclonal primary antibody against fibronectin (1:400, Sigma–Aldrich) or laminin‐111 (1:100, Invitrogen) in blocking solution overnight at 4 °C. Hydrogels were then rinsed twice in DPBS/0.1% Triton X‐100 and incubated with goat anti‐rabbit Cy3‐conjugated (1:200, Jackson Immunoresearch) secondary antibody at room temperature for 1 h. Samples were imaged using a Zeiss Observer Z1 epifluorescence inverted microscope. Three replicates per sample were measured.

### PAAm Hydrogels Nanoindentation

Nanoindentation measurements were performed using a fiber–optic based nanoindentation device Chiaro (Optics11) mounted on top of an inverted optical microscope Zeiss Axiovert 200M (Zeiss). Measurements were performed following the standardized protocol described by Ciccone et al.^[^ ^85^ ^]^ using a cantilever with stiffness (k) 0.51 Nm^−1^ holding spherical tips of radius (R) 3 µm for medium and stiff gels, and 0.028 Nm^−1^ with 28.5 µm for soft gels, respectively. For each experimental condition, at least three hydrogels were indented with a minimum of 100 indentations per condition. For each indentation, the probe moved at a constant speed of 2 µm s^−1^ over a vertical range of 10 µm (displacement control). The forward segment of the collected force–displacement (F–z) curves was analyzed using custom open‐source software.^[^ ^85^ ^]^ Curves were first filtered using a Savitzky–Golay filter from the SciPy computing stack^[^ ^86^ ^]^ with window length of 25 nm and polynomial order of 3 to remove random noise. After the point where the probe came into contact with the cell (*z*_0_,*F*_0_) was identified with a thresholding algorithm to convert (F–z) curves into force–indentation (F–δ) curves. To quantify the elastic properties of the gels (Young's Modulus E), F–δ curves were fitted with the Hertz model (Equation 1) up to a maximum indentation of δ  =  0.1 *R*. The Poisson's ratio (ν) was taken as 0.5, assuming the material's incompressibility.

(1) $$F=\frac{4}{3}\frac{E}{\left(1-v^{2}\right)}\delta^{\frac{3}{2}}R^{\frac{1}{2}}$$

### Cell Culture

Murine C2C12 myoblasts (Sigma–Aldrich) were maintained in Dulbecco's Modified Eagle Medium (DMEM, Invitrogen) with high glucose content, supplemented with 20% Foetal Bovine Serum (FBS, Invitrogen) and 1% antibiotics (P/S) (1 mL of a mixture of 10 000 units mL^−1^ of penicillin and 10 000 µg mL^−1^ streptomycin per 100 mL of media, ThermoFisher Scientific) in humidified atmosphere at 37 °C and 5% CO^2^.

### Osmolality and Electrical Conductivity

The osmolality of cell culture medium (Dulbecco's Modified Eagle Medium with high glucose content, supplemented with 20% Foetal Bovine Serum and 1% antibiotics P/S) as well as cell culture medium with B at 0.59 and 1.47 mm was measured using freezing point depression with a cryo‐osmometer OSMOMAT model 030.

The electrical conductivity of the cell culture medium described above, as well as the cell culture medium with B at 0.59 and 1.47 mm, was measured using an EC‐Meter BASIC 30 + with a conductivity cell 50 71 (CRISON).

### Cell Viability

Cytotoxicity assay MTT ((3‐(4,5‐dimethylthiazol‐2‐yl)‐2,5‐diphenyltetrazolium bromide) quantitative assay (Promega) was performed to assess the cytocompatibility of PAAm hydrogels and borax (Borax España S.A) with C2C12 cells. 1 × 10^4^ cells per cm^2^ were seeded on PAAm hydrogels, and metabolic activity was measured after 1, 3, 5, and 7 days of incubation in DMEM supplemented with 20% FBS and 1% P/S. Samples were treated with borax 0.59 or 1.47 mm as required. These concentrations are based on previous works from the group.^[^ ^28^, ^29^ ^]^ Cells were then incubated for 2 h with MTT (tetrazolium salt) at 37 °C. Formazan was solubilized with DMSO followed by measuring absorbance at 540 nm. Three biological replicates with three technical replicates were measured.

MTT assays were complemented with LIVE/DEAD viability/cytotoxicity kit (Molecular Probes). Briefly, samples were washed with PBS at 37 °C and incubated for 30 min at 37 °C with a mixture containing 4 µm ethidium homodimer‐1 and 2 µm calcein AM in PBS. Then, the staining solution was removed, and the samples were washed with PBS. Samples were imaged using a ZEISS Axio Observer Z1 epifluorescence inverted microscope. Image processing and analysis were performed using Fiji imaging software.^[^ ^87^ ^]^ 3 biological replicates with three technical replicates were measured.

### Cell Proliferation

1 × 10^4^ cells per cm^2^ were seeded on PAAm hydrogels and allowed to adhere for 24 h. Samples were treated with borax 0.59 or 1.47 mm as required. Cells were incubated with alamarBlue reagent (ThermoFisher Scientific) for 2 h protected from the light in a humidified atmosphere at 37 °C and 5% CO2. Absorbance was read at 570 nm using a Multiskan FC microplate reader (ThermoScientific). 600 nm was used as a reference wavelength. three biological replicates with three technical replicates were measured.

### Cell Adhesion

C2C12 cells were seeded at a low density of 5 × 10^3^ cells per cm^2^ onto functionalized PAAm hydrogels and allowed to adhere for 3 h. Cells were cultured in DMEM with high glucose content, supplemented with 1% P/S and in the absence of serum (FBS). After 3 h of culture, cells were washed in DPBS (Gibco) and fixed in 4% formaldehyde solution (Sigma–Aldrich) for 20 min. Samples were treated with borax 0.59 or 1.47 mm as required. Ten cells from three different biological replicates were measured.

### Immunostaining

Cells from adhesion and differentiation experiments were rinsed with DPBS and permeabilized with 0.5% Triton x‐100 in DPBS at room temperature for 5 min, next blocked in 2% BSA (Sigma–Aldrich) in DPBS for 1 h at room temperature, and then incubated with primary antibodies in blocking solution overnight at 4 °C. The samples were then rinsed twice in DPBS/0.1% Triton X‐100 and incubated with the secondary antibody and phalloidin (Invitrogen) at room temperature for 1 h. Finally, samples were washed twice in 0.1% Triton X‐100 in DPBS before mounting with Vectashield containing DAPI (Vector Laboratories). For cell adhesion studies, a mouse monoclonal primary antibody against vinculin (1:400, Sigma–Aldrich), Alexa fluor 488 phalloidin (1:200, Invitrogen), and rabbit anti‐mouse Cy3‐conjugated (Jackson Immunoresearch, 1:200) secondary antibody were used. For myogenic differentiation studies, a mouse monoclonal primary antibody against sarcomeric α‐actinin (1:200, Abcam) and a rabbit anti‐mouse Cy3‐conjugated secondary antibody (1:200, Jackson Immunoresearch) were used. For intracellular tension studies, mouse monoclonal primary antibody against phospho‐myosin light chain (1:200, Cell Signalling), rabbit monoclonal primary antibody against yes‐associated protein 1 (YAP) (1:500, Abcam), Alexa fluor 488 phalloidin (1:200, Invitrogen) and rabbit anti‐mouse Cy3‐conjugated (1:200, Jackson Immunoresearch) and goat anti‐rabbit Cy3‐conjugated (1:200, Jackson Immunoresearch) secondary antibodies were used.

### Image Acquisition for Fixed Samples

Images of the actin cytoskeleton were captured using either a Zeiss Observer Z1 epifluorescence inverted microscope (Zeiss) with a 20×/0.5NA Plan Fluor objective, 40×/0.6NA Plan Fluor objective and 63×/1.4NA Plan Apo oil immersion objective; or a Zeiss LSM900 confocal microscope (Zeiss) using a 20×/0.8NA Plan Apo objective and 40x/1.3NA Plan Apo oil immersion objective. Vinculin images were acquired using a Zeiss Observer Z1 epifluorescence inverted microscope (Zeiss) with a 63×/1.4NA Plan Apo oil immersion objective or a Zeiss LSM900 confocal microscope (Zeiss) using a 40×/1.3NA Plan Apo oil immersion objective. pMLC images were acquired using a Zeiss Observer Z1 epifluorescence inverted microscope (Zeiss) with a 20×/0.5NA Plan Fluor objective and 40×/0.6NA Plan Fluor objective. YAP images were acquired using a Zeiss Observer Z1 epifluorescence inverted microscope (Zeiss) with a 20×/0.5NA Plan Fluor objective and 40×/0.6NA Plan Fluor objective. Images of the subcellular localization were captured using a Zeiss LSM900 confocal microscope (Zeiss) using a 20×/0.8NA Plan Apo objective and 40×/1.3NA Plan Apo oil immersion objective. Images of the PLA colocalization were captured using a Zeiss LSM900 confocal microscope (Zeiss) using a 40×/1.3NA Plan Apo oil immersion objective. Cell viability images were acquired using a Zeiss Observer Z1 epifluorescence inverted microscope (Zeiss) with a 10×/0.3NA Plan Fluor objective. Images of PAAm hydrogels functionalization were acquired using a Zeiss Observer Z1 epifluorescence inverted microscope (Zeiss) with a 10x/0.3NA Plan Fluor objective, 20×/0.5NA Plan Fluor objective, and 40×/0.6NA Plan Fluor objective. Images of cell senescence were captured using a Zeiss LSM900 confocal microscope (Zeiss) using a 20×/0.8NA Plan Apo objective. Images of NaBC1 silencing were captured using a Zeiss Observer Z1 epifluorescence inverted microscope (Zeiss) with a 10x/0.3NA Plan Fluor objective. Images of VD1 transfection were captured using a Zeiss LSM900 confocal microscope (Zeiss) using a 10X/0.3NA Plan Apo objective and 40×/1.3NA Plan Apo oil immersion objective. Image processing and analysis were performed using Fiji imaging software.^[^ ^87^ ^]^

### Actin Flow

C2C12 cells were transfected using the Neon transfection system (ThermoFisher Scientific) following the manufacturer's protocol. The plasmid used was LifeAct‐GFP (Ibidi). The parameters used to achieve cell transfection were 1650 V, 10 ms, 3 pulses, with 5 µg of DNA. Transfected cells were cultured for 24 h.

Cells were seeded at 1 × 10^4^ cells per cm^2^ on PAAm hydrogels and allowed to adhere for 24 h. Cells were imaged using a Zeiss LSM900 confocal microscope (Zeiss) using a 40x/1.3 Plan Apo oil immersion objective. Images were taken for 4 min at 1 frame every 2 s at 488 nm. Actin flow was determined by kymographs in the Fiji imaging software.^[^ ^87^ ^]^ Samples were treated with borax 0.59 or 1.47 mm as required. Five cells with at least five different flow areas per cell were measured.

### Cells Nanoindentation

Nanoindentation measurements were performed using a fiber–optic based nanoindentation device Chiaro (Optics11) mounted on top of an inverted optical microscope Zeiss Axiovert 200M (Zeiss). Measurements were performed following the standardized protocol described by Ciccone et al.^[^ ^85^ ^]^ using cantilevers with stiffness (k) of 0.020 or 0.022 Nm^−1^ holding spherical tips of radius (R) 3.5 and 3 µm, respectively.

C2C12 myoblasts were plated on functionalized glasses at a density of 1 × 10^4^ cells per cm^2^ and allowed to adhere for 24 h. All measurements were performed at 37 °C using an on‐stage Incubator (Okolab) in a standard culture medium.

For each experimental condition, at least ten cells were indented by performing nine repeated indentations on each single cell, with subsequent indentations being spaced 1 µm apart. For each indentation, the probe moved at a speed of 2 µm s^−1^ over a vertical range of 10 µm.

The forward segment of the collected force–displacement (F–z) curves was analyzed using a custom open‐source software.^[^ ^85^ ^]^ Curves were first filtered using a Savitzky–Golay filter from the SciPy computing stack^[^ ^86^ ^]^ with a window length of 25 nm and a polynomial order of 3 to remove random noise. After, the point where the probe came into contact with the cell (*z*_0_,*F*_0_) was identified with a thresholding algorithm to convert (F–z) curves into force–indentation (F–δ) curves. To quantify the elastic properties of the gels (Young's Modulus E), F–δ curves were fitted with the Hertz model (Equation 1) up to a maximum indentation of δ  =  0.1 *R*. The Poisson's ratio (ν) was taken as 0.5, assuming material's incompressibility.

(2) $$F=\frac{4}{3}\frac{E}{\left(1-v^{2}\right)}\delta^{\frac{3}{2}}R^{\frac{1}{2}}$$

### Brillouin Microscopy

Brillouin microscopy (LifeMachinery) was performed by using a 20× objective to illuminate the sample with a 660 nm laser. The backscattered light was coupled into a single‐mode fiber and analyzed using a VIPA cross‐axis spectrometer. Each pixel in the Brillouin maps came from one Brillouin spectrum. In the VIPA spectrometer, the frequencies of the light were separated in space and imaged on a high‐sensitivity Orca Fusion camera. Both the stoke and anti‐stoke peaks, which correspond to lower and higher energy (compared to Rayleigh) scattered radiation, were fitted with a Lorentzian function. The frequency (Brillouin) shift, measured as the displacement of the Brillouin peaks with respect to the Rayleigh, was taken from the LabView software (Light Machinery). Before the measurements, a first calibration with an acrylic cube and Spectralock software was performed. To minimise the laser signal, the etalon pressure was modified by starting with increments of 100 and then 10. Once the laser peak was minimized, a stripe overlay was performed to ensure that the spectra were well extracted with the optimal Brillouin signal. Finally, the collimator coupling was optimized to increase the signal of the Brillouin peaks. A second calibration was then performed with the sample to locate the laser blobs and a striped overlay was then performed. After this, the background was subtracted by closing the laser shutter and making an average of 10 spectra. The laser shutter was then opened and the Brillouin peaks were selected in the LabView software. To take the maps of the sample, a square was placed in the camera window and the size of the square and the size of the step size between measurements was selected. Ten cells from three different biological replicates were measured.

### Traction Force Microscopy (TFM)

Carboxylate‐modified 0.2 µm FluoSpheres (Life Technologies) were prepared by sonicating the stock for 10 min, then diluted 1:30 in milli‐Q water and further sonicated for 15 min. Immediately after sonication, 1:20 FluoSpheres were incorporated into the PAAm hydrogels on coverslips before functionalization, as previously described.

Cells were seeded at 1 × 10^4^ cells per cm^2^ on PAAm hydrogels and allowed to adhere for 24 h. Using an EVOS FL Auto microscope (Life Technologies) with the incubator set at 37 °C and 5% CO2 at 20× magnification, Z‐stack images were taken through the cells (brightfield channel) and FluoSpheres embedded in the hydrogels (Texas Red channel) before and after cell trypsinization. Cell traction forces were determined using ImageJ software by tracking the displacement of the FluoSpheres and then reconstructing the force field from the displacement data using the iterative particle image velocimetry (PIV) and Fourier transform traction cytometry (FTTC) plugins^[^ ^88^ ^]^ in ImageJ software, respectively. The stress maps obtained were modified in ParaView software to plot more accurate scales. At least 30 cells from 10 different beacons per hydrogel were analyzed.

### Gene Expression

Total RNA was extracted from C2C12 cultured for 4, 8, 24, or 96 h under different experimental conditions using RNeasy Micro Kit (Qiagen). RNA quantity and integrity were measured with a NanoDrop 1000 (ThermoScientific). Then, 500 ng of RNA were reverse transcribed using the QuantiTect Reverse Transcription Kit (Qiagen). Real‐time qPCR was performed using Quantinova SYBR Green PCR kit (Qiagen) and 7500 Real Time PCR system (Applied Biosystems). The reactions were run in triplicate for both technical and biological replicas. The primers used for amplification were designed from sequences found in the GenBank database and included:

### *NaBC1* (Gene ID

269356; Fw: 5′‐GAGGTTCGCTTTGTCATCCTGG‐3′, Rev: 5′‐ATGCCAGTGAGCTTCCCGTTCAG‐3′), *AKT* (Gene ID: 11651; Fw: 5′‐GGACTACTTGCACTCCGAGAAG‐3′, Rev: 5′‐CATAGTGGCACCGTCCTTGATC‐3′), *mTOR* (Gene ID: 56717; Fw: 5′‐AGAAGGGTCTCCAAGGACGACT‐3′, Rev: 5′‐GCAGGACACAAAGGCAGCATTG‐3′), *GDF11* (Gene ID: 14561; Fw: 5′‐ TTTCGCCAGCCACAGAGCAACT‐3′, Rev: 5′‐ CTCTAGGACTCGAAGCTCCATG‐3′), *MyoD* (Gene ID: 17927; Fw: 5′‐GCACTACAGTGGCGACTCAGAT‐3′, Rev: 5′‐TAGTAGGCGGTGTCGTAGCCAT‐3′), *MYOGENIN* (Gene ID: 17928; Fw: 5′‐CCATCCAGTACATTGAGCGCCT‐3′, Rev: 5′‐CTGTGGGAGTTGCATTCACTGG‐3′), *VEGFR* (Gene ID: 14254; Fw: 5′‐ TGGATGAGCAGTGTGAACGGCT‐3′, Rev: 5′‐GCCAAATGCAGAGGCTTGAACG‐3′), *INSR* (Gene ID: 16337; Fw: 5′‐AGATGAGAGGTGCAGTGTGGCT‐3′, Rev: 5′‐ GGTTCCTTTGGCTCTTGCCACA‐3′), *IL‐GFR* (Gene ID: 16001; Fw: 5′‐ CGGGATCTCATCAGCTTCACAG‐3′, Rev: 5′‐TCCTTGTTCGGAGGCAGGTCTA‐3′), *INTEGRIN ALPHA v* (Gene ID: 16410; Fw: 5′‐ GTGTGAGGAACTGGTCGCCTAT‐3′, Rev: 5′‐ CCGTTCTCTGGTCCAACCGATA‐3′), *INTEGRIN ALPHA 5* (Gene ID: 16402; Fw: 5′‐ ACCTGGACCAAGACGGCTACAA‐3′, Rev: 5′‐ CTGGGAAGGTTTAGTGCTCAGTC‐3′), *INTEGRIN ALPHA 7* (Gene ID: 16404; Fw: 5′‐TCTGTCAGAGCAACCTCCAGCT‐3′, Rev: 5′‐ CTATGAACGGCTGCCCACTCAA‐3′), *INTEGRIN BETA 1* (Gene ID: 16412, Fw: 5′‐ CTCCAGAAGGTGGCTTTGATGC‐3′, Rev: 5′‐GTGAAACCCAGCATCCGTGGAA‐3′), *INTEGRIN BETA 3* (Gene ID: 16416, Fw: 5′‐GTGAGTGCGATGACTTCTCCTG‐3′, Rev: 5′‐CAGGTGTCAGTGCGTGTAGTAC‐3′), *INTEGRIN BETA 5* (Gene ID: 16419, Fw: 5′‐ TTTCGCCAGCCACAGAGCAACT‐3′, Rev: 5′‐ CTCTAGGACTCGAAGCTCCATG‐3′). *GAPDH* (Gene ID: 14433; Fw: 5′‐CATCACTGCCACCCAGAAGACTG‐3′, Rev: 5′‐ATGCCAGTGAGCTTCCCGTTCAG‐3′) was used as a housekeeping gene. Ct value was used for quantification by the comparative Ct method. Sample values were normalized to the threshold value of housekeeping gene GAPDH: ∆CT = CT(gene of interest) − CT(GAPDH). The Ct value of the control (cell culture plate) was used as a reference. ∆∆CT = ∆CT(experiment) − ∆CT(control). mRNA expression was calculated by the following equation: fold change = 2^−∆∆CT^. Three biological replicates with three technical replicates were measured.

### NaBC1‐Integrins Colocalization

C2C12 myoblasts were plated on PAAm hydrogels at a density of 1 × 10^4^ cells per cm^2^ and allowed to adhere for 24 h. Samples were treated with borax 0.59 or 1.47 mm as required. Colocalization of NaBC1/Integrin α_v_, NaBC1/α_5_, and NaBC1/β_4_ experiments were performed using DUOLINK PLA system (Sigma–Aldrich) following the manufacturer's instructions. Specific primary antibodies used were: anti‐NaBC1 (Invitrogen, 1:200), anti‐integrin α_v_ (Abcam, 1:500), anti‐integrin α_5_ (Abcam, 1:500) and anti‐integrin β_4_ (Abcam, 1:500). For image quantification of colocalization fluorescent dots, at least 30 individual cells were imaged for each condition using a ZEISS LSM900 confocal microscope.

### Cell Senescence

Cell senescence was measured with the CellEvent Senescence Green Detection Kit (Invitrogen). The kit is based on the CellEvent Senescence Green Probe, which is a fluorescent reagent containing two galactoside moieties, making it specific to the typical senescence marker β‐galactosidase. The enzyme‐cleaved product was retained within the cell due to covalent binding of intracellular proteins and emits a fluorogenic signal that had excitation/emission maxima of 490/514 nm. C2C12 cells were seeded at 1 × 10^4^ cells per cm^2^ on PAAM hydrogels and allowed to adhere for 24 h. Samples were treated with borax 0.59 or 1.47 mm as required. Cells were washed with DPBS (Gibco) and fixed in 4% formaldehyde solution (Sigma–Aldrich) for 20 min. Samples were washed within BSA 1% in DPBS and incubated in the dark with CellEvent Senescence Green Probe for 2 h at 37 °C without CO2. After incubation, samples were washed three times with DPBS, and fluorescence was quantified in a Multiskan FC microplate reader (ThermoScientific) using an Alexa Fluor 488/FITC filter set. Samples were imaged with a Zeiss LSM900 confocal microscope. Three biological replicates with three technical replicates were measured.

### Total ATP Content

C2C12 cells were seeded at 1 × 10^4^ cells per cm^2^ on PAAM hydrogels. After 24 h, cells were washed twice with PBS and lysed using 0.5% Triton‐X100 in PBS. After centrifugation at 11000 × g for 3 min, 5 µL of total lysate were used in triplicates for the assessment of total ATP content using the ATP Determination Kit (ThermoFisher Scientific) following the manufacturer's instructions. Luminescence was monitored at 560 nm using a Multiskan FC microplate reader (ThermoScientific). Three biological replicates with three technical replicates were measured.

### Mitochondrial ATP Content

Mitochondrial ATP content was assessed with the BioTracker ATP‐Red dye (Millipore), a live cell red fluorescent imaging probe for ATP. The probe specifically reports ATP content in the mitochondrial matrix of living cells. The probe without ATP forms a closed‐ring structure that is not fluorescent. In the presence of the negatively charged ATP, the covalent bonds between boron and ribose in the probe are broken and the ring opens, causing the probe to be fluorescent.

C2C12 cells were seeded at 1 × 10^4^ cells per cm^2^ on PAAM hydrogels. After 24 h, cells were incubated for 1 h with 200 nm MTG. Then, cells were washed twice with PBS and incubated for 15 min with 5 µm BioTracker ATP‐Red dye in medium at 37 °C and 5% CO2. Then, the cells were washed twice with medium, and fresh medium was added. Imaging was performed using a ZEISS LSM900 confocal microscope (Zeiss). Analysis was performed with ZEN software by measuring the average ATP red fluorescence intensity inside a region of interest generated by the MTG area. At least ten cells from three different biological replicates were measured.

### Chemical Procedure for the Preparation of Fluorescein‐Labeled Boronic Acid

Chemical synthesis reactions were performed employing commercial reagents and solvents without additional purification unless otherwise noted. Solvents for synthesis were purchased from Scharlab, while chemicals were purchased from usual commercial sources. Particularly, 4‐(Aminomethyl)phenylboronic acid pinacol ester hydrochloride, HCl in methanol (1.25 m), triethylamine, and sodium metaperiodate were purchased from Sigma–Aldrich while Fluorescein isothiocyanate (FITC, 95% mixture of isomers) was purchased from ABCR. Analytical thin‐layer chromatography (TLC) was performed on precoated aluminum silica gel sheets. NMR spectra were recorded in a Bruker AV250 equipment employing deuterated solvents purchased from Sigma–Aldrich. Chemical shifts were reported relative to the remainder ^1^H of solvents. Coupling constants *J* were given in Hz. Resonance patterns were designated with the notations s (singlet), d (doublet), m (multiplet), br s (broad singlet). FTIR spectra were recorded using a Nexus (ThermoFisher Scientific) spectrometer equipped with a Smart Golden Gate ATR accessory. Mass spectrometry spectra were recorded in a Bruker HTC ion trap mass spectrometer.

**Scheme 1:**
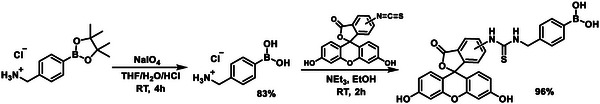
Chemical synthesis of fluorescein‐tagged boronic acid (4‐((3‐(3′,6′‐dihydroxy‐3‐oxo‐3*H*‐spiro[isobenzofuran‐1,9′‐xanthen]‐5/6‐yl)thioureido)methyl)phenyl)boronic acid.

Title compound, 4‐(fluorescein)thioureidomethylphenylboronic acid was prepared in a two‐step procedure from 4‐(aminomethyl)phenylboronic acid pinacol ester (**Scheme** **1**). First, the cleavage of the pinacol ester was achieved upon oxidative cleavage of the pinacol C─C bond. Briefly, NaIO_4_ (240 mg, 1.1 mmol 1.2 equiv.) and the ammonium‐containing boronic ester (250 mg, 0.93 mmol, 1.0 equiv.) were dissolved in a mixture of THF (8 mL), water (2 mL), and hydrochloric acid (0.1 mL). This mixture was stirred at room temperature for 4h. To isolate boronic acid, solvents were removed under a vacuum and the crude dissolved in ethanol. Inorganic salts were filtered off, and the crude was then treated with a solution of hydrogen chloride in methanol (5mL) to reform the hydrochloride. Finally, the intermediate compound was isolated after solvent removal and precipitation with a mixture of chloroform and heptane. The product was isolated as a white solid (145 mg, 83%) and employed for the next step without further purification. ^1^H NMR (250 MHz, MeOD) δ 7.70 (d, *J* = 7.2 Hz, 1H), 7.45 (d, *J* = 7.4 Hz, 1H), 4.12 (s, 1H). MS(ESI^+^), m/z calculated for (M+H)^+^ [C_7_H_11_BNO_2_]^+^: 152.09, found: 152.1. Melting point: >180 °C (decomposition).

The preparation of the title compound was performed by direct reaction of the as‐prepared aminomethyl phenylboronic acid hydrochloride (9.4 mg, 0.05 mmol) with stoichiometric amounts of FITC (19.5 mg, 0.05 mmol) and a stock solution of triethylamine (0.01 m) in EtOH (5 mL). The reaction was stirred for 2 h in the dark, evaporated, and filtered through a flash chromatography column employing silica gel and EtOAc as eluent. The final compound was obtained as an orange solid (26 mg, 96%). ^1^H NMR (250 MHz, MeOD) δ 8.05 (d, *J* = 1.8 Hz, 1H), 7.75 (dd, *J* = 8.2, 2.0 Hz, 1H), 7.62 (br. d, *J* = 7.2 Hz, 2H), 7.38 (d, *J* = 7.9 Hz, 2H), 7.21 (d, *J* = 8.5 Hz, 1H), 7.07–6.98 (m, 3H), 6.74–6.51 (m, 7H), 4.88 (s, 2H). MS(ESI^+^), m/z calculated for (M+H)^+^ [C_28_H_21_BN_2_O_7_S]^+^: 540.12, found: 540.1.

### Subcellular Localization

C2C12 myoblasts were plated on PAAm hydrogels at a density of 1 × 10^4^ cells per cm^2^ and allowed to adhere for 24 h. Cells were treated with FITC‐B for 1 h at 37 °C. After washing with DPBS, cells were incubated with 75 nm LysoTracker Red DND‐99 (Invitrogen), 100 nm MitoTracker Red CMXRos (Invitrogen), or 1 µm ER‐tracker Red (Invitrogen) for 1 h at 37 °C. Samples were imaged with a Zeiss LSM980 confocal microscope and an incubator to maintain the conditions constant at 37 °C and 5% CO2. Mander's overlapping coefficient (MOC) was determined with ZEN software. 20 cells from 3 different biological replicates were measured.

### Fluorescence Recovery After Photobleaching (FRAP)

C2C12 myoblasts were plated on PAAm hydrogels at a density of 1 × 10^4^ cells per cm^2^ and allowed to adhere for 24 h. Samples were treated with FITC‐B for 1 h, as required. Then, samples were washed with DPBS three times and imaged with a Zeiss LSM980 confocal microscope. 488 laser was used to bleach 100% of fluorescence in the indicated areas (nucleus, cytoplasm, mitochondria, lysosomes, endoplasmic reticulum, and focal adhesions) for 2 ms. Fluorescence recovery was measured for 4 min after bleaching. Ten cells from three different biological replicates were measured.

### Myogenic differentiation

C2C12 cells were plated on PAAm hydrogels at a high seeding density of 2 × 10^4^ cells per cm^2^ in differentiation medium for myotube formation (DMEM high glucose content supplemented with 1% Insulin–Transferrin–Selenium (ITS, Gibco) and 1% P/S. Samples were treated with borax 0.59 or 1.47 mm as required. The differentiation medium was changed every 2 days. After 4 days of culture, cells were washed in DPBS (Gibco) and fixed in 4% formaldehyde solution (Sigma–Aldrich) for 20 min. Three biological replicates with three technical replicates were measured.

### AlphaFold 3 Predictions

Complexes structures of mouse NaBC1 (A2AJN7) and mouse β_1_ integrin (P09055‐1) transmembrane and intracellular domains, and mouse NaBC1 (A2AJN7) with mouse vinculin (Q64727) were generated using AlphaFold 3^[^ ^89^ ^]^ with two copies of each protein, along with two sodium and borate ions. Representative models from each set of 5 AlphaFold model predictions were selected, and images were generated using Molstar Viewer.^[^ ^90^ ^]^

### Co‐Immunoprecipitation and Immunoblotting

Co‐immunoprecipitation was performed following the protocol described elsewhere.^[^ ^91^ ^]^ Briefly, rigid PAAM hydrogels were fabricated, and cells were cultured on these hydrogels with or without media containing B (1.47 mm) for 24 h to assess NaBC1/β_1_ and NaBC1/Vinculin. After 24 h, cells were lysed in co‐immunoprecipitation lysis buffer containing protease and phosphatase inhibitors at a final concentration of 1X. Antibodies specific for β_1_ integrin (Proteintech) and vinculin (Proteintech) were conjugated to Dynabeads beads coated with protein A or G (ThermoFisher) and then incubated with equal amounts of protein lysates (2 mg) to allow formation of protein–antibody complexes which was then eluted from the beads by using reducing agent (ThermoFisher) and sample buffer (ThermoFisher) at final concentration of 1×, followed by heating at 95 °C for 10 min. Electrophoresis was then performed at 190 V for 50 min at room temperature, and proteins were transferred to PVDF membrane at 20 V and 160 mA for 70 min in a cooled environment, followed by blocking using 5% non‐fat dry milk (NFDM) and immunodetection using protein‐specific antibodies for 1.5 h at room temperature. Membranes were washed thrice using Tris‐buffered saline containing 0.1% tween‐20 and then incubated with HRP‐conjugated secondary antibodies diluted in 5% NFDM for 1 h at room temperature. Proteins were then imaged using a chemiluminescence substrate. Mouse IgG_1_ and rabbit IgG were used as control antibodies. To identify protein‐specific bands, 40 µg protein lysate was run alongside eluted proteins and referred to as input. NaBC1 antibody used was purchased from Merck. The use of Protein ladders PL00003 (Proteintech) and 26634 (ThermoFisher) is indicated in the figure captions.

### Silver Staining

Silver staining was performed to visualize all proteins present in the immunocomplexes after immunoprecipitation. β_1_ integrin or vinculin‐specific antibodies were first bound to Dynabeads beads coated with protein A or G and then added to cell lysates to allow formation of bead–antibody–protein complex. Proteins were then eluted, and electrophoresis was performed following the procedure mentioned above. Silver staining was performed following the manufacturer's instructions (ThermoFisher). Briefly, the gel was washed thrice in de‐ionized water (DI water) and fixed twice using ethanol and acetic acid (30% ethanol, 10% acetic acid) for 15 min. After three washes using DI water, the gel was immersed in a sensitizer solution for 1 min. The gel was then stained for 30 min by immersing it in a staining solution and finally developed for 30 s. The development was stopped by adding 5% acetic solution for 10 min. The gel was washed several times using DI water and was finally imaged.

### NaBC1 Silencing

C2C12 cells were seeded at 6 × 10^4^ cells per cm^2^ on PAAm hydrogels in DMEM with high glucose content, supplemented with 20% FBS and 1% P/S in humidified atmosphere at 37 °C and 5% CO2. After 24 h, cells were washed with Opti‐MEM reduced serum medium (ThermoFisher Scientific) and transfected using pre‐designed MISSION esiRNA (Sigma–Aldrich) against mouse NaBC1 in X‐tremeGENE siRNA Transfection Reagent (Roche), following manufacturer's instructions. MISSION esiRNA Fluorescent Universal Negative Control 1 Cy3 (NC, Sigma–Aldrich) was used as a control of transfection efficiency. NaBC1 silencing was corroborated by the evaluation of NaBC1 mRNA expression levels.

### VD‐1 Transfection

C2C12 cells were transfected using the Neon transfection system (ThermoFisher Scientific) following the manufacturer's protocol. The plasmid used was VD‐1‐GFP (kindly gifted by Pere Roca‐Cusachs). The parameters used to achieve cell transfection were 1650 V, 2 ms, 3 pulses, with 5 µg of DNA. Transfected cells were cultured for 24 h. Following VD‐1 transfection, cell morphology, immunostaining, and actin flow assays were performed, as previously described.

### Image Analysis

For PAAm hydrogels functionalization measurement, staining intensity of immunofluorescence images (fibronectin) was quantified by Fiji imaging software.^[^ ^87^ ^]^

Focal adhesions were analyzed in Fiji imaging software^[^ ^87^ ^]^ by following a previously published protocol. Briefly, images were cropped to include only one cell per image. Subsequently, background subtraction was performed using a sliding paraboloid and rolling ball radius of 50. Local contrast was then enhanced by applying CLAHE using block size = 19, histogram bins = 256, and maximum slope = 6. The image underwent an exponential transformation to minimize the background. Automatic adjustments were made to image brightness and contrast, followed by the application of a Log3D filter with sigmax = sigmay = 3 applied. The LUT of the image was inverted, and automatic threshold was applied to convert the image to a binary. A watershed algorithm was then used to eliminate incorrectly clustered adhesions. Finally, the “Analyze Particles” command was executed (minimum 20 pixels to maximum infinity) to quantify FAs area and count per cell.

Cell projected area, circularity, and aspect ratio were quantified in Fiji imaging software^[^ ^87^ ^]^ by applying a Gaussian blur filter (sigma = 2), followed by a default threshold to segment individual cells and quantify parameters of interest. The threshold was manually adjusted to capture the entire cell cytoskeleton.

For intracellular tension studies, staining intensity of immunofluorescence images (phospho–myosin light chain) was quantified by Fiji imaging software.^[^ ^87^ ^]^

YAP expression was plotted as a nuclear/cytoplasmic ratio; this was performed by measuring nuclear and cytoplasmic YAP expression independently using Fiji imaging software^[^ ^87^ ^]^ and calculated as follows:

First, the cytoplasmic area was defined, where A*cell* is the area of the entire cell and A*nuc* is the area of the cell nucleus. A*nuc* was calculated by applying a Gaussian blur (sigma = 2) and segmenting nuclei (DAPI channel) via the default threshold. Subsequently, A*cell* was calculated by applying a Gaussian blur (sigma = 2) and segmenting the actin cytoskeleton (Phalloidin channel) via the default threshold.

(3) Acyt=Acell−Anuc

Second, YAP's integrated density fluorescence in the cytoplasm was calculated, where YAP*cell* is the integrated density of YAP in the entire cell and YAP*nuc* is the integrated density of YAP in the cell nucleus

(4) YAPcyt=YAPcell−YAPnuc

Finally, YAP's integrated density fluorescence nucleus/cytoplasm ratio is calculated, where YAP*nuc* is the integrated density of YAP in the nucleus, A*nuc* is the area of the nucleus, YAP*cyt* is the integrated density of YAP in the cytoplasm, and A*cyt* is the area of the cell cytoplasm.

(5) $$YAPnuc/cyt\;ratio=\left[\left(YAPnuc/Anuc\right)/\left(YAPcyt/Acyt\right)\right]$$

To determine the retrograde actin flow at the cell edge, time‐lapses were initially converted to 8‐bit format, underwent background subtraction, contrast enhanced, and were subjected to a Gaussian blur filter (sigma = 1.5). Subsequently, kymographs were generated using the Multi Kymograph plugin in Fiji imaging software^[^ ^87^ ^]^ with a line width of 1. Actin retrograde flow speed was then computed using the bounding rectangle parameters as follows:

(6) $$v=\frac{width/c1}{height/c2}$$

where width is the width of the bounding rectangle in pixels, c1 is the spatial conversion factor (px µm^−1^), height is the height of the bounding rectangle in pixels, and c2 is the temporal conversion factor (px s^−1^). This calculation yields the actin retrograde flow speed (v) in units of µm s^−1^, which is subsequently converted to nm s^−1^.

For myogenic differentiation analysis, total nuclei per image were counted from myotube images from differentiation experiments using the “Analyze particles” command in Fiji imaging software.^[^ ^87^ ^]^ The segmented DAPI channel image was subtracted from the Cy3 channel segmented image, and the remaining nuclei were counted and assigned to non‐differentiated cells. The fusion index expressed in %, was calculated subtracting the non‐differentiated nuclei from the total nuclei counted. Myotubes were only considered when 3 or more nuclei were aligned inside cells.

### Statistical Analysis

All statistical analyses were performed in Prism v9 software (GraphPad). Data are presented as Mean ± Standard Deviation. Normality tests were performed to determine whether to select parametric or non‐parametric tests. Two‐tailed unpaired Student's *t* tests were employed when comparing two conditions. For comparisons involving more than two conditions with variations in one or two variables, one‐way ANOVA or two‐way ANOVA were performed respectively, with post‐hoc Tukey's multiple comparisons test. Specific tests conducted for each analysis, sample size (n), probability (*p*) value, data presentation and the meaning of the significance symbols are detailed in the respective figure captions. Comparisons were considered significant when the *p* value was at least smaller than 0.05. Statistical differences were defined by *p* values and confidence intervals were indicated with a *; * ≤ 0.05, ** ≤ 0.01, *** ≤ 0.001, **** ≤ 0.0001.

## Conflict of Interest

The authors declare no conflict of interest.

## Author Contributions

J.G.‐V., P.R and M.S‐S conceived the project. J.G.‐V. designed, performed and analyzed most of the experiments. G.C. performed and analyzed all nanoindentation experiments and developed the software for Brillouin analysis. U.D. designed, performed, and analyzed all immunoprecipitation and immunoblotting experiments. U.D. also coordinated the wet‐lab results with authors that culminated in AlphaFold3 predictions T.Q. and G.M. designed, performed, and analyzed the AlphaFold3 predictions. E.B.‐E. contributed to the actin flow experiments and Brillouin experiments. A.R.‐N. contributed to cell transfection with LifeAct‐GFP and VD1 plasmids. R.R.C. designed and synthesized Fluorescein‐labelled boronic acid. J.G.‐V, G.C. and M.S‐S wrote the manuscript with contribution from U.D. at R1. All authors read the manuscript. P.R and M.S‐S supervised the project and acquired the funding.

[Correction added on 06 May 2025, after first online publication: Authors contributions section has been added.]

## Supporting information



Supporting Information

Supplemental Video 1

Supplemental Video 2

Supplemental Video 3

Supplemental Video 4

Supplemental Video 5

Supplemental Video 6

Supplemental Video 7

Supplemental Video 8

Supplemental Video 9

## Data Availability

The data that support the findings of this study are available from the corresponding author upon reasonable request.
