# Supplementary material for: NaBC1 Boron Transporter Enables Myoblast Response to Substrate Rigidity via Fibronectin‐Binding Integrins
Source: Adv Sci (Weinh). 2025 Apr 24;12(20):2407548. doi: 10.1002/advs.202407548 (PMC12120709; doi:10.1002/advs.202407548)
Supplement: Supplementary file 1 — Supporting Information [file ADVS-12-2407548-s010.docx]

NaBC1 Boron Transporter Enables Myoblast Response to Substrate Rigidity via Fibronectin-Binding Integrins

*Juan Gonzalez-Valdivieso^1,2^, Giuseppe Ciccone^1,3^, Udesh Dhawan^1^, Tezz Quon^4^, Eva Barcelona-Estaje^1^, Aleixandre Rodrigo-Navarro^1,3^, Rafael R. Castillo^5,6^, Graeme Milligan^4^, Patricia Rico^7,8*^, Manuel Salmeron-Sanchez^1,3,9*^*

^1^ Centre for the Cellular Microenvironment (CeMi), University of Glasgow, Glasgow, United Kingdom

^2^ University of Valladolid, Valladolid, Spain

^3^ Institute for Bioengineering of Catalonia (IBEC), The Barcelona Institute for Science and Technology (BIST), Barcelona, Spain.

^4^ Centre for Translational Pharmacology, School of Molecular Biosciences, College of Medical, Veterinary and Life Sciences, University of Glasgow, United Kingdom

^5^ Universidad de Alcalá, Departamento de Química Orgánica y Química Inorgánica, Instituto de Investigación Química “Andrés M. del Río” (IQAR), 28805-Alcalá de Henares, Madrid, Spain.

^6^ Grupo DISCOBAC, Instituto de Investigación Sanitaria de Castilla-La Mancha (IDISCAM), Spain

^7^ Centre for Biomaterials and Tissue Engineering (CBIT), Universitat Politècnica de València, Valencia, Spain

^8^ Biomedical Research Networking Center in Bioengineering, Biomaterials and Nanomedicine (CIBER-BBN), Spain

^9^ Institució Catalana de Recerca i Estudis Avançats (ICREA), Barcelona, Spain.

Supporting Information

**Table S1.** **Addition of B to cell culture medium do not affect cell osmolality.** Quantification of osmolality and electrical conductivity of cell culture medium (Dulbecco’s Modified Eagle Medium with high glucose content, supplemented with 20% Foetal Bovine Serum and 1% antibiotics P/S) as well as cell culture medium with B at 0.59 and 1.47 mM. *n*: 3 replicates. Data are represented as Mean ± Standard Deviation.

| **Treatment** | **Osmolality (mOsm/Kg)** | **Electrical conductivity (S/m)** |
| --- | --- | --- |
| Untreated | 342 ± 4 | 1.381 ± 0.005 |
| B 0.59 mM | 347 ± 3 | 1.373 ± 0.003 |
| B 1.47 mM | 350 ± 1 | 1.365 ± 0.006 |


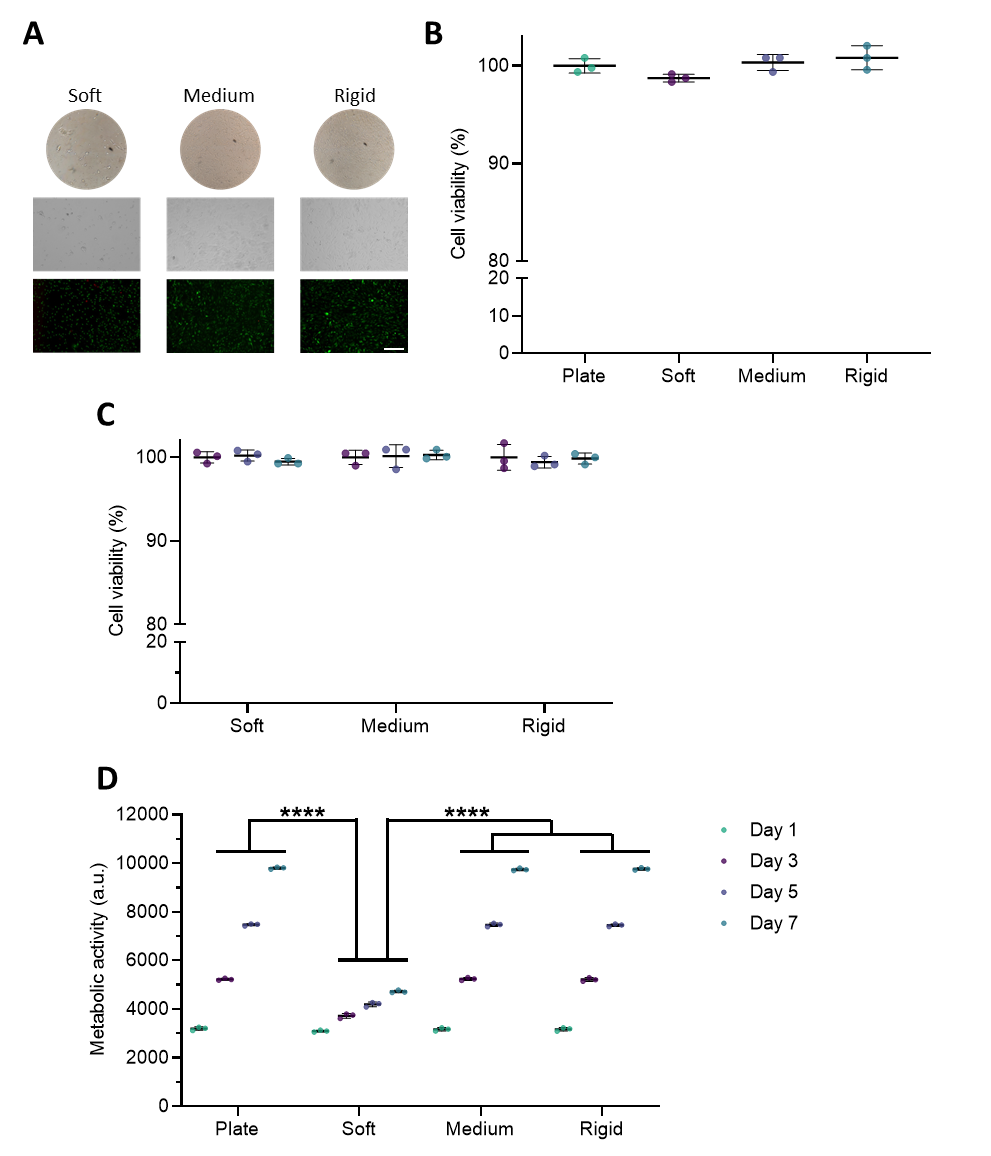


**Figure S1.** **PAAm hydrogels present excellent biocompatibility.** A: Representative images of C2C12 myoblasts seeded on PAAm hydrogels with different stiffness functionalized with fibronectin. Red: ethidium homodimer-1; Green: calcein AM. Scale bar: 100 µm. B: Quantification of cell viability of C2C12 myoblasts seeded on PAAm hydrogels with different stiffness functionalized with fibronectin. *n*: 3 biological replicates with 3 technical replicates. C: Quantification of cell viability of C2C12 myoblasts seeded on PAAm hydrogels with different stiffness functionalized with fibronectin and stimulated with soluble boron (0.59 and 1.47 mM). *n*: 3 biological replicates with 3 technical replicates. D: Quantification of cell proliferation of C2C12 myoblasts seeded on PAAm hydrogels with different stiffness functionalized with fibronectin and stimulated with soluble boron (0.59 and 1.47 mM) for up to 7 days. *n*: 3 biological replicates with 3 technical replicates. Data are represented as Mean ± Standard Deviation, and differences are considered significant for p ≤ 0.05 using one-way ANOVA (Tukey’s multiple comparisons tests) for multiple comparisons. ****p ≤ 0.0001


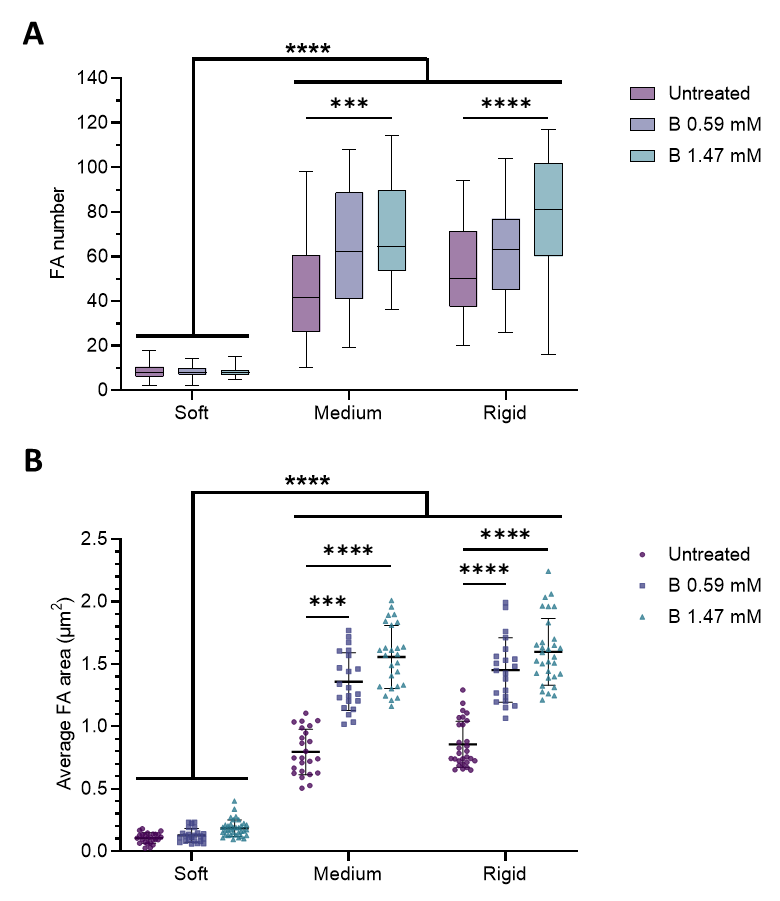


**Figure S2. NaBC1 stimulation triggers the formation of FA in myoblasts on fibronectin.** Quantification of the number (A) and average area (B) of focal adhesions in C2C12 myoblasts seeded on PAAm hydrogels with different stiffness functionalized with fibronectin and stimulated with soluble boron (0.59 and 1.47 mM). *n* = 10 cells from 3 different biological replicates. Data are represented as Mean ± Standard Deviation, and differences are considered significant for p ≤ 0.05 using two-way ANOVA (Tukey’s multiple comparisons tests) for multiple comparisons. ***p ≤ 0.001, ****p ≤ 0.0001


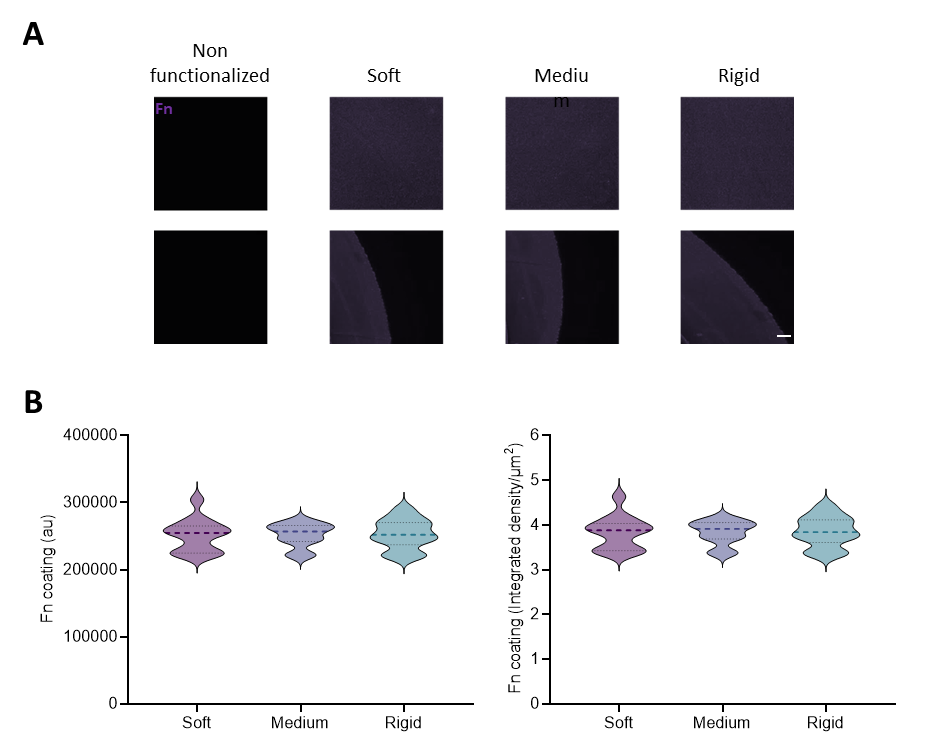


**Figure S3.** **Fibronectin functionalization of PAAm hydrogels is homogeneous in all rigidities.** A: Representative images of PAAm hydrogels with different stiffness functionalized with fibronectin. Magenta: fibronectin. Scale bar: 200 µm. B: Quantification of fibronectin coating of PAAm hydrogels with different stiffness. *n*: 3 biological replicates with 3 technical replicates. Data are represented as Mean ± Standard Deviation, and differences are considered significant for p ≤ 0.05 using one-way ANOVA (Tukey’s multiple comparisons tests) for multiple comparisons.


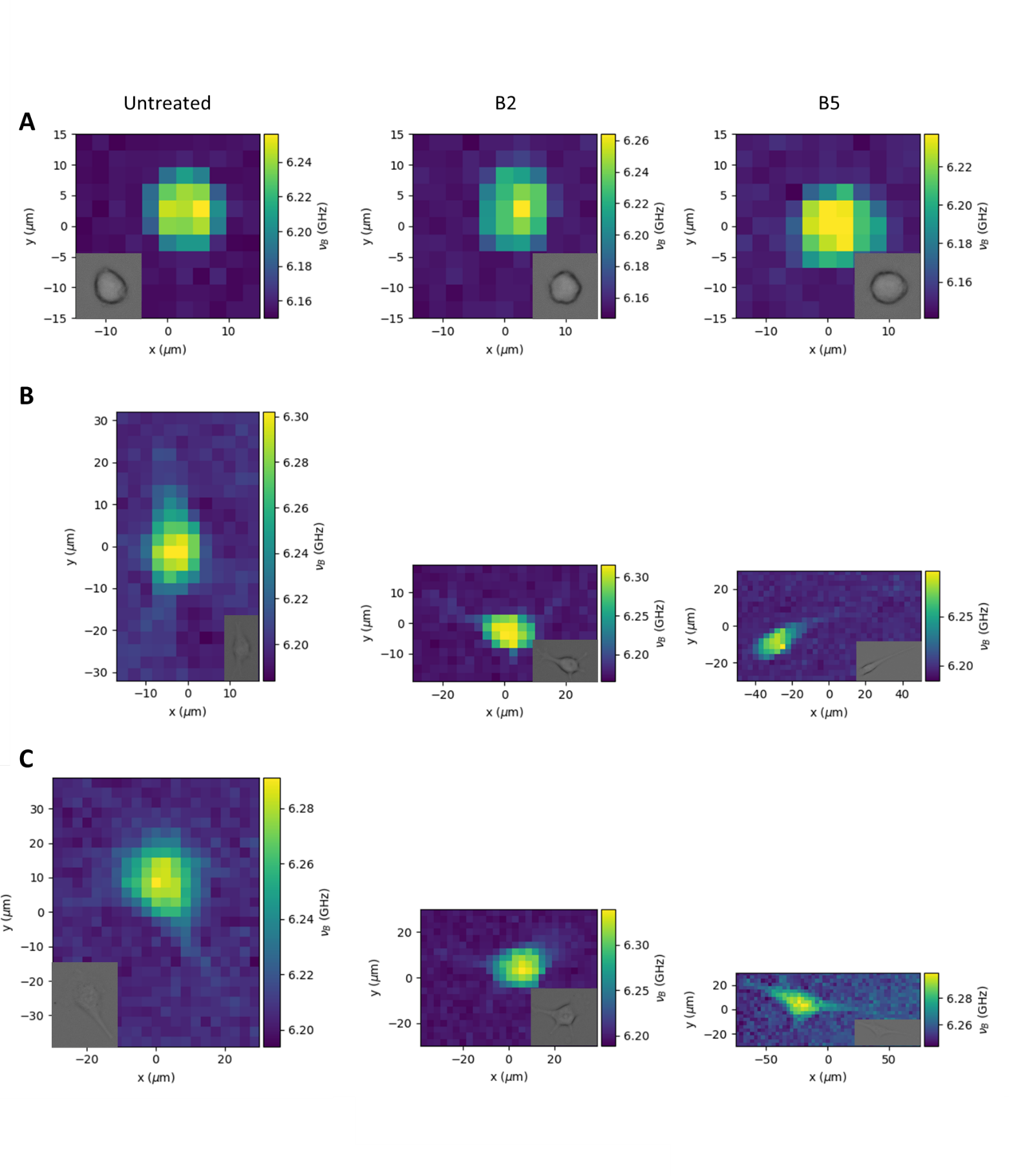


**Figure S4. NaBC1 increases cell stiffness on fibronectin in dependence of substrate rigidity.** Representative Brillouin maps of C2C12 myoblasts seeded on PAAm hydrogels with different stiffness functionalized with fibronectin and stimulated with soluble boron ions (0.59 and 1.47 mM).


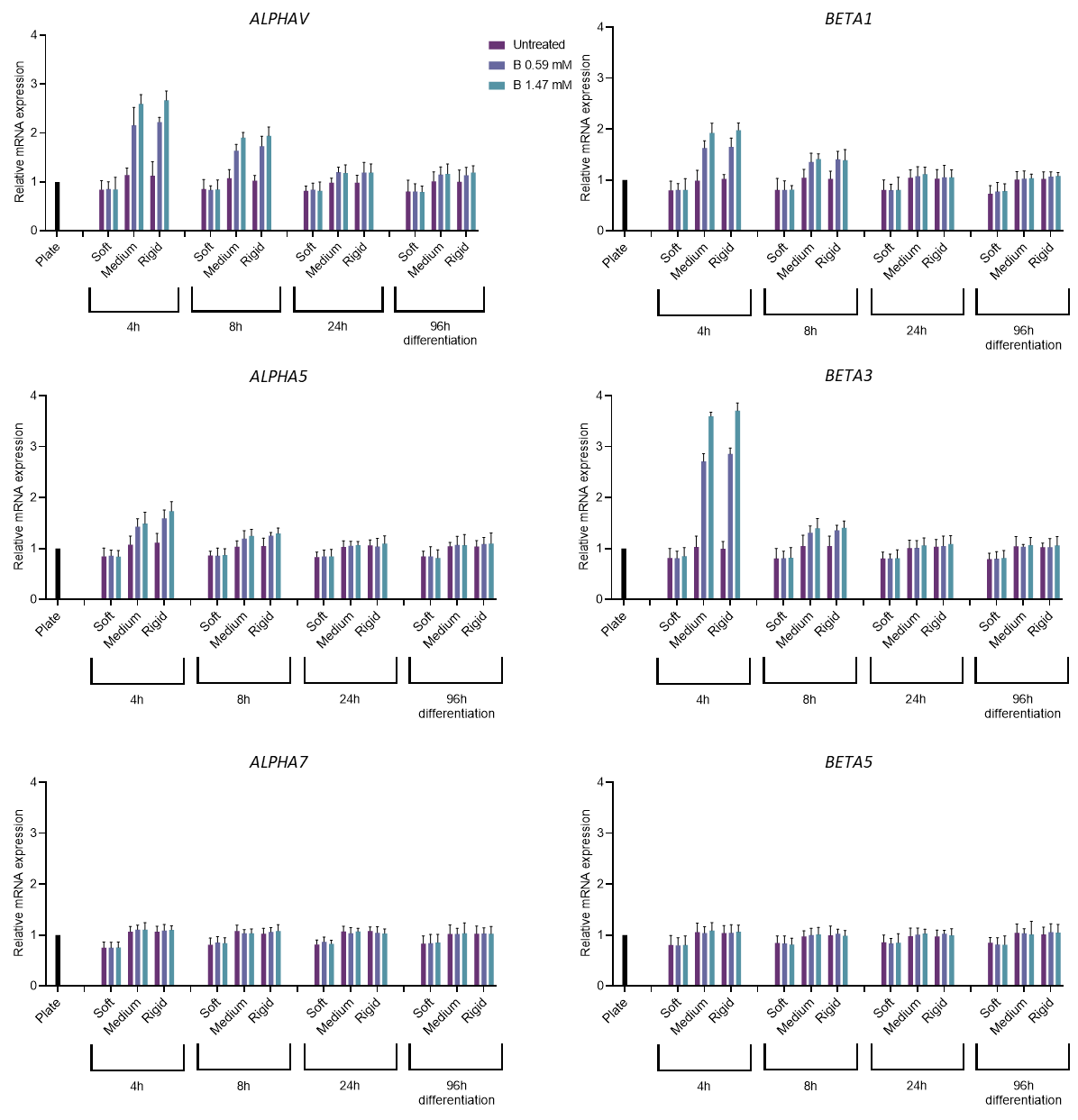


**Figure S5.** **NaBC1 controls intracellular signaling via cooperation with fibronectin-binding integrins.** Quantification of gene expression of cell adhesion-related genes (*ALPHAV*, *ALPHA5*, *ALPHA7*, *BETA1*, *BETA3*, *BETA5* integrins) in C2C12 myoblasts seeded on PAAm hydrogels of different stiffnesses, functionalized with fibronectin and stimulated with soluble boron (B) (at 0.59 and 1.47 mM) for 4, 8, 24 hours or for 96 hours in myogenic differentiation conditions compared to untreated cells on cell culture plates, as measured by qPCR. *n* = 3 biological replicates with 3 technical replicates. Data are represented as Mean ± Standard Deviation.


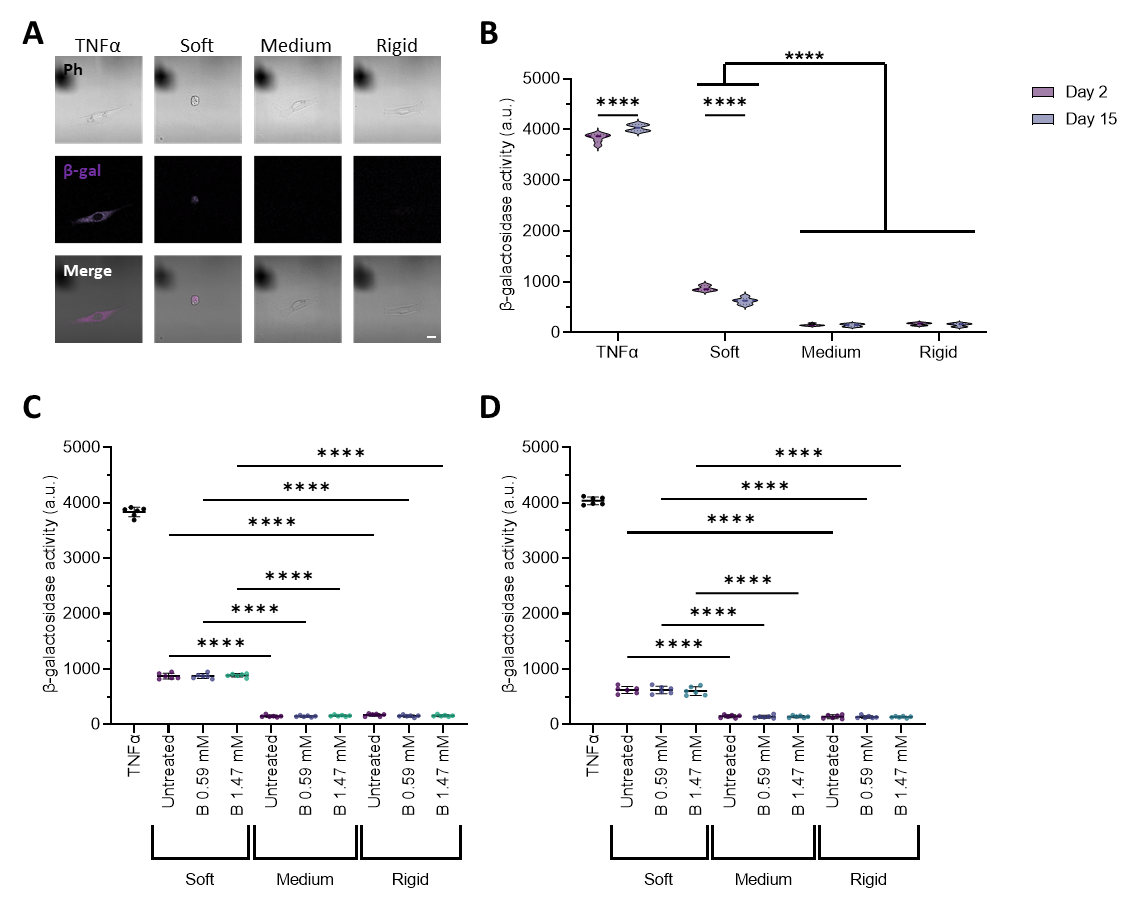


**Figure S6. Cells undergo senescence on soft substrates and it is not reverted by NaBC1 stimulation.** A: Representative images of C2C12 myoblasts seeded on PAAm hydrogels with different stiffness functionalized with fibronectin. Gray: Phase contrast. Magenta: β-galactosidase. Scale bar: 20 µm B: Quantification of β-galactosidase activity in C2C12 myoblasts seeded on PAAm hydrogels with different stiffness functionalized with fibronectin for up to 15 days. *n*: 3 biological replicates with 3 technical replicates. C: Quantification of β-galactosidase activity in C2C12 myoblasts seeded on PAAm hydrogels with different stiffness functionalized with fibronectin and stimulated with soluble boron ions (0.59 and 1.47 mM). *n*: 3 biological replicates with 3 technical replicates. Data are represented as Mean ± Standard Deviation, and differences are considered significant for p ≤ 0.05 using one-way ANOVA or two-way ANOVA (Tukey’s multiple comparisons tests) for multiple comparisons. ****p ≤ 0.0001


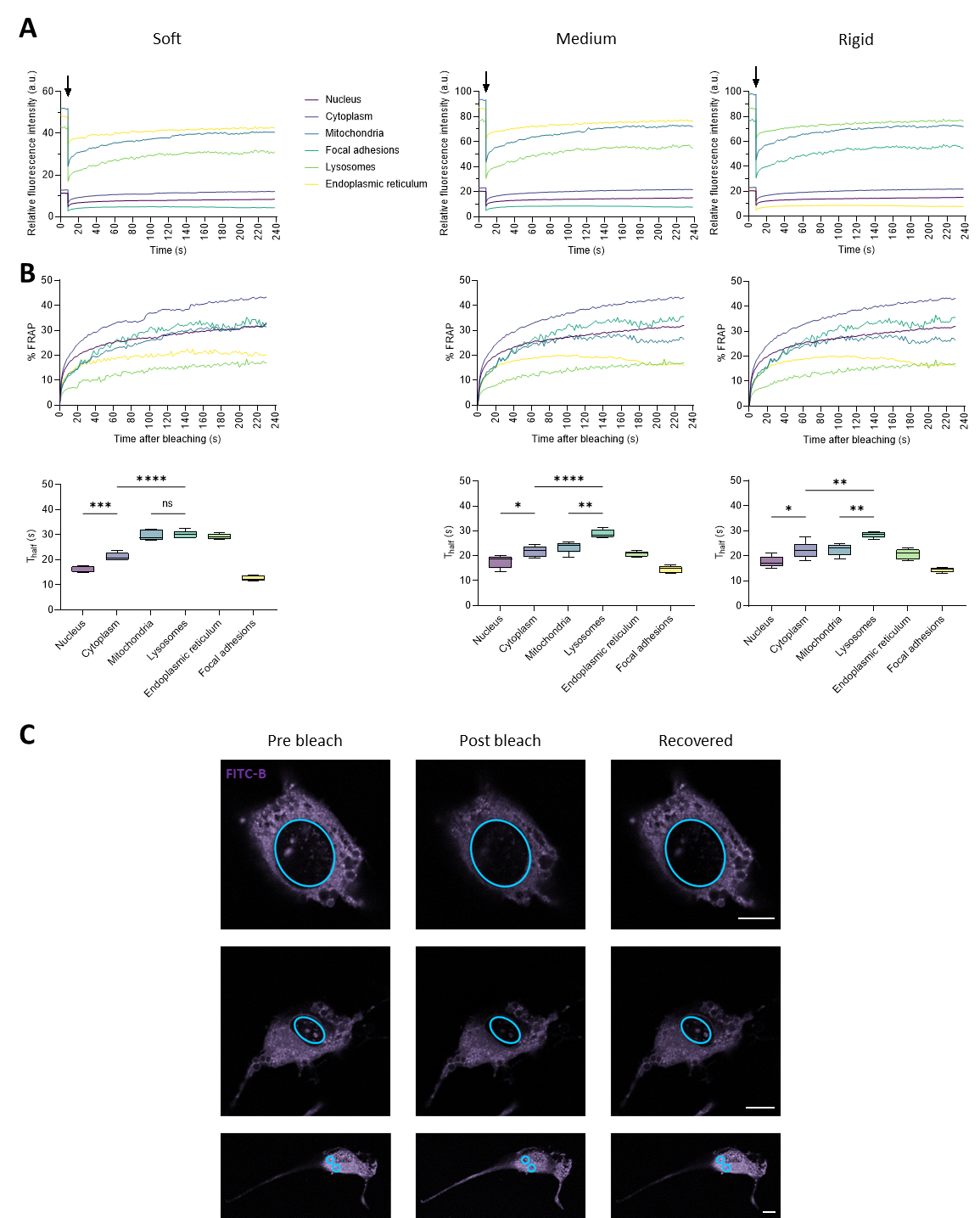


**Figure S7. B subcellular localization and dynamics.** A: Above: profiles of FITC-labelled B in C2C12 myoblasts seeded on PAAm hydrogels with different stiffness functionalized with fibronectin stimulated with soluble boron. Arrows indicate bleaching time. Below: percentage of signal recovered after bleaching. B: Quantification of half-life of FITC-labelled B in C2C12 myoblasts. *n* = 10 cells from 3 different biological replicates. C: Representative images of C2C12 myoblasts seeded on PAAm hydrogels with different stiffness functionalized with fibronectin stimulated with soluble boron ions. Magenta: FITC-labelled B; Cyan circles: bleached area. Scale bars: 20 µm. Data are represented as Mean ± Standard Deviation, and differences are considered significant for p ≤ 0.05 using one-way ANOVA (Tukey’s multiple comparisons tests) for multiple comparisons. *p ≤ 0.05, **p ≤ 0.01, ***p ≤ 0.001, ****p ≤ 0.0001


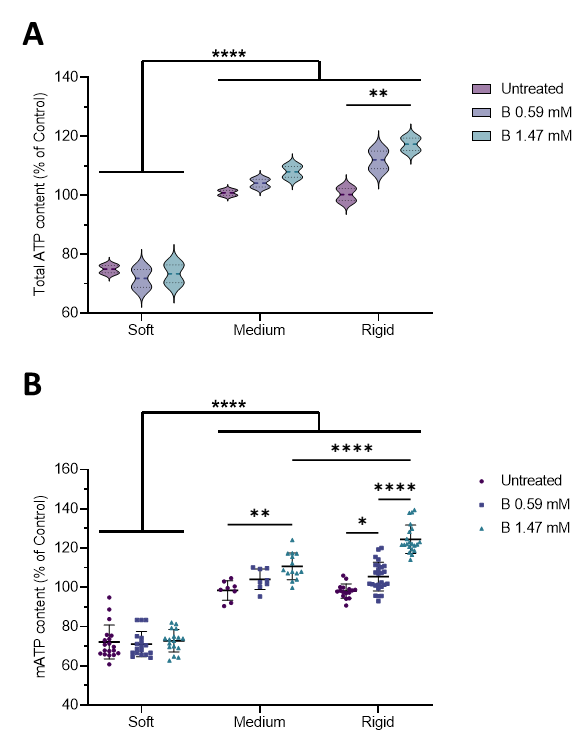


**Figure S8.** **NaBC1 increases total and mitochondrial ATP content on fibronectin in dependence of substrate stiffness.** A: Quantification of total content of C2C12 myoblasts seeded on PAAm hydrogels with different stiffness functionalized with fibronectin and stimulated with soluble boron (0.59 and 1.47 mM). *n*: 3 biological replicates with 3 technical replicates. B: Quantification of mitochondrial content of C2C12 myoblasts seeded on PAAm hydrogels with different stiffness functionalized with fibronectin and stimulated with soluble boron (0.59 and 1.47 mM). *n*: at least 10 cells from 3 biological replicates. Data are represented as Mean ± Standard Deviation, and differences are considered significant for p ≤ 0.05 using two-way ANOVA (Tukey’s multiple comparisons tests) for multiple comparisons. *p ≤ 0.05, **p ≤ 0.01, ****p ≤ 0.0001


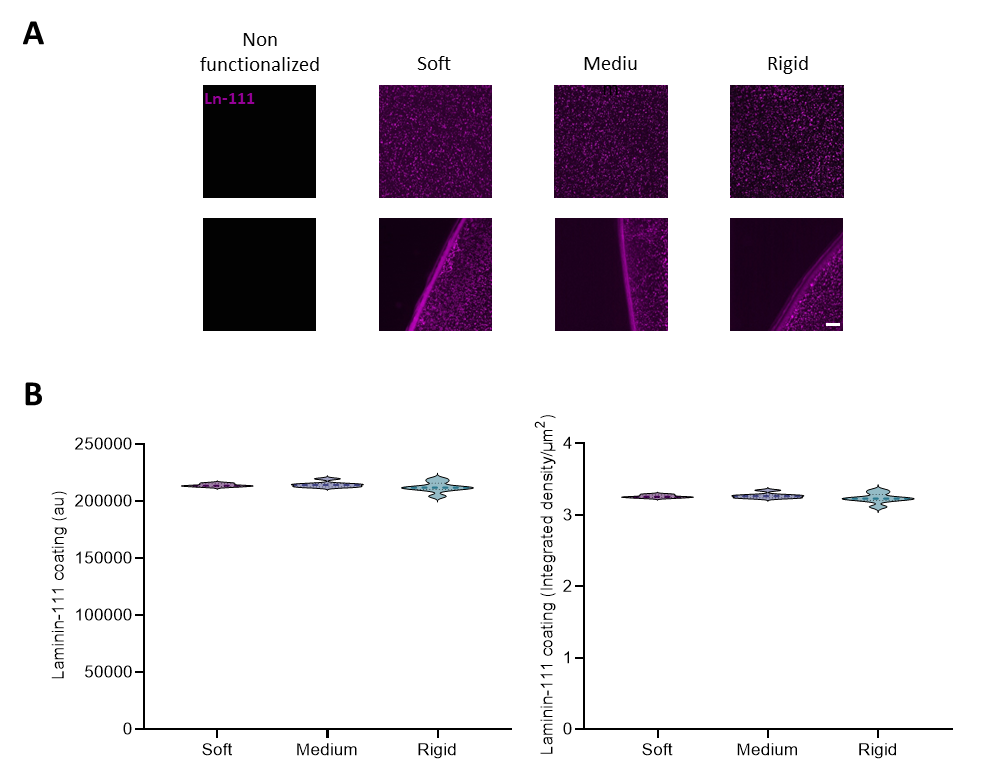


**Figure S9.** **Laminin-111 functionalization of PAAm hydrogels is homogeneous in all rigidities.** A: Representative images of PAAm hydrogels with different stiffness functionalised functionalized with laminin-111. Magenta: laminin-111. Scale bar: 200 µm. B: Quantification of laminin-111 coating of PAAm hydrogels with different stiffness. n: 3 biological replicates with 3 technical replicates. Data are represented as Mean ± Standard Deviation, and differences are considered significant for p ≤ 0.05 using one-way ANOVA (Tukey’s multiple comparisons tests) for multiple comparisons.


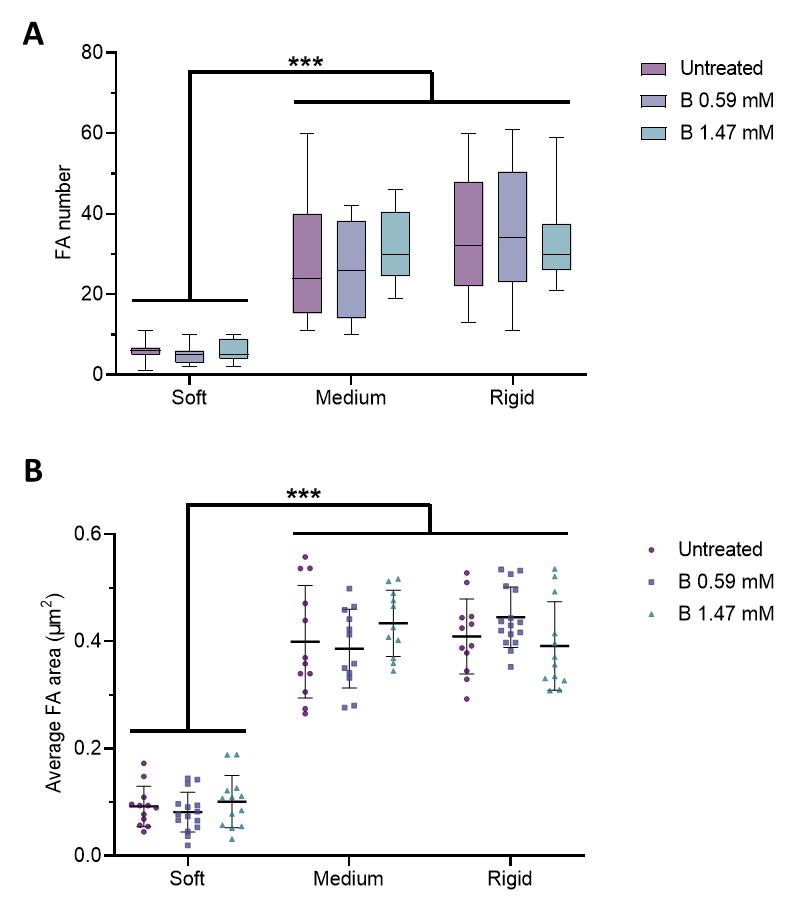


**Figure S10. The formation of FA in myoblasts on laminin-111 is not enhanced by NaBC1 stimulation.** Quantification of the number (A) and average area (B) of focal adhesions in C2C12 myoblasts seeded on PAAm hydrogels with different stiffness functionalized with laminin-111 and stimulated with soluble boron ions (0.59 and 1.47 mM). *n* = 10 cells from 3 different biological replicates. Data are represented as Mean ± Standard Deviation, and differences are considered significant for p ≤ 0.05 using two-way ANOVA (Tukey’s multiple comparisons tests) for multiple comparisons. ***p ≤ 0.001


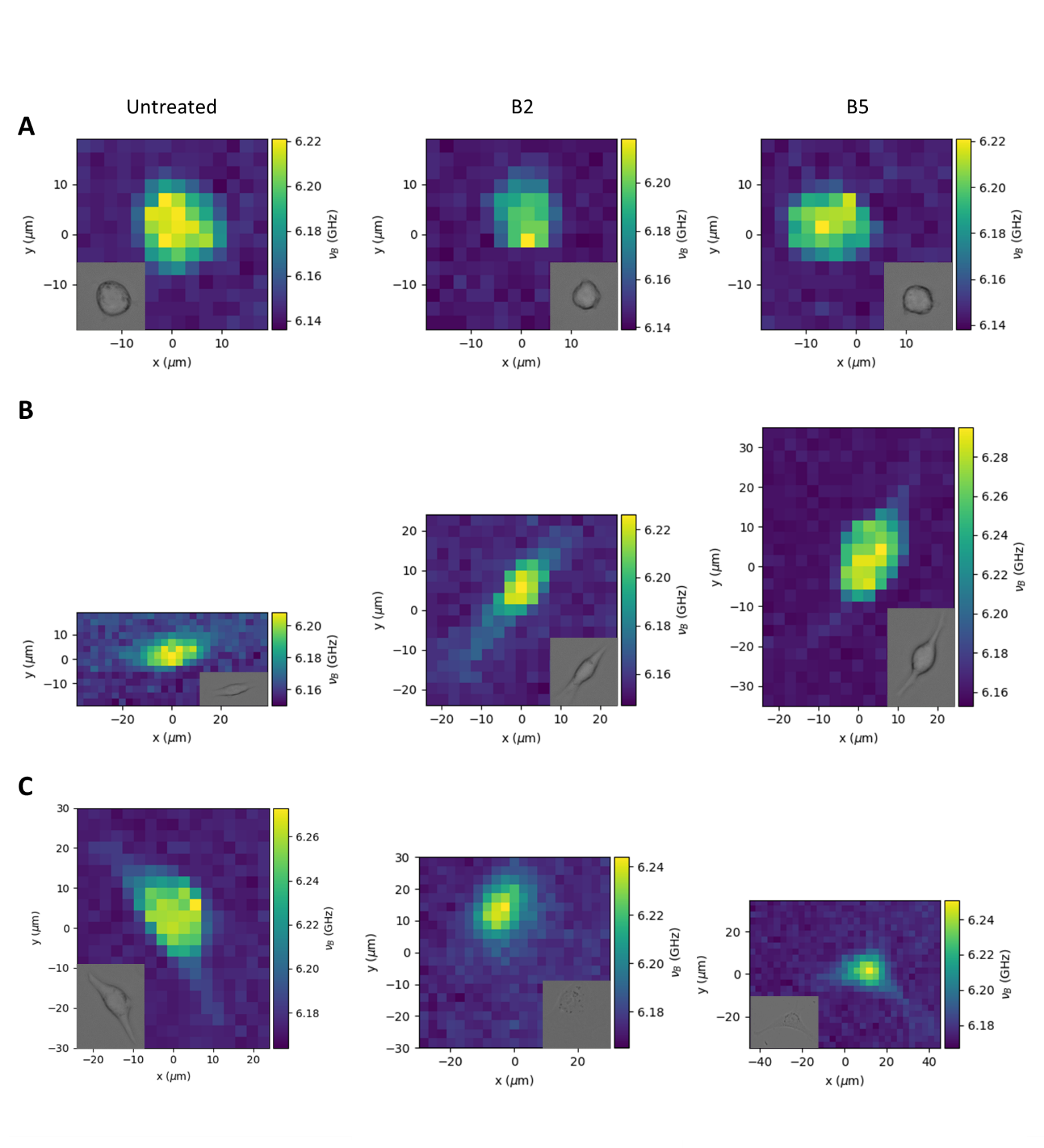


**Figure S11. Cell stiffness is not altered by NaBC1 stimulation on laminin-111.** Representative Brillouin maps of C2C12 myoblasts seeded on PAAm hydrogels with different stiffness functionalized with laminin-111 and stimulated with soluble boron (0.59 and 1.47 mM).


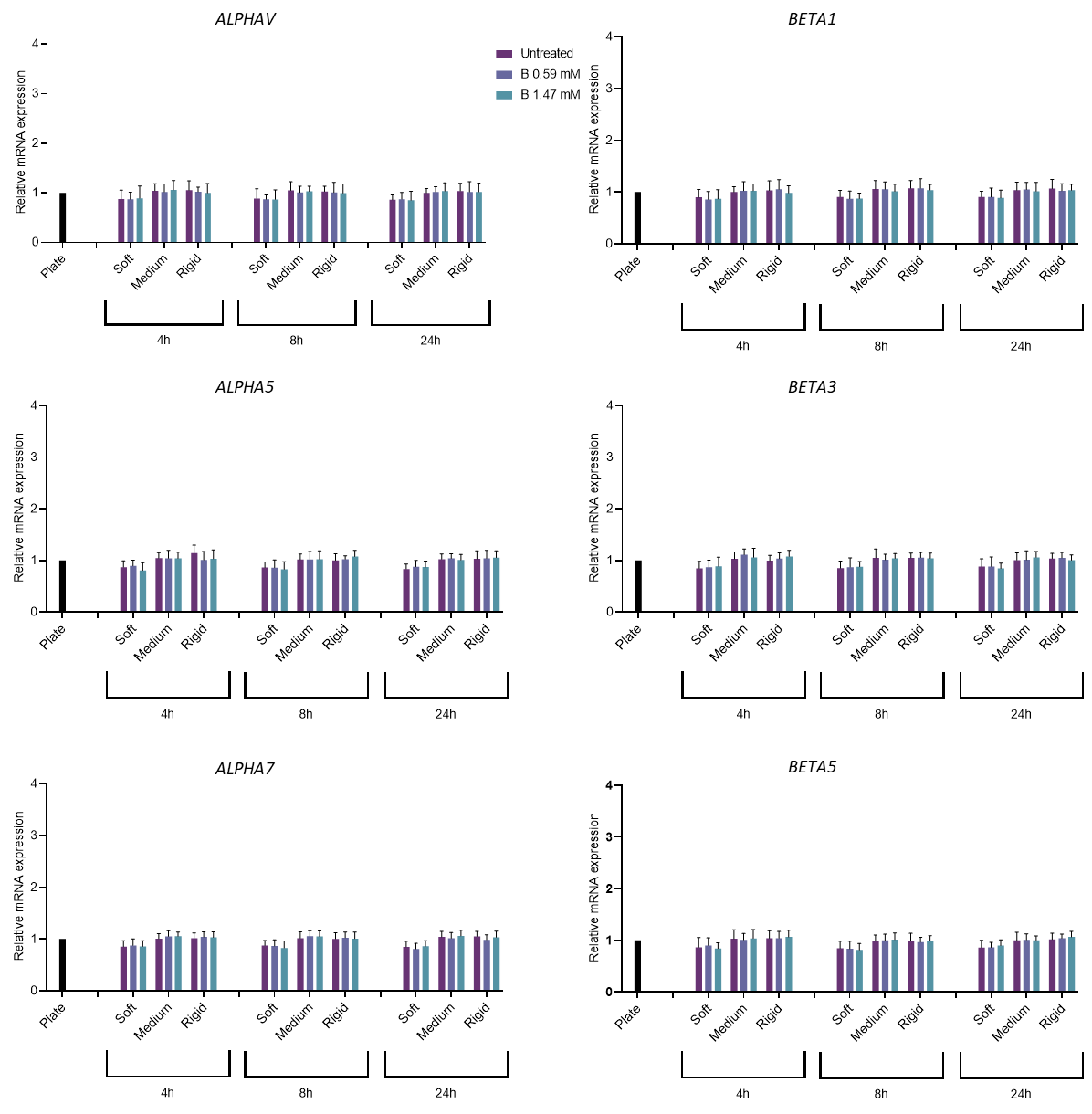


**Figure S12.** **NaBC1 controls intracellular signaling via cooperation with fibronectin-binding integrins.** Quantification of gene expression of cell adhesion-related genes (*ALPHAV*, *ALPHA5*, *ALPHA7*, *BETA1*, *BETA3*, *BETA5* integrins) in C2C12 myoblasts seeded on PAAm hydrogels of different stiffnesses, functionalized with laminin-111 and stimulated with soluble boron (B) (at 0.59 and 1.47 mM) for 4, 8 or 24 hours compared to untreated cells on cell culture plates, as measured by qPCR. *n* = 3 biological replicates with 3 technical replicates. Data are represented as Mean ± Standard Deviation.


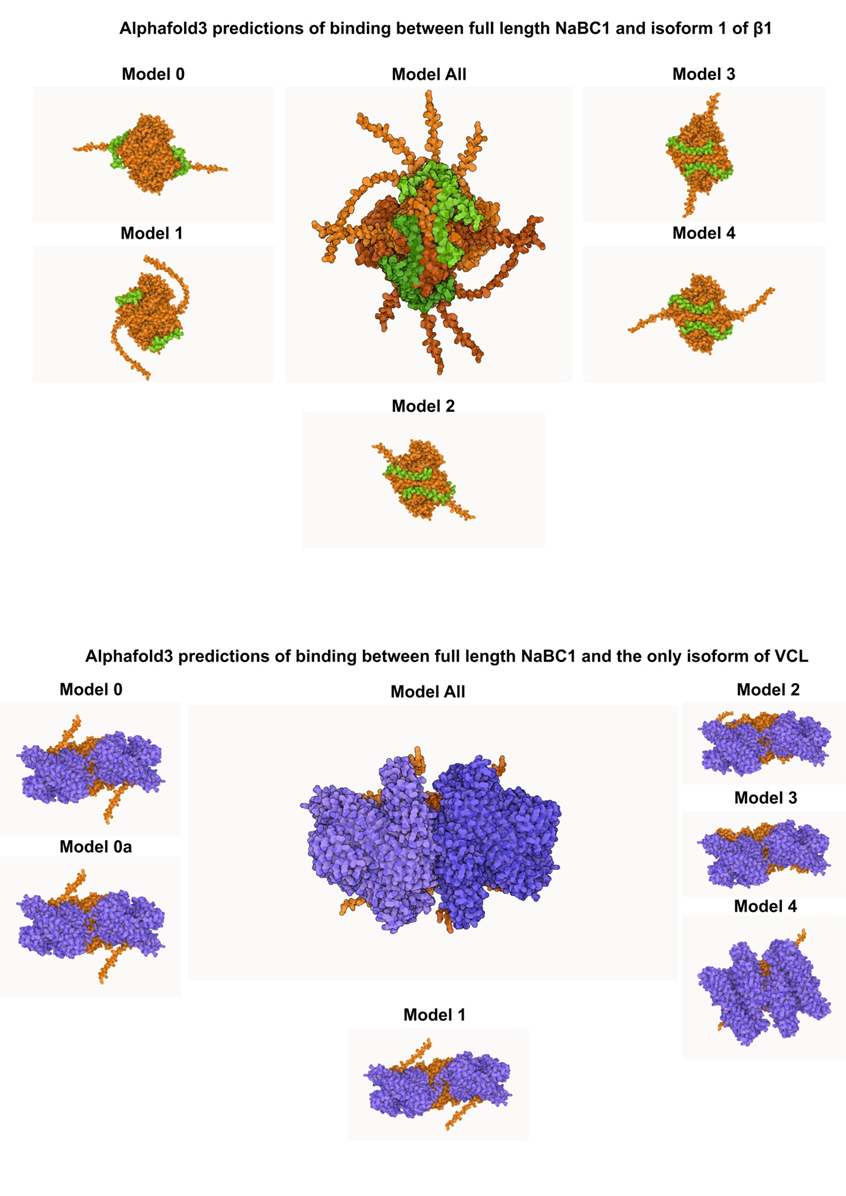


**Figure S13. AlphaFold 3 predicts interactions of NaBC1 with β_1_ integrin and vinculin.** AlphaFold 3 predictions showing different individual models (0-4 for β_1_ integrin and vinculin) as well as their combination (Model All). Up: NaBC1/β_1_ integrin. pLDDT confidence scores (Model 0 to Model 4): 68.5; 68.3; 68.6; 67.5; 68.4; Bottom: NaBC1/vinculin. pLDDT confidence scores (Model 0 to Model 4): 76.3; 76.1; 76.3; 76.2; 75.4;


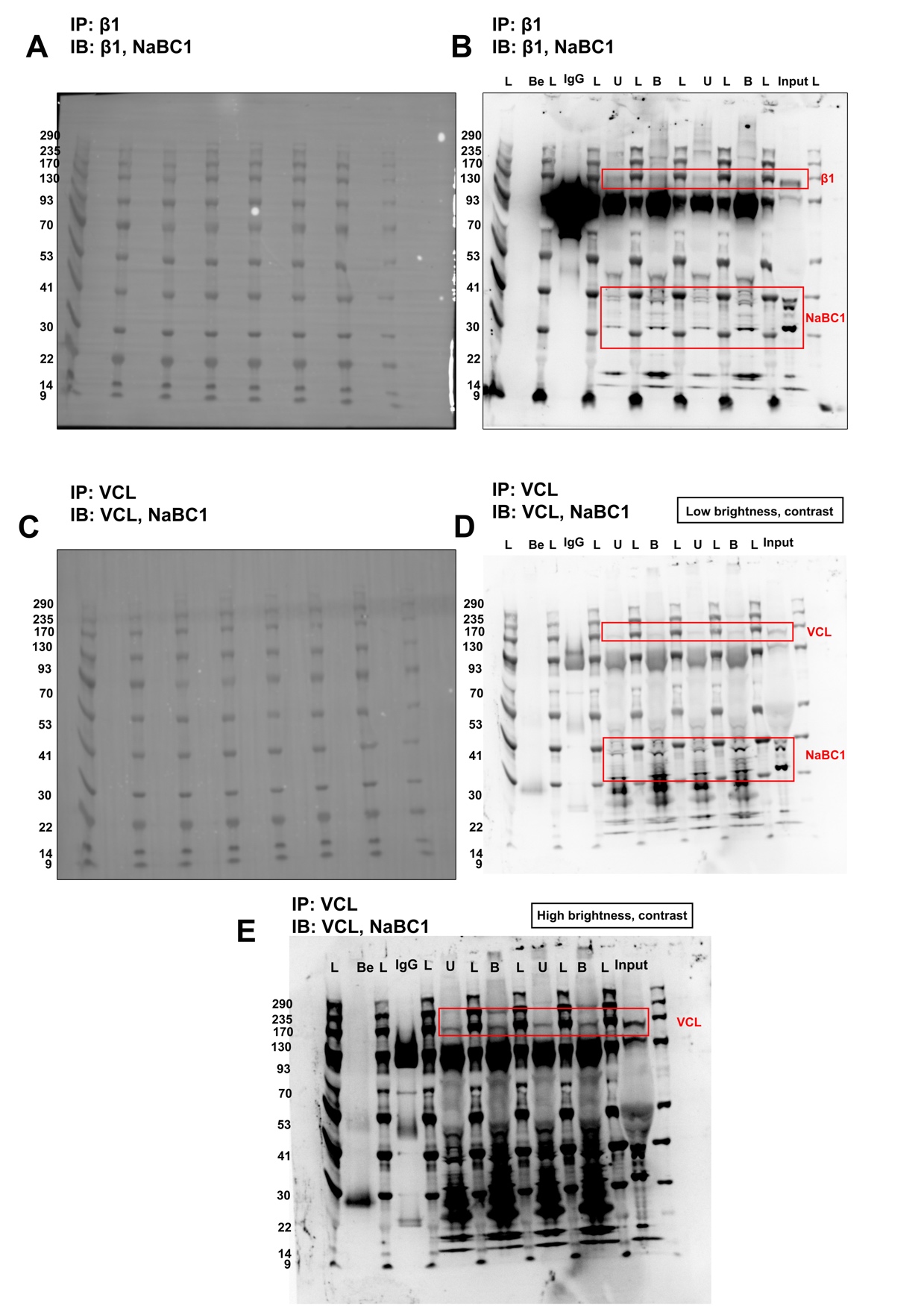


**Figure S14.** **NaBC1 interacts with β_1_ integrin and vinculin on rigid hydrogels and this interaction is amplified in the presence of B.** Uncropped immunoblots showing interaction between (A-B) NaBC1 and β1 integrin as well as between (C-E) NaBC1 and vinculin (VCL). D: Low brightness and contrast image of the immunoblot showing that there was no overlap between protein bands and IgG as well as magnetic beads. L: ladder; Be: beads; U: untreated cells; B: boron-treated cells. Protein ladder PL00003 (Proteintech). All immunoblot experiments were duplicated with identical results.


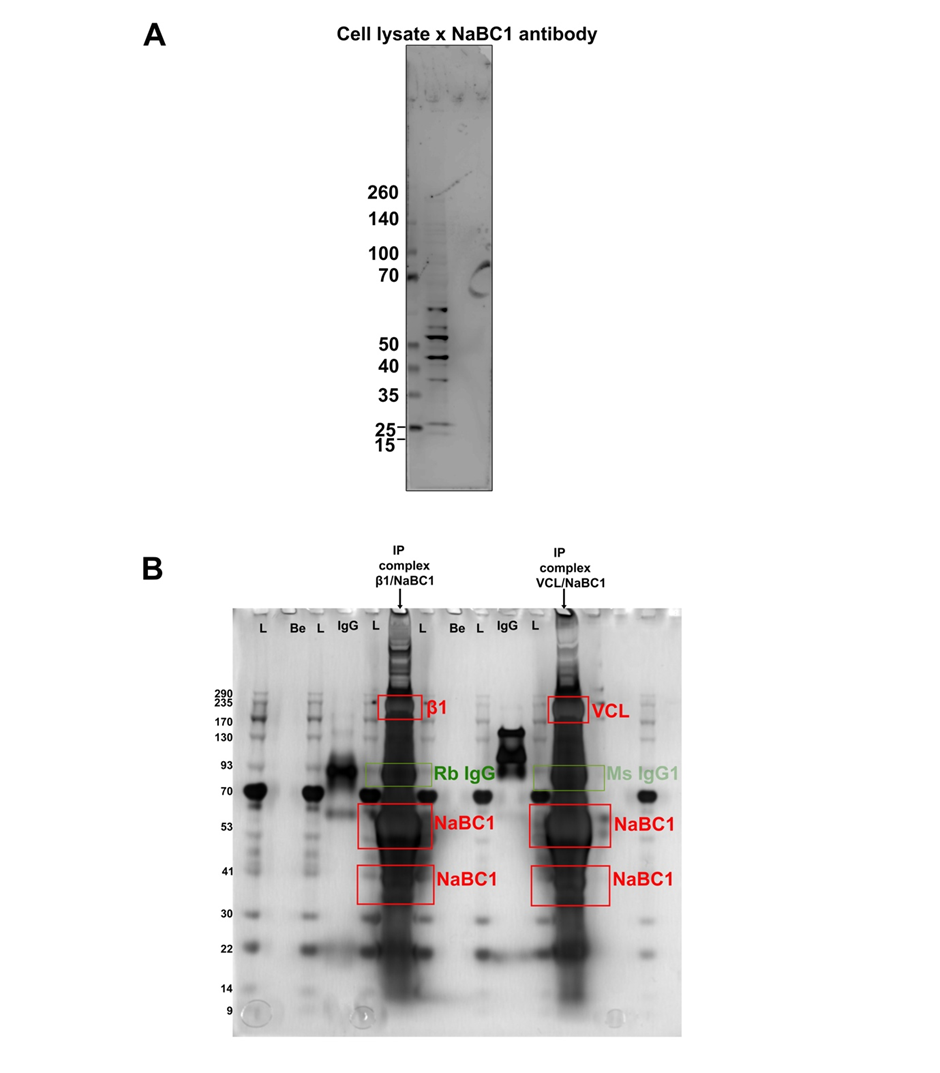


**Figure S15.** **Validation of NaBC1 antibody and assessing individual proteins in immunocomplexes.** A: Proteins in crude cell lysate were separated using SDS-PAGE and immunodetected using NaBC1 to assess if the antibody detects full-length NaBC1 proteins. The antibody did not detect NaBC1 full-length protein, indicating that the protein potentially cleaves into smaller fragments including a 41 kDa intracellular region. B: Silver staining of SDS-PAGE gel showing the presence of different proteins in the immunocomplexes. We observed bands close to 41 kDa, while complements the immunoblot results presented in panel A. We also detected protein bands close to 130 kDa which can be the pulled-down proteins β_1_ integrin or vinculin (VCL). Protein ladder 26634 (ThermoFisher). All immunoblot experiments were duplicated with identical results.


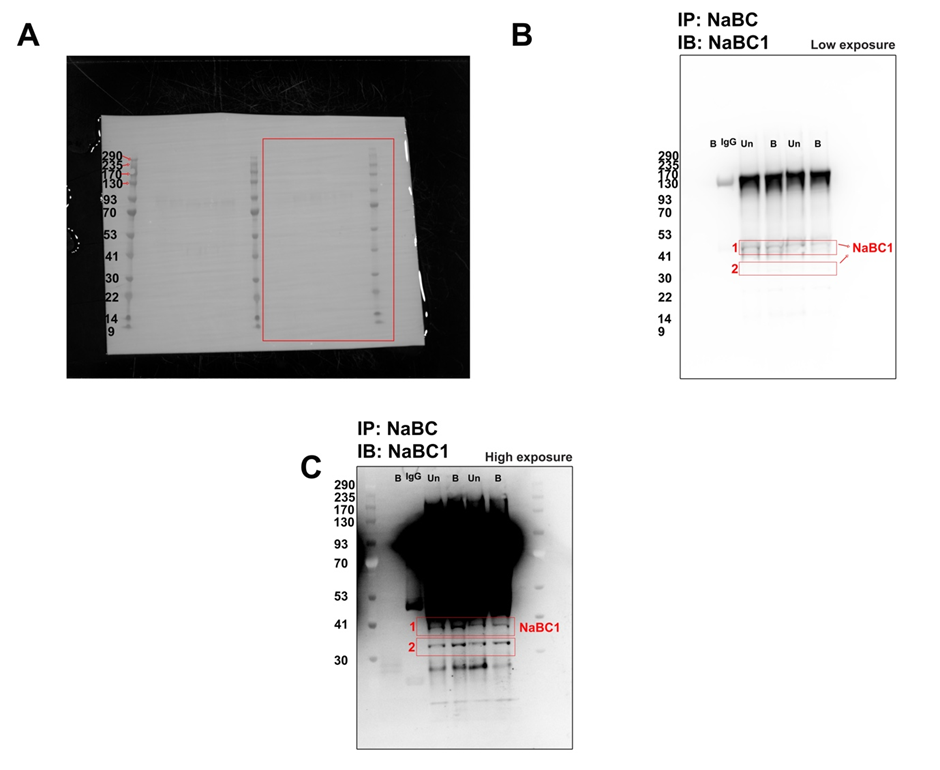


**Figure S16.** **NaBC1 interacts with β_1_ integrin and vinculin on rigid hydrogels and this interaction is amplified in the presence of B.** A-C: Immunoblots showing immunoprecipitation of NaBC1 followed by immunodetection using NaBC1 antibody to assess if the antibody detects full-length NaBC1 protein. The results showed that the full-length protein cleaves into several smaller fragments including the 41 kDa intracellular region which interacts with β_1_ and vinculin. Protein ladder PL00003 (Proteintech).


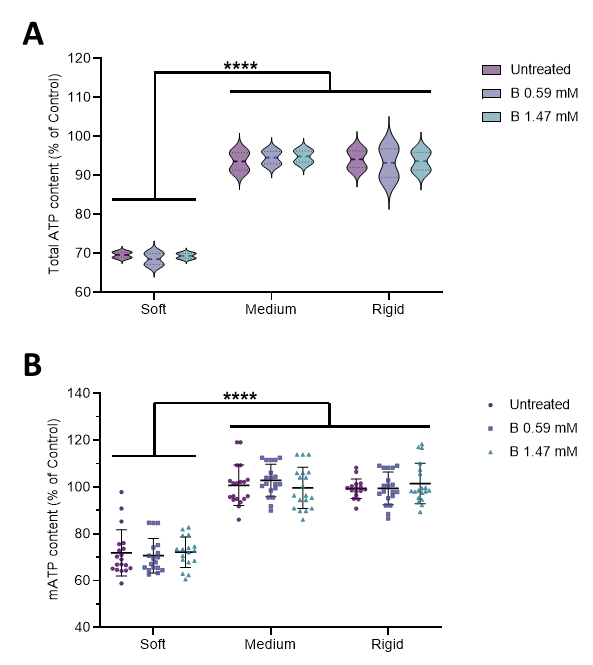


**Figure S17.** **Total and mitochondrial ATP content on laminin-111 is not influenced by NaBC1 or substrate stiffness.** A: Quantification of total content of C2C12 myoblasts seeded on PAAm hydrogels with different stiffness functionalized with laminin-111 and stimulated with soluble boron (0.59 and 1.47 mM). *n*: 3 biological replicates with 3 technical replicates. B: Quantification of mitochondrial content of C2C12 myoblasts seeded on PAAm hydrogels with different stiffness functionalized with laminin-111 and stimulated with soluble boron (0.59 and 1.47 mM). *n*: at least 10 cells from 3 biological replicates. Data are represented as Mean ± Standard Deviation, and differences are considered significant for p ≤ 0.05 using two-way ANOVA (Tukey’s multiple comparisons tests) for multiple comparisons. ****p ≤ 0.0001


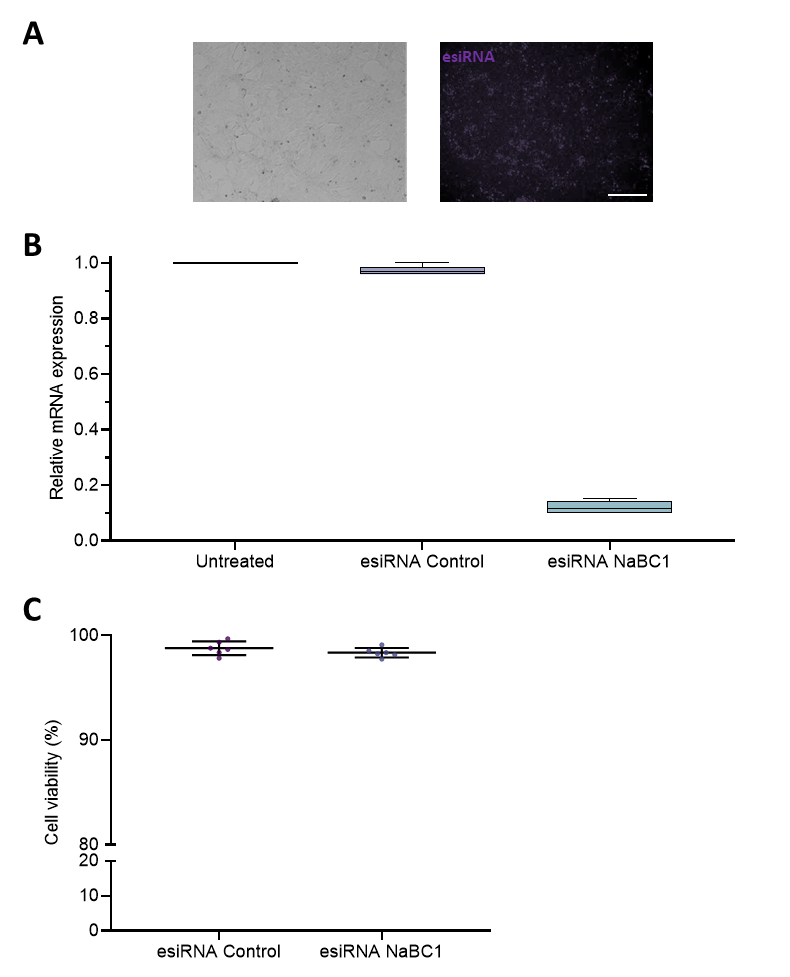


**Figure S18.** **NaBC1 silencing of C2C12 myoblasts does not affect cell viability.** A: Representative images of silenced NaBC1 C2C12 myoblasts. Magenta: Control esiRNA. Scale bar: 100 µm. B: Quantification of mRNA expression of NaBC1 in wild-type and silenced NaBC1 C2C12 myoblasts. *n*: 3 biological replicates with 3 technical replicates. C: Quantification of cell viability of silenced NaBC1 C2C12 myoblasts. *n*: 3 biological replicates with 3 technical replicates. Data are represented as Mean ± Standard Deviation, and differences are considered significant for p ≤ 0.05 using one-way ANOVA (Tukey’s multiple comparisons tests) or *t*-tests for multiple or pairwise comparisons, respectively. ****p ≤ 0.0001


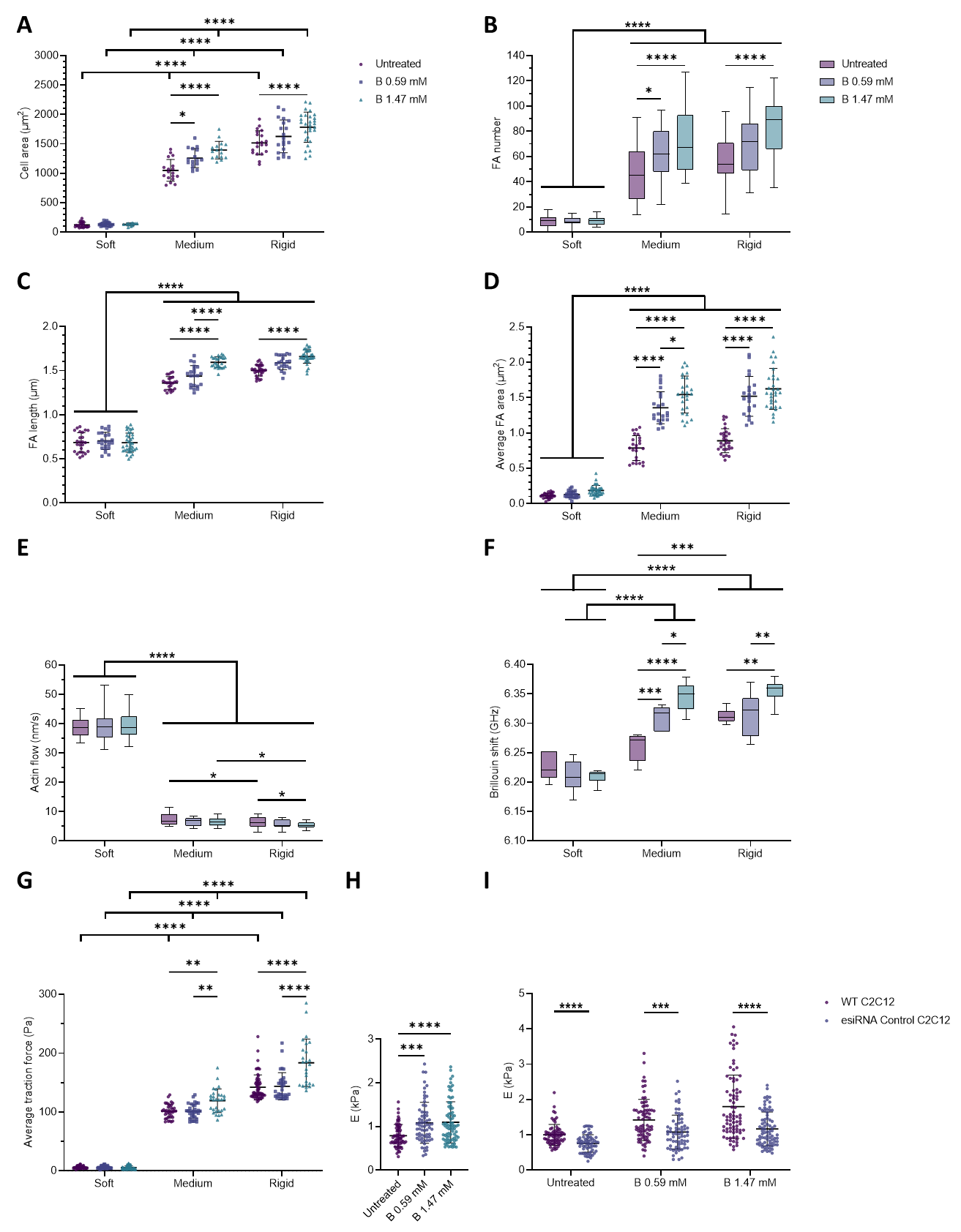


**Figure S19.** **Transfection of C2C12 myoblasts does not affect cell behavior.** The results reported in panels A-D derive from experiments in which C2C12 myoblasts were transfected with esiRNA Control and seeded on PAAm hydrogels of different stiffnesses (soft, medium, and rigid) that were functionalized with fibronectin (FN) and stimulated with soluble boron ions (B) at two different concentrations (0.59 and 1.47 mM). A: Quantification of cell area of Control-silenced C2C12 myoblasts that were treated and cultured as described. *n* = 10 cells from 3 different biological replicates. B: Quantification of the number of focal adhesions in Control-silenced C2C12 myoblasts seeded on PAAm hydrogels with different stiffness functionalized with fibronectin and stimulated with soluble boron (0.59 and 1.47 mM). *n* = 10 cells from 3 different biological replicates. C: Quantification of focal adhesion (FA) length in Control-silenced C2C12 myoblasts that were treated and cultured as described. *n* = 10 cells from 3 different biological replicates. D: Quantification of focal adhesion (FA) average area in Control-silenced C2C12 myoblasts that were treated and cultured as described. *n* = 10 cells from 3 different biological replicates. E: Quantification of actin retrograde flow in Control-silenced C2C12 myoblasts that were treated and cultured as described. *n* = 5 cells with at least 5 different flow areas per cell. F: Quantification of Brillouin shift in Control-silenced C2C12 myoblasts that were treated and cultured as described and imaged by Brillouin microscopy. *n* = 10 cells from 3 different biological replicates. G: Quantification of traction forces exerted by Control-silenced C2C12 myoblasts that were treated and cultured as described. *n* = 30 cells from 10 different locations within each hydrogel from 3 different biological replicates. H: Quantification of cell stiffness by nanoindentation of Control-silenced C2C12 myoblasts seeded on glass coverslips functionalized with FN and stimulated with soluble B (0.59 and 1.47 mM). *n* = 10 cells with 9 indentations on each single cell from 3 different biological replicates. I: Comparison of cell stiffness by nanoindentation of wild type and Control-silenced C2C12 myoblasts seeded on glass coverslips functionalized with FN and stimulated with soluble B (0.59 and 1.47 mM). *n* = 10 cells with 9 indentations on each single cell from 3 different biological replicates. Data are represented as Mean ± Standard Deviation, and differences are considered significant for p ≤ 0.05 using one-way ANOVA or two-way ANOVA (Tukey’s multiple comparisons tests) for multiple comparisons. *p ≤ 0.05, **p ≤ 0.01, ***p ≤ 0.001, ****p ≤ 0.0001


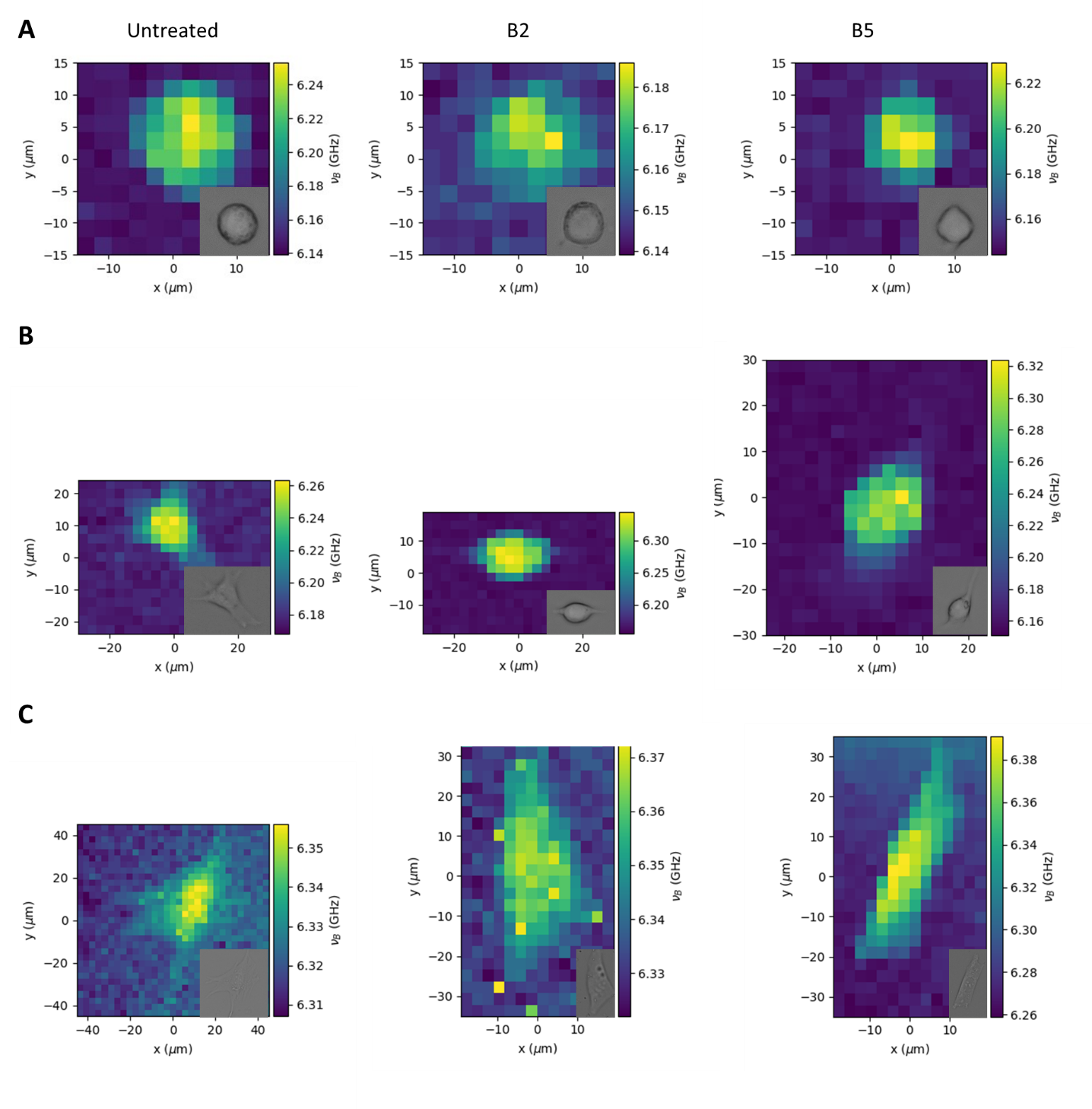


**Figure S20. Control-silencing does not alter cell stiffness on fibronectin-coated substrates.** Representative Brillouin maps of Control-silenced C2C12 myoblasts seeded on PAAm hydrogels with different stiffness functionalized with fibronectin and stimulated with soluble boron (0.59 and 1.47 mM).


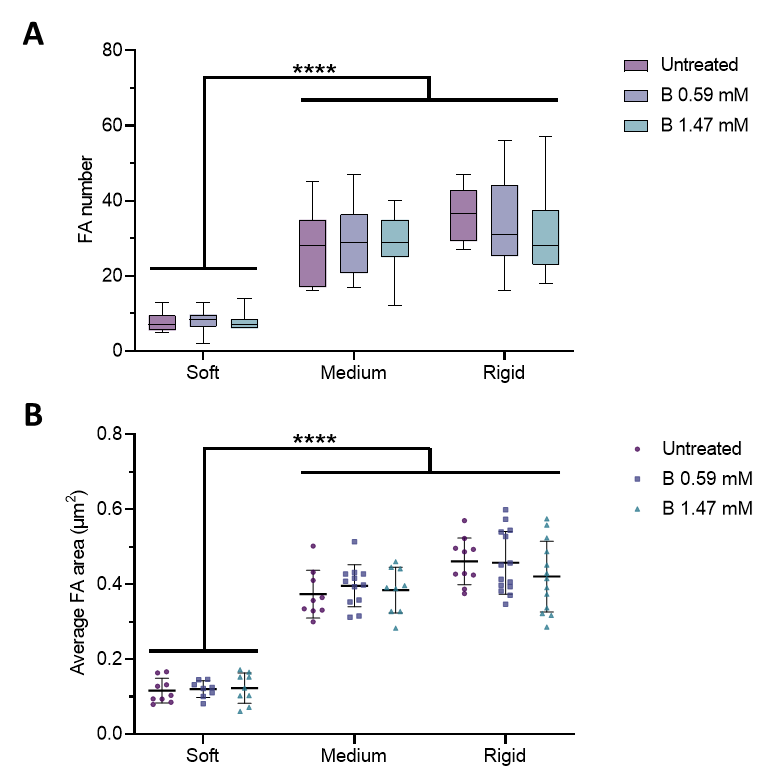


**Figure S21. NaBC1 regulates the stiffness-mediated triggering of formation of FA on fibronectin in NaBC1-silenced myoblasts.** Quantification of the number (A) and average area (B) of focal adhesions in C2C12 myoblasts seeded on PAAm hydrogels with different stiffness functionalized with fibronectin and stimulated with soluble boron (0.59 and 1.47 mM). *n* = 10 cells from 3 different biological replicates. Data are represented as Mean ± Standard Deviation, and differences are considered significant for p ≤ 0.05 using two-way ANOVA (Tukey’s multiple comparisons tests) for multiple comparisons. ****p ≤ 0.0001


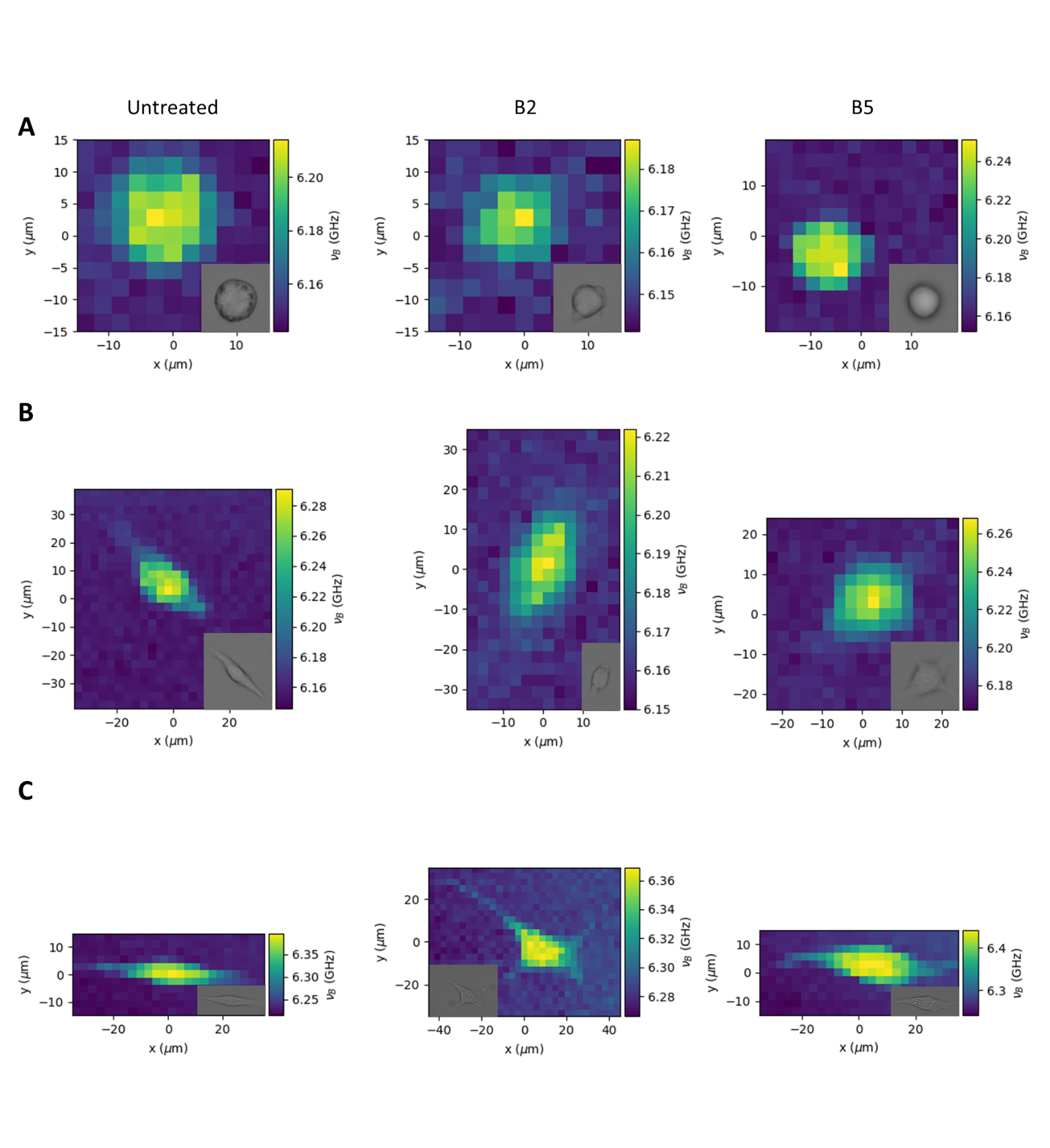


**Figure S22. NaBC1-silencing does not alter cell stiffness on fibronectin-coated substrates.** Representative Brillouin maps of NaBC1-silenced C2C12 myoblasts seeded on PAAm hydrogels with different stiffness functionalized with fibronectin and stimulated with soluble boron (0.59 and 1.47 mM).


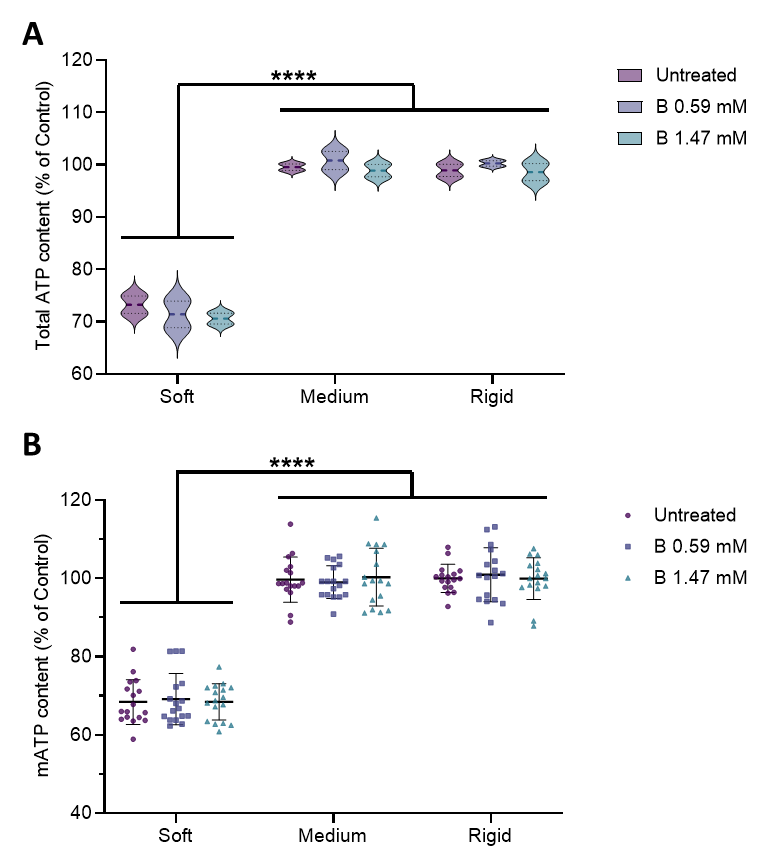


**Figure S23.** **NaBC1 stimulation is essential for stiffness-mediated triggering of total and mitochondrial ATP content on fibronectin substrates.** A: Quantification of total content of NaBC1-silenced C2C12 myoblasts seeded on PAAm hydrogels with different stiffness functionalized with fibronectin and stimulated with soluble boron (0.59 and 1.47 mM). *n*: 3 biological replicates with 3 technical replicates. B: Quantification of mitochondrial content of NaBC1-silenced C2C12 myoblasts seeded on PAAm hydrogels with different stiffness functionalized with fibronectin and stimulated with soluble boron (0.59 and 1.47 mM). *n*: at least 10 cells from 3 biological replicates. Data are represented as Mean ± Standard Deviation, and differences are considered significant for p ≤ 0.05 using two-way ANOVA (Tukey’s multiple comparisons tests) for multiple comparisons. ****p ≤ 0.0001


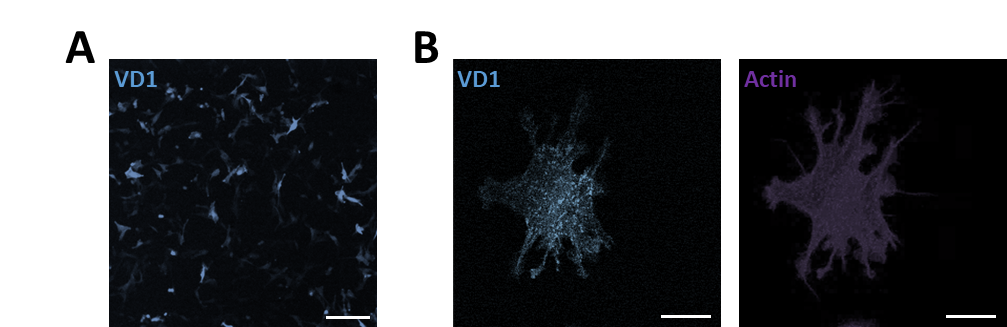


**Figure S24.** **Transfection of C2C12 myoblasts with the VD1 plasmid.** A: Representative image of C2C12 myoblasts transfected with the VD1 plasmid. Cyan: VD1 plasmid. Scale bar: 100 µm. B: Representative images of C2C12 myoblasts transfected with the VD1 and LifeAct plasmids. Cyan: VD1 plasmid; Magenta: LifeAct plasmid. Scale bar: 20 µm.


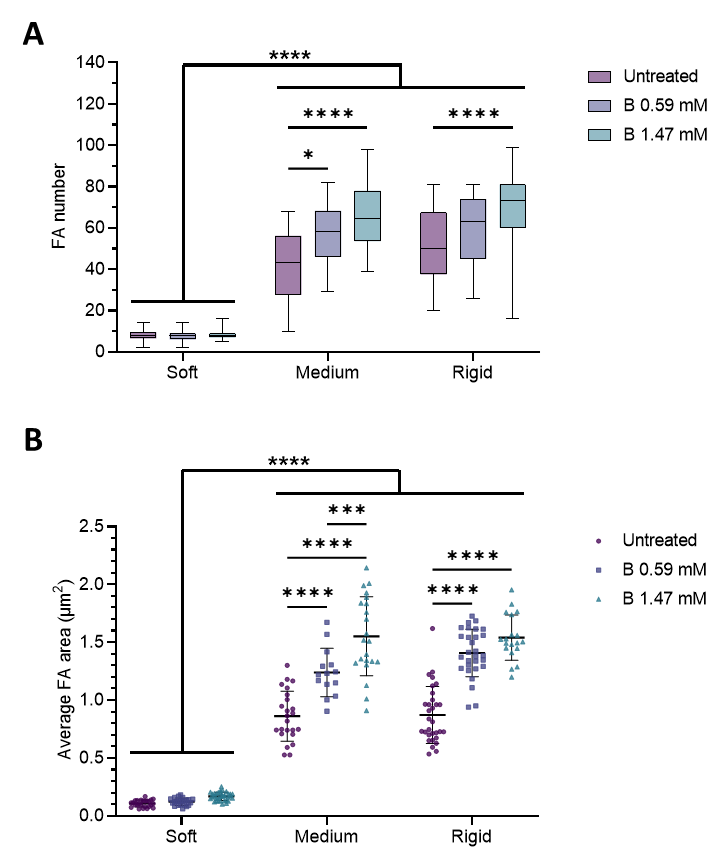


**Figure S25. NaBC1 and stiffness-mediated triggering of formation of FA in myoblasts on fibronectin substrates is independent of the talin-vinculin binding.** Quantification of the number (A) and average area (B) of focal adhesions in C2C12 myoblasts transfected with the VD1 plasmid seeded on PAAm hydrogels with different stiffness functionalized with fibronectin and stimulated with soluble boron (0.59 and 1.47 mM). *n* = 10 cells from 3 different biological replicates. Data are represented as Mean ± Standard Deviation, and differences are considered significant for p ≤ 0.05 using two-way ANOVA (Tukey’s multiple comparisons tests) for multiple comparisons. *p ≤ 0.05, ****p ≤ 0.0001


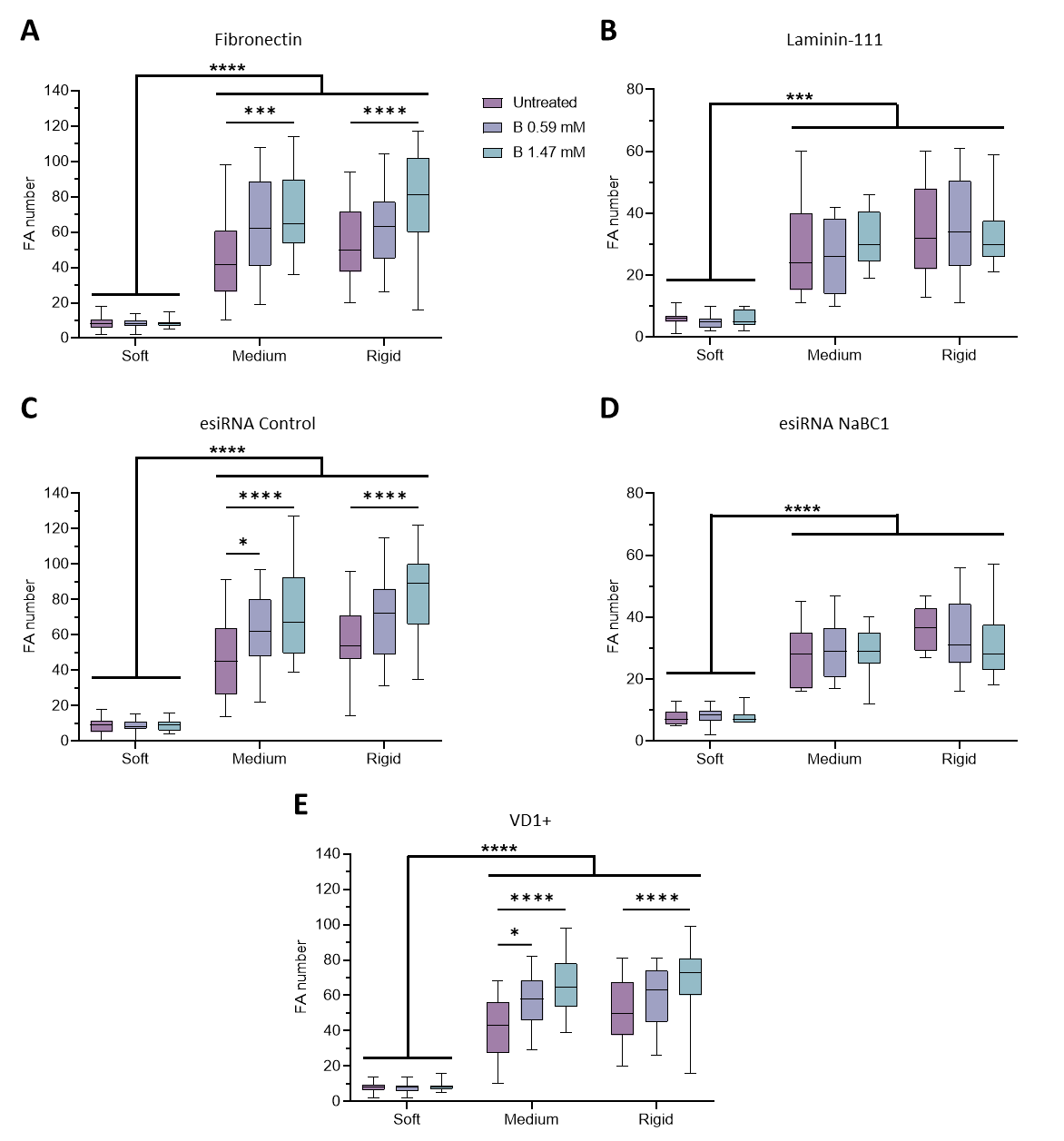


**Figure S26. Comparison of the number of FA in C2C12 myoblasts.** Quantification of the number of focal adhesions in wild type C2C12 myoblasts (A-B), Control-silenced C2C12 myoblasts (C), NaBC1-silenced C2C12 myoblasts (D) or C2C12 myoblasts transfected with the VD1 plasmid (E) seeded on PAAm hydrogels with different stiffness functionalized with fibronectin (A, C, D and E) or laminin-111 (B) and stimulated with soluble boron (0.59 and 1.47 mM). *n* = 10 cells from 3 different biological replicates. Data are represented as Mean ± Standard Deviation, and differences are considered significant for p ≤ 0.05 using two-way ANOVA (Tukey’s multiple comparisons tests) for multiple comparisons. *p ≤ 0.05, ***p ≤ 0.001, ****p ≤ 0.0001

**
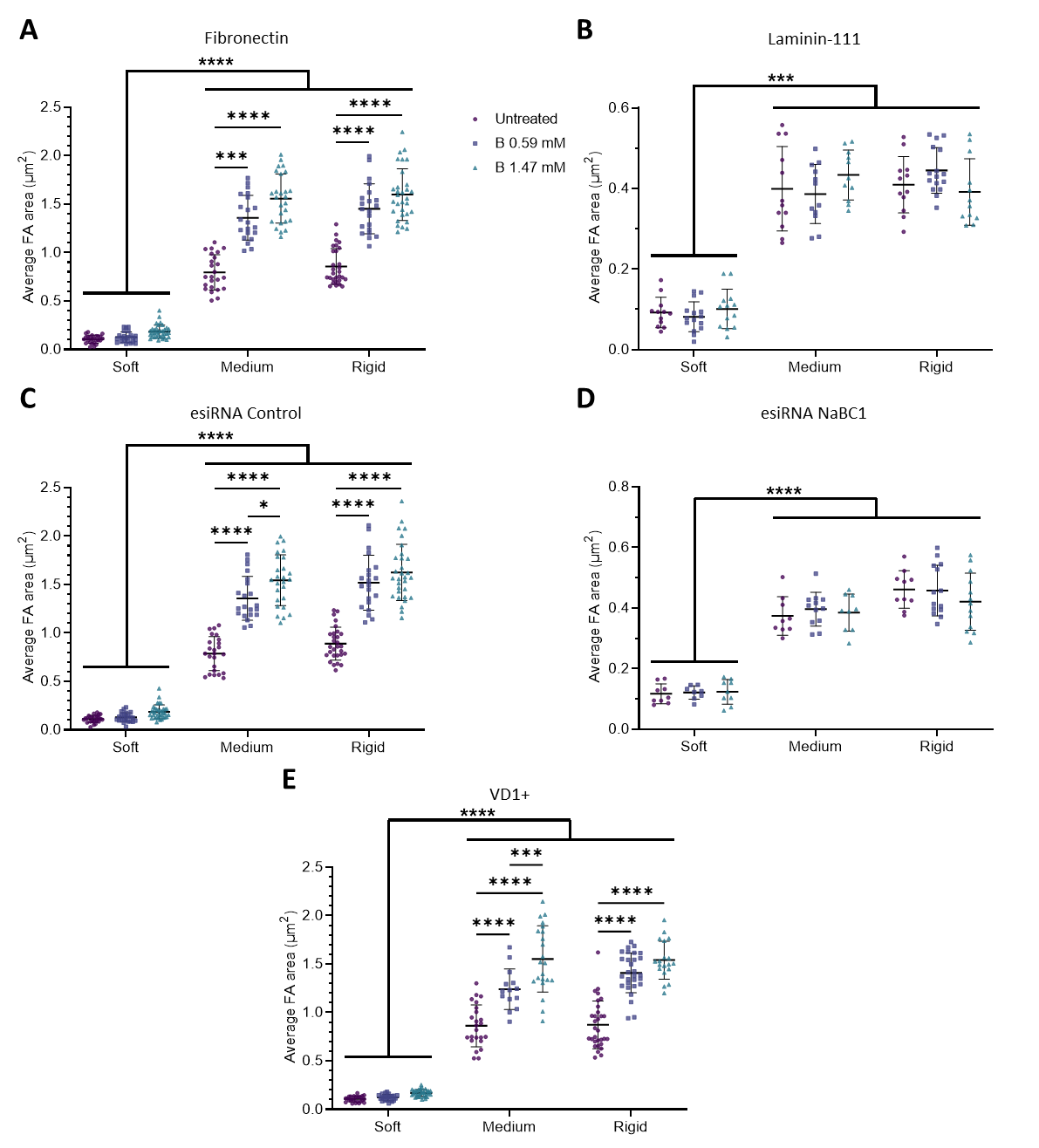
**

**Figure S27. Comparison of the average FA area in C2C12 myoblasts.** Quantification of the average area of focal adhesions in wild type C2C12 myoblasts (A-B), Control-silenced C2C12 myoblasts (C), NaBC1-silenced C2C12 myoblasts (D) or C2C12 myoblasts transfected with the VD1 plasmid (E) seeded on PAAm hydrogels with different stiffness functionalized with fibronectin (A, C, D and E) or laminin-111 (B) and stimulated with soluble boron (0.59 and 1.47 mM). *n* = 10 cells from 3 different biological replicates. Data are represented as Mean ± Standard Deviation, and differences are considered significant for p ≤ 0.05 using two-way ANOVA (Tukey’s multiple comparisons tests) for multiple comparisons. *p ≤ 0.05, ***p ≤ 0.001, ****p ≤ 0.0001

**
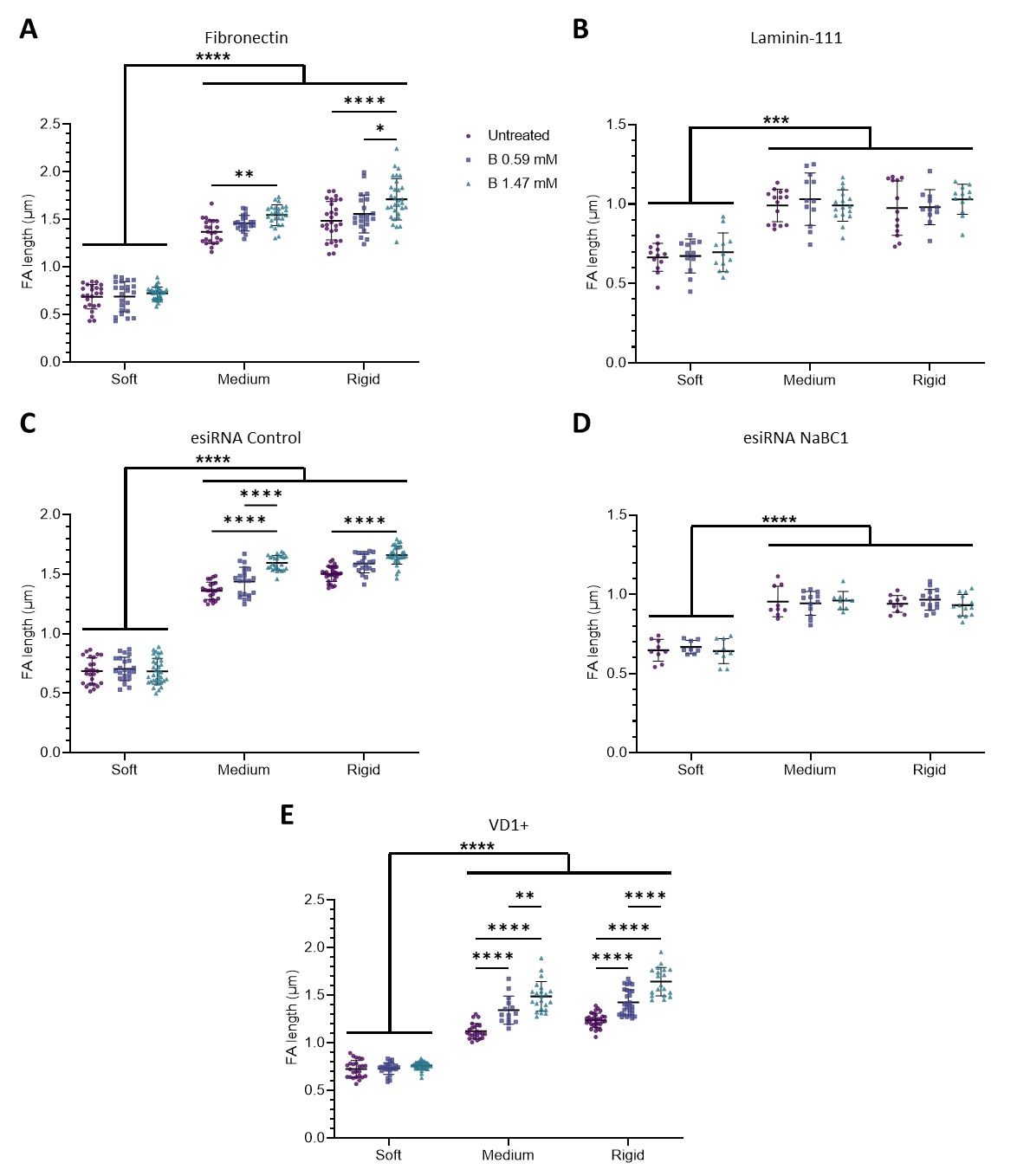
**

**Figure S28. Comparison of FA length in C2C12 myoblasts.** Quantification of the length of focal adhesions in wild type C2C12 myoblasts (A-B), Control-silenced C2C12 myoblasts (C), NaBC1-silenced C2C12 myoblasts (D) or C2C12 myoblasts transfected with the VD1 plasmid (E) seeded on PAAm hydrogels with different stiffness functionalized with fibronectin (A, C, D and E) or laminin-111 (B) and stimulated with soluble boron (0.59 and 1.47 mM). *n* = 10 cells from 3 different biological replicates. Data are represented as Mean ± Standard Deviation, and differences are considered significant for p ≤ 0.05 using two-way ANOVA (Tukey’s multiple comparisons tests) for multiple comparisons. *p ≤ 0.05, **p ≤ 0.01, ***p ≤ 0.001, ****p ≤ 0.0001
